# Supplementary material for: Collective chiroptical activity through the interplay of excitonic and charge-transfer effects in localized plasmonic fields
Source: Nat Commun. 2024 Jun 6;15:4846. doi: 10.1038/s41467-024-49086-3 (PMC11156920; doi:10.1038/s41467-024-49086-3)
Supplement: Supplementary file 1 — Supplementary Information [file 41467_2024_49086_MOESM1_ESM.pdf]

Supplementary Information

Collective chiroptical activity through the interplay of excitonic and charge-transfer effects in localized plasmonic fields

Huacheng Li,[1,2,3,13] Xin Xu,[1,2,3,13] Rongcheng Guan,[1,2,3] Artur Movsesyan,[4,5] Zhenni Lu,[1,6] Qiliang Xu,[1,6] Ziyun Jiang,[1,2,3] Yurong Yang,[1,2,3] Majid Khan,[1,2,3] Jin Wen,[1,3] Hongwei Wu,[1,6] Santiago de la Moya,[7] Gil Markovich,[8] Huatian Hu,[9] Zhiming Wang,[4] Qiang Guo,[10] Tao Yi,[1,6] Alexander O. Govorov,[4,5,*] Zhiyong Tang,[11,12] & Xiang Lan[1,2,3,*]

[1]State Key Laboratory for Modification of Chemical Fibers and Polymer Materials, Donghua University, Shanghai 201620, China

[2]Center for Advanced Low-dimension Materials, Donghua University, Shanghai 201620, China

[3]College of Materials Science and Engineering, Donghua University, Shanghai 201620, China

[4]Institute of Fundamental and Frontier Sciences, University of Electronic Science and Technology of China, Chengdu 610054, China

[5]Department of Physics and Astronomy and Nanoscale and Quantum Phenomena Institute, Ohio University, Athens, Ohio 45701, United States

[6]College of Chemistry and Chemical Engineering, Donghua University, Shanghai 201620, China

[7]Departamento de Química Orgánica, Facultad de Ciencias Químicas, Universidad Complutense de Madrid, Ciudad Universitaria s/n, Madrid 28040, Spain

[8]School of Chemistry, Raymond and Beverly Sackler Faculty of Exact Sciences, Tel Aviv University, Tel Aviv 69978, Israel

[9]Center for Biomolecular Nanotechnologies, Istituto Italiano di Tecnologia, Via Barsanti 14, Arnesano 73010, LE, Italy

[10]State Key Laboratory of Protein and Plant Gene Research, Center for Life Sciences, Academy for Advanced Interdisciplinary Studies, School of Life Sciences, Peking University, Beijing 100871, China

[11]CAS Key Laboratory of Nanosystem and Hierarchical Fabrication & CAS Center for Excellence in Nanoscience, National Center for Nanoscience and Technology, Beijing 100190, China

[12]University of Chinese Academy of Sciences, Beijing 100049, China

[13]These authors contributed equally: Huacheng Li, Xin Xu

[*]e-mail: govorov@ohio.edu, xlan@dhu.edu.cn

Table of Contents

Supplementary Notes

1. Förster resonance energy transfer (FRET) and site-specificity........................S15

2. Stability test of chromophore-DNA conjugates..S16

3. Aggregation test of chromophore-DNA conjugates S18

4. Quantification of surface concentration of chromophore-DNA conjugates ...S20

5. Additional experimental data...S22

6. Theoretical analysis..S35

Supplementary Figures...S3-S42

Supplementary Discussion S40

Supplementary Tables S43

Supplementary References...S46

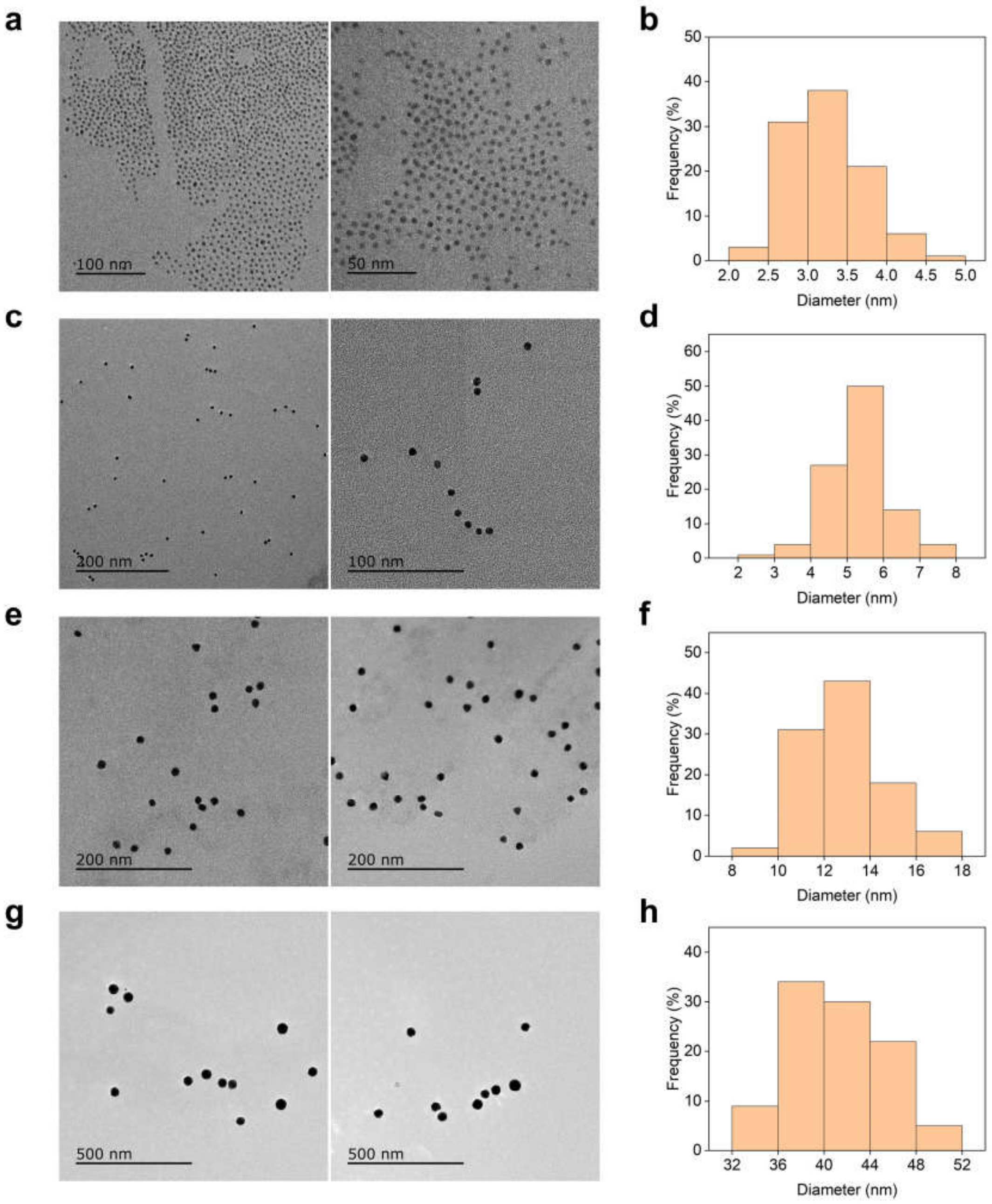

Supplementary Fig. 1. TEM images and size distribution of Au nanoparticles (AuNPs). a, b, Namely 3 nm AuNPs (diameter = 3.2 ± 0.5 nm). **c, d,** Namely 5 nm AuNPs (diameter = 5.4 ± 0.9 nm). **e, f,** Namely 13 nm AuNPs (diameter = 13 ± 2 nm). **g, h,** Namely 40 nm AuNPs (diameter = 41 ± 4 nm). At the 0.05 significance level, the population median (3.2 nm, 5.4 nm, 13 nm and 41 nm, respectively) is not significantly different from the test median (3 nm, 5 nm, 13 nm and 40 nm, correspondingly) using one-sample Student's *t*-test or one-sample Wilcoxon signed rank test (sampling number = 100 for differently sized AuNPs).

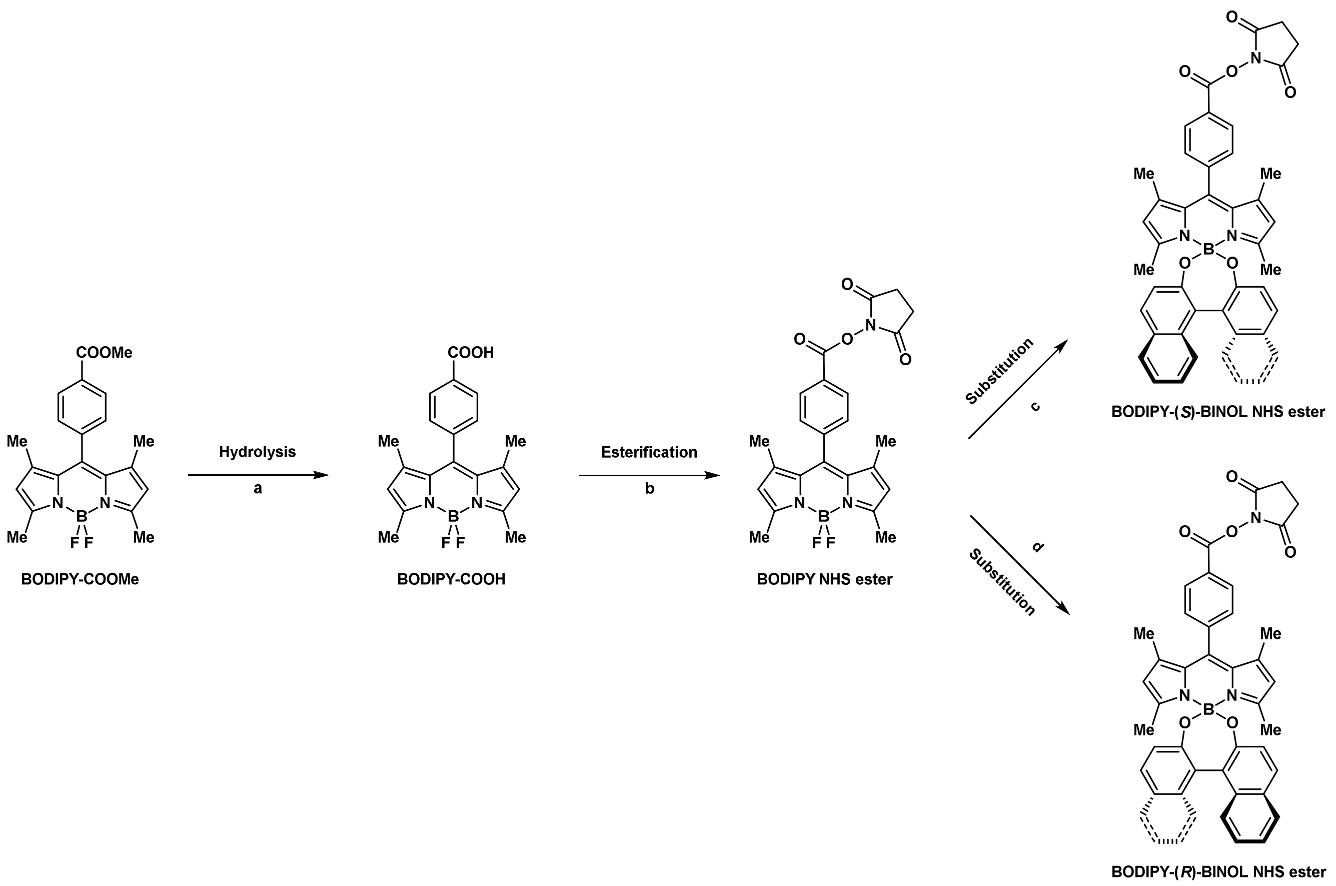

Supplementary Fig. 2. Synthetic routes for BODIPY-(*S*)-BINOL NHS ester and BODIPY-(*R*)-BINOL NHS ester. Reaction conditions: **a,** KOH, H_2O, isopropanol, HCl, room temperature, TLC monitored. **b,** *N*-hydroxy-succinimide, 1-(3-dimethylaminopropyl)-3-ethylcarbodiimide hydrochloride, dry CH_2Cl_2, room temperature, TLC monitored. **c,** 1,1'-binaphthyl-2,2'-diol ((*S*)-BINOL), Et_2AlCl, dry CH_2Cl_2, room temperature, 2 h. **d,** 1,1'-binaphthyl-2,2'-diol ((*R*)-BINOL), Et_2AlCl, dry CH_2Cl_2, room temperature, 2 h.

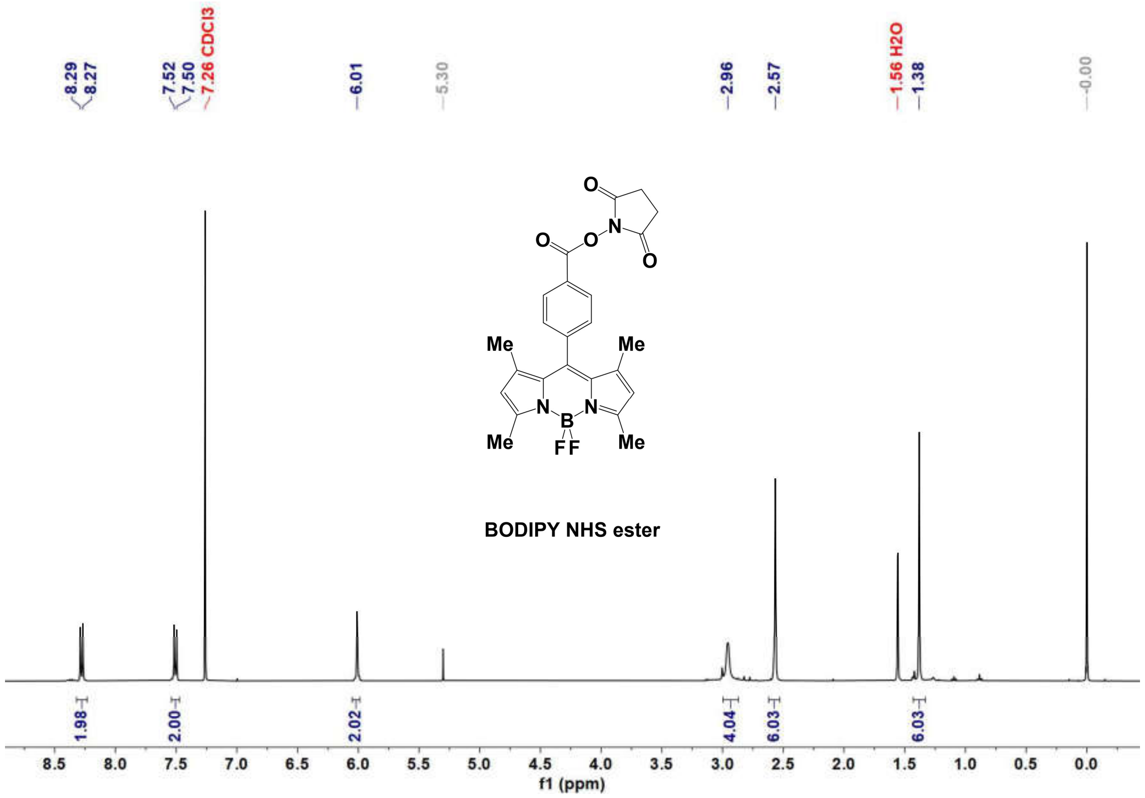

Supplementary Fig. 3. ^{1}H NMR spectrum of BODIPY NHS ester (400 MHz, Chloroform-*d*).

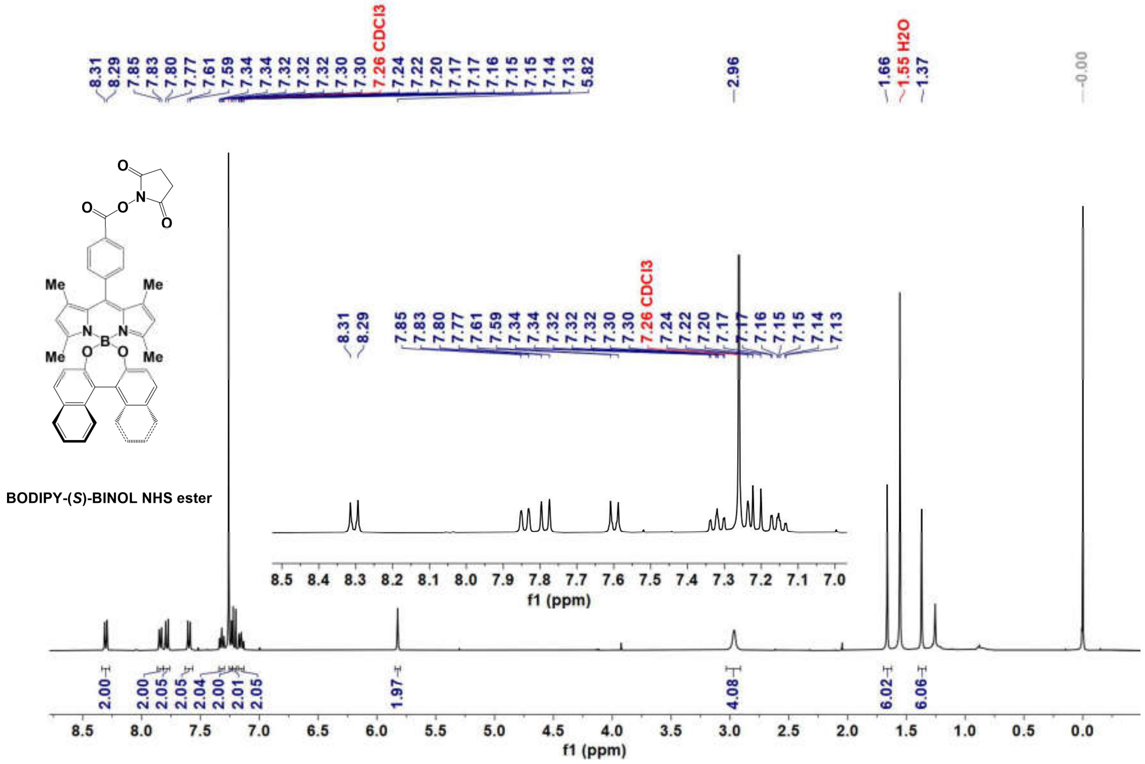

Supplementary Fig. 4. ^{1}H NMR spectrum of BODIPY-(*S*)-BINOL NHS ester (400 MHz, Chloroform-*d*).

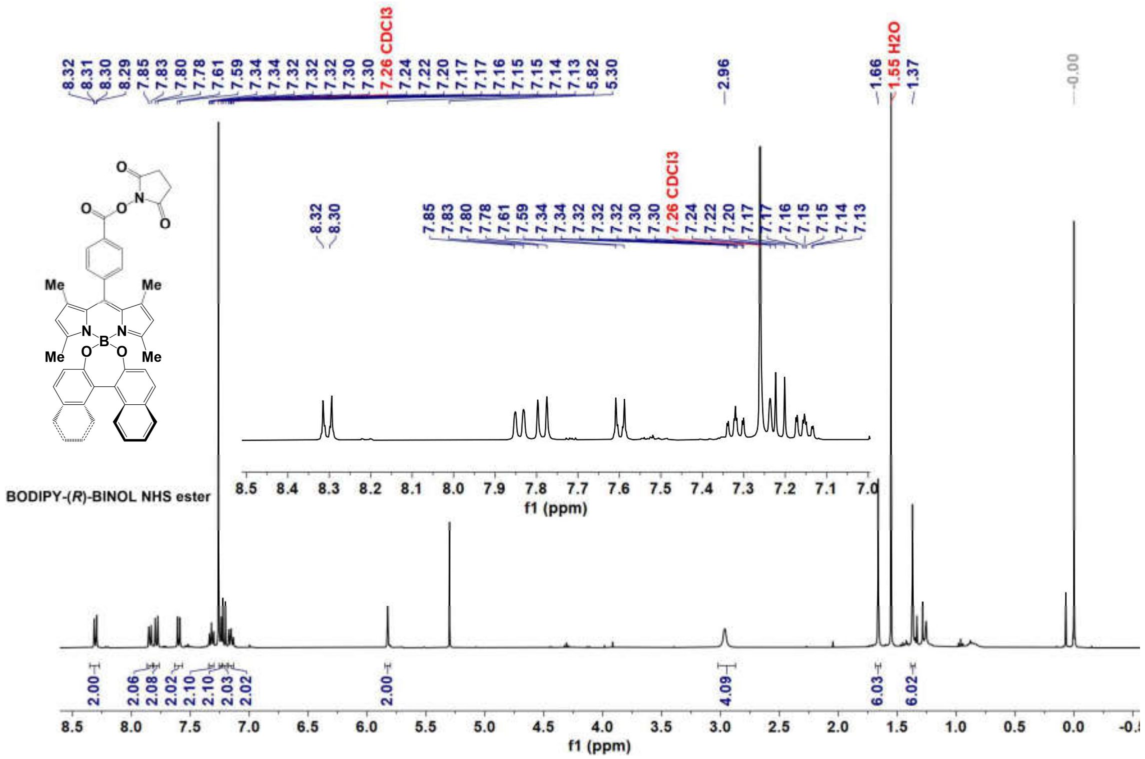

Supplementary Fig. 5. **^{1}H NMR spectrum of BODIPY-(*R*)-BINOL NHS ester (400 MHz, Chloroform-*d*).**

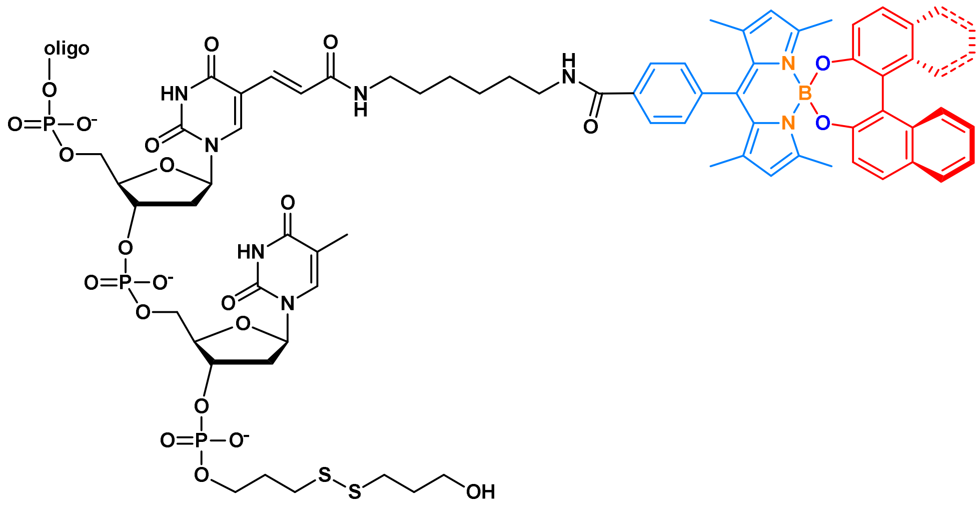

Supplementary Fig. 6. Molecular structure diagram of cBDP-DNA conjugates, taking cBDP (*S*)-DNA (2nt) (*S* refers to (*S*)-BINOL) as an example. The cBDP molecule is conjugated to side chain of thymine by amide bond. The DNA backbone 3’ end is modified by disulfide bond for AuNP surface modification. The electron-withdrawing BODIPY core is highlighted in blue, and the electron-donating (*S*)-BINOL moiety is highlighted in red.

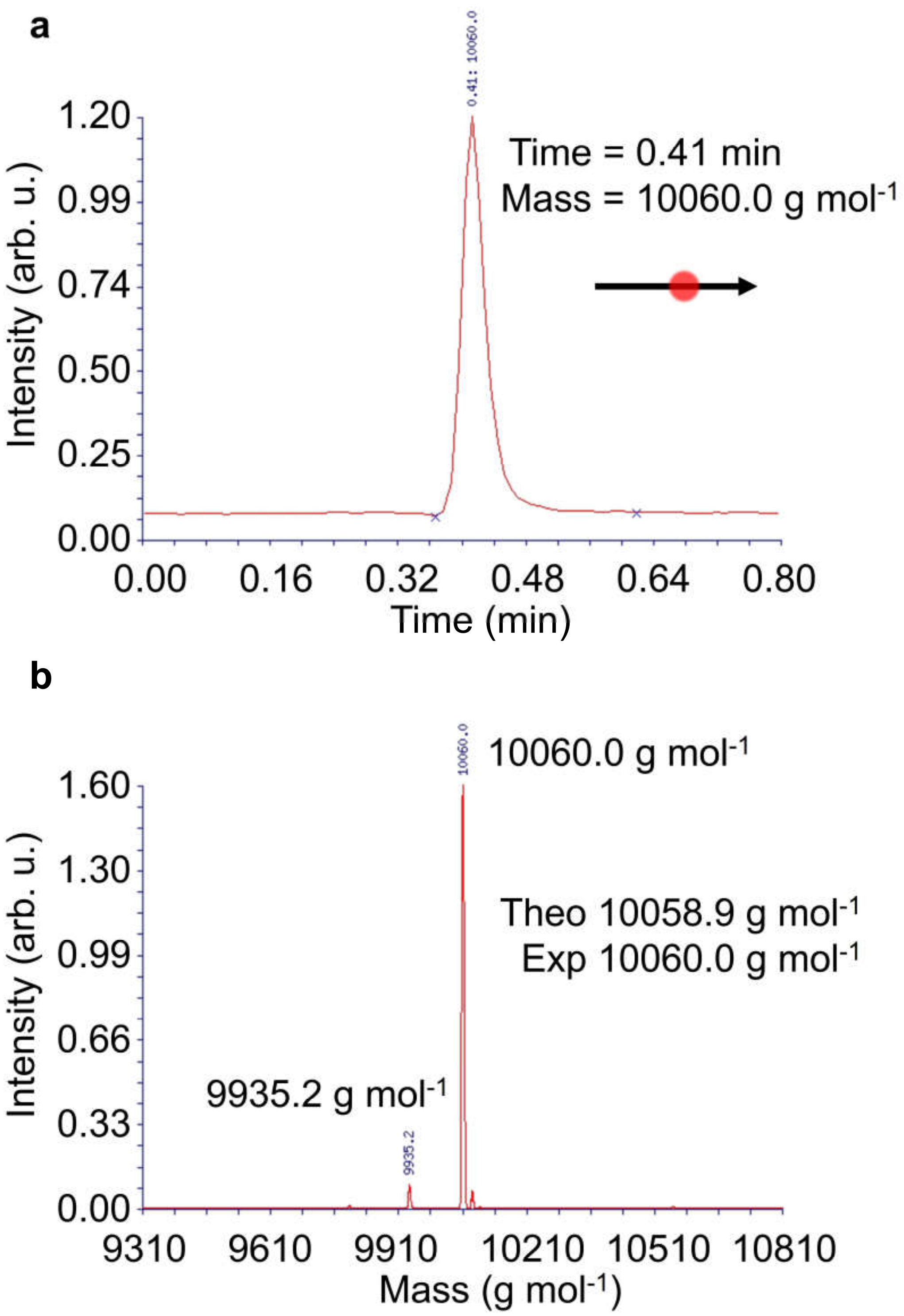

Supplementary Fig. 7. Liquid chromatography mass spectrometry (LCMS) chromatogram (a) and electrospray ionization (ESI) mass spectrum (b) of cBDP (*S*)-DNA (14nt). The theoretical values (Theo) and experimental mass (Exp) are labelled, confirming the success synthesis and purity of cBDP (*S*)-DNA (14nt). The cBDP is represented by a red spot and the DNA backbone is represented by a black arrow.

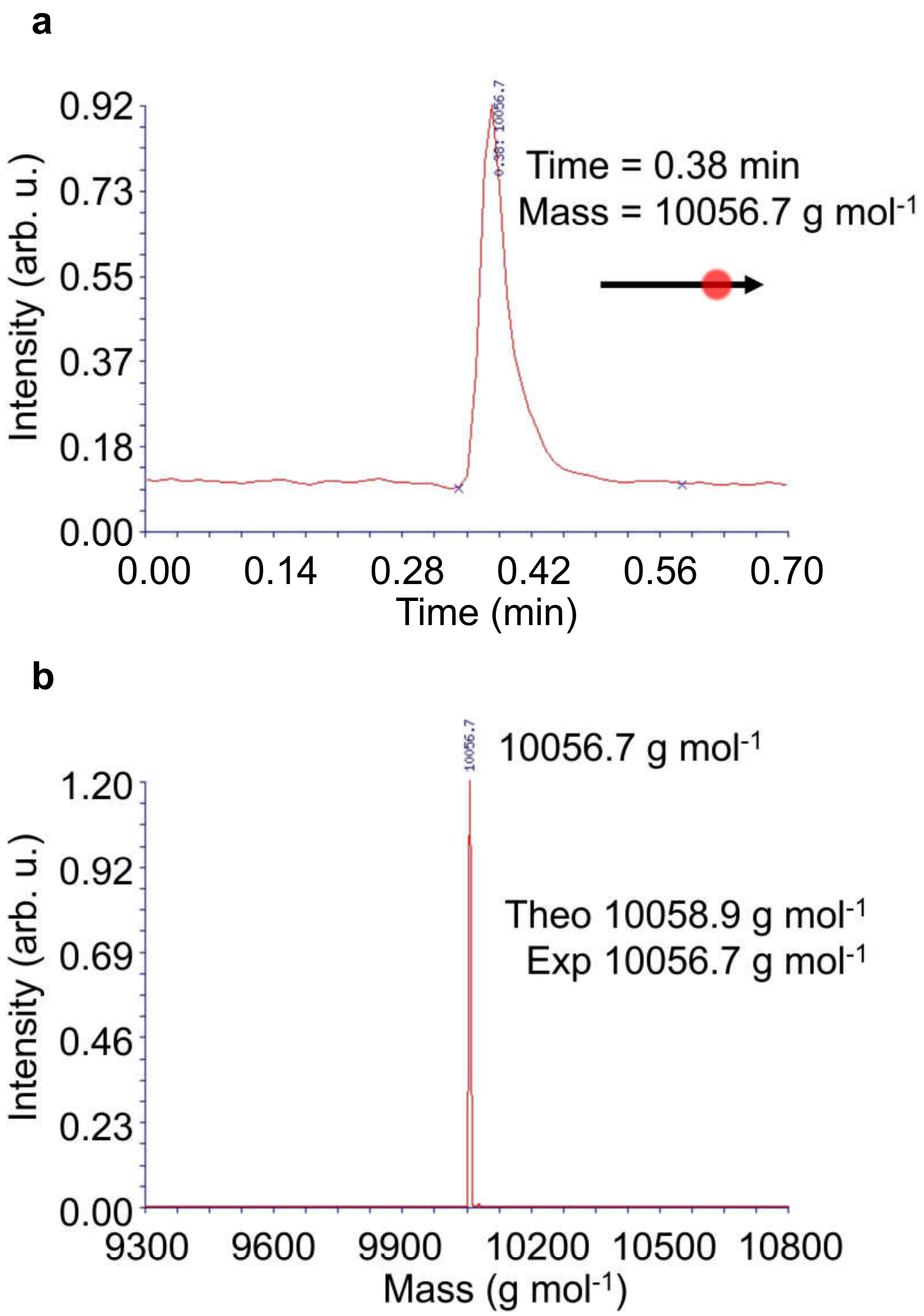

Supplementary Fig. 8. LCMS chromatogram (a) and ESI mass spectrum (b) of cBDP (*S*)-DNA (7nt). The theoretical values (Theo) and experimental mass (Exp) are labelled, confirming the success synthesis and purity of cBDP (*S*)-DNA (7nt). The cBDP is represented by a red spot and the DNA backbone is represented by a black arrow.

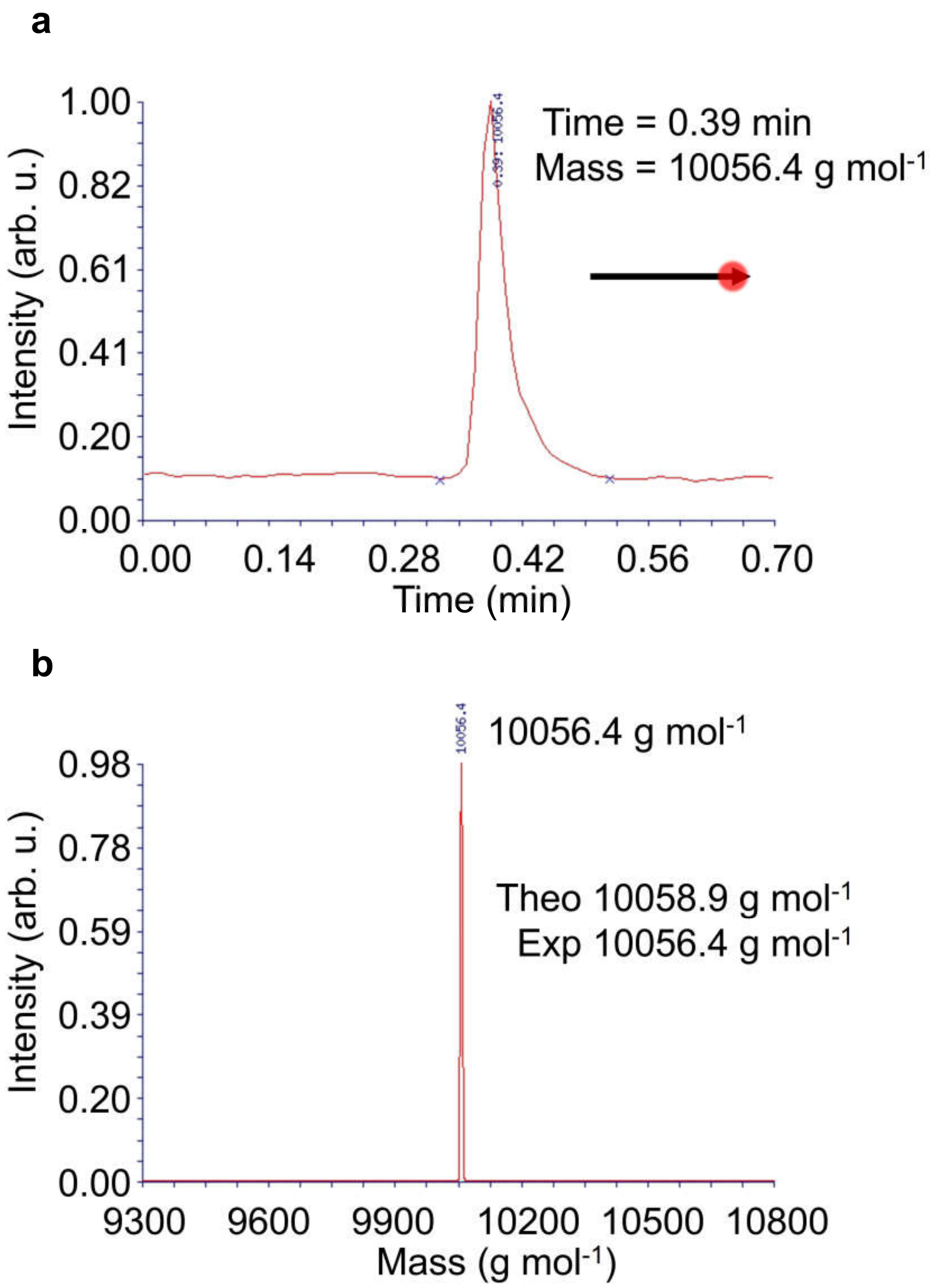

Supplementary Fig. 9. LCMS chromatogram (a) and ESI mass spectrum (b) of cBDP (*S*)-DNA (2nt). The theoretical values (Theo) and experimental mass (Exp) are labelled, confirming the success synthesis and purity of cBDP (*S*)-DNA (2nt). The cBDP is represented by a red spot and the DNA backbone is represented by a black arrow.

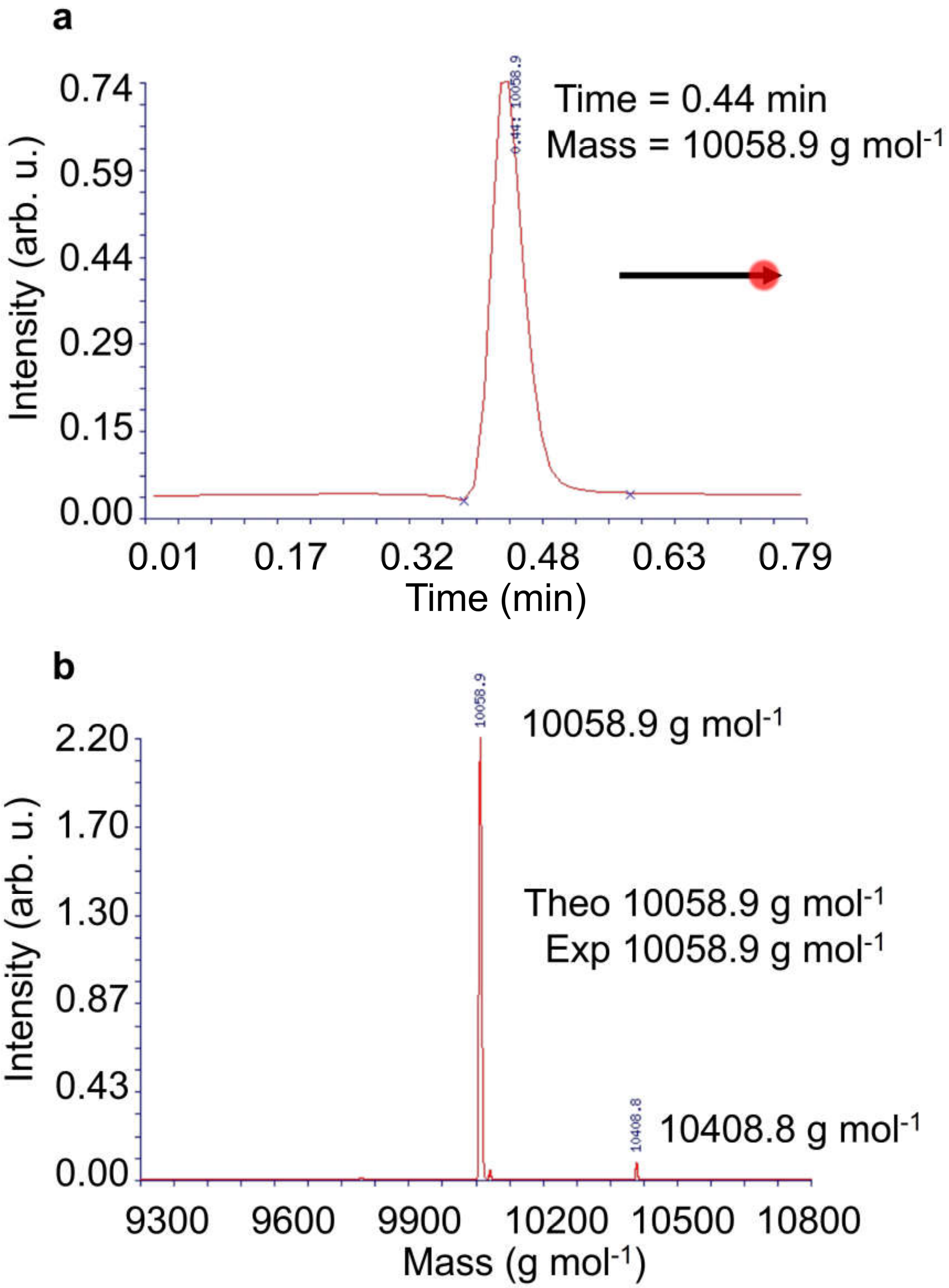

Supplementary Fig. 10. LCMS chromatogram (a) and ESI mass spectrum (b) of cBDP (*R*)-DNA (2nt). The theoretical values (Theo) and experimental mass (Exp) are labelled, confirming the success synthesis and purity of cBDP (*R*)-DNA (2nt). The cBDP is represented by a red spot and the DNA backbone is represented by a black arrow.

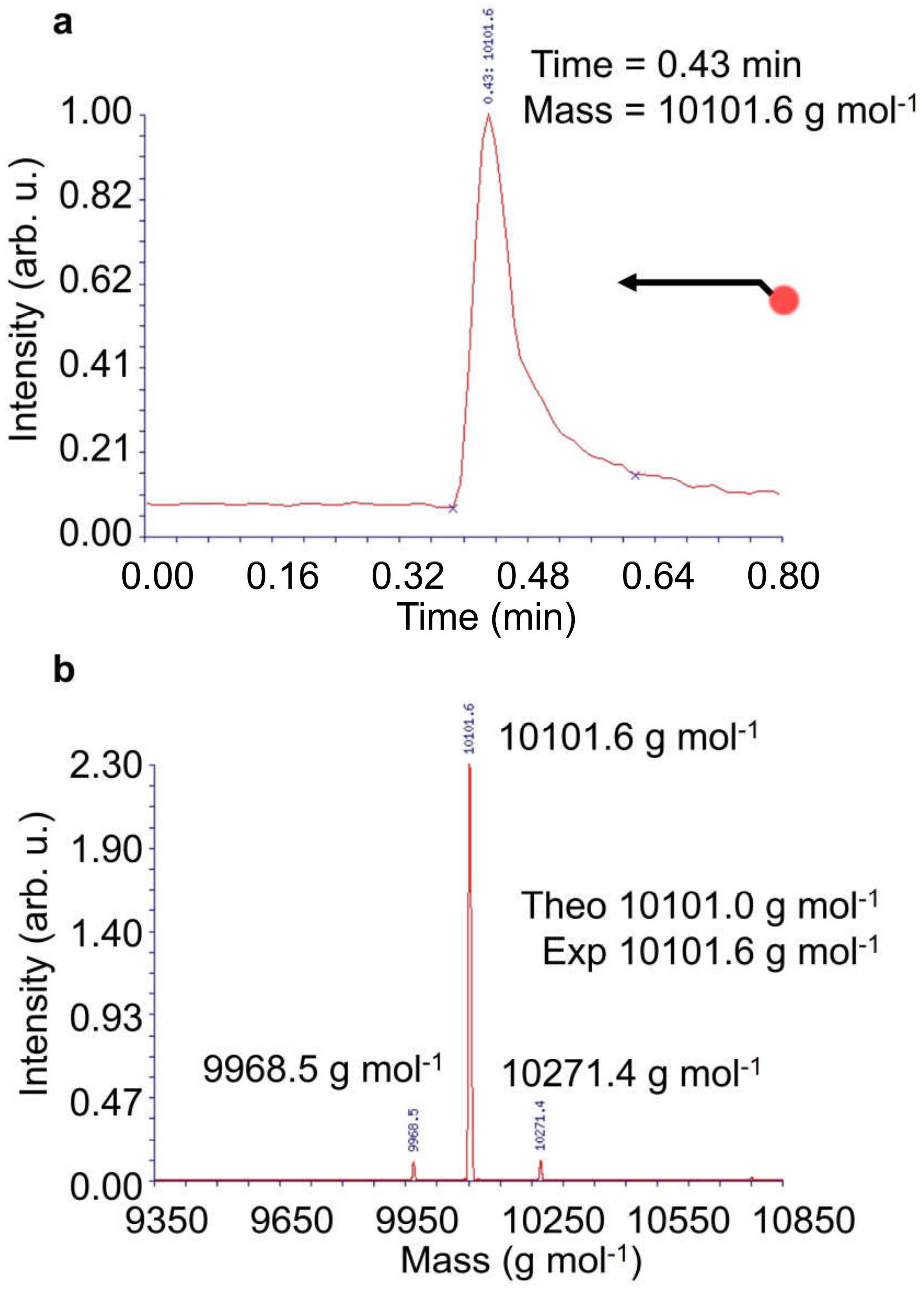

Supplementary Fig. 11. LCMS chromatogram (a) and ESI mass spectrum (b) of DNA strand 4. The theoretical values (Theo) and experimental mass (Exp) are labelled, confirming the success synthesis and purity of DNA strand 4. The cBDP is represented by a red spot and the DNA backbone is represented by a black arrow.

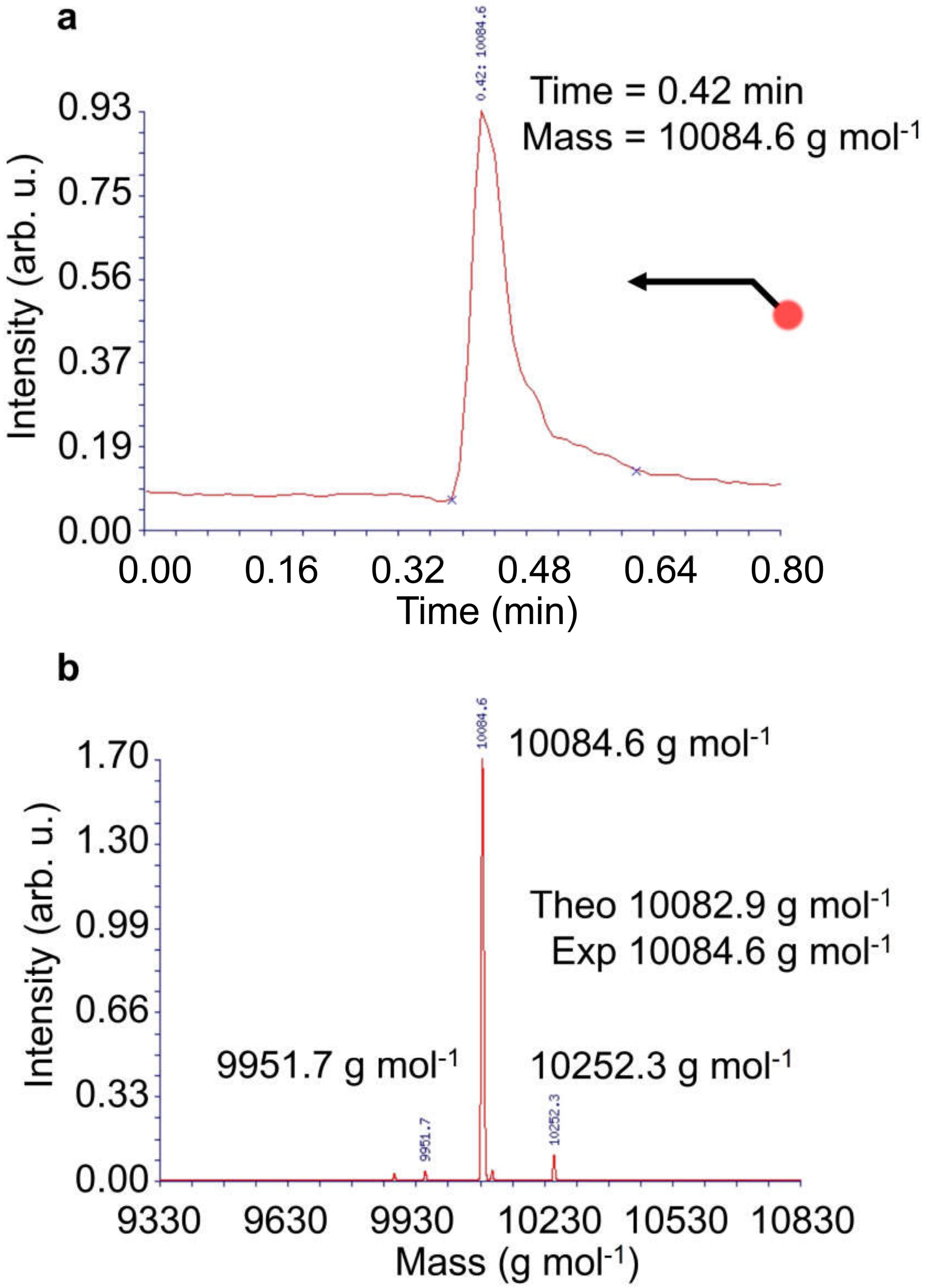

Supplementary Fig. 12. LCMS chromatogram (a) and ESI mass spectrum (b) of DNA strand 5. The theoretical values (Theo) and experimental mass (Exp) are labelled, confirming the success synthesis and purity of DNA strand 5. The cBDP is represented by a red spot and the DNA backbone is represented by a black arrow.

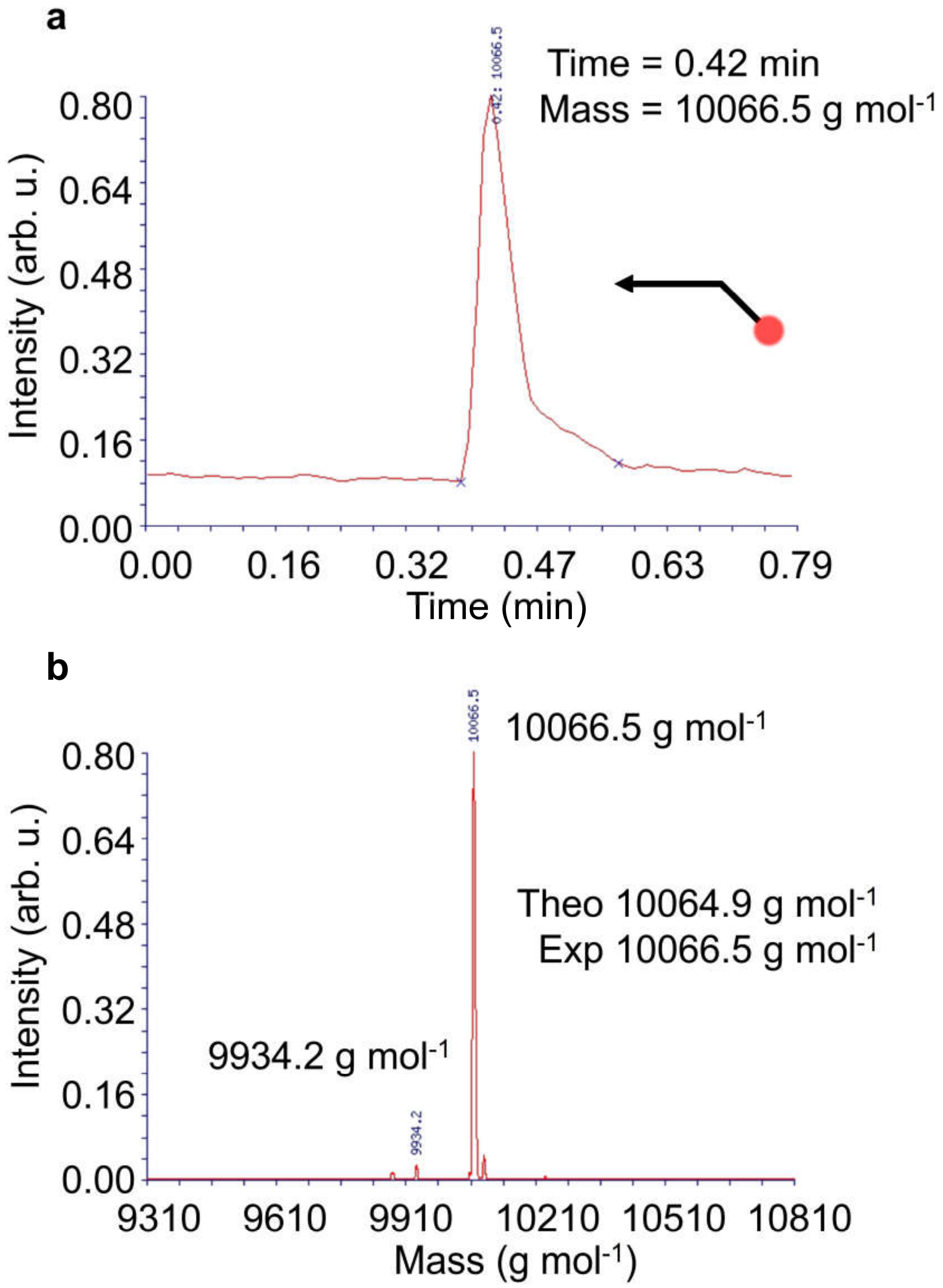

Supplementary Fig. 13. LCMS chromatogram (a) and ESI mass spectrum (b) of DNA strand 6. The theoretical values (Theo) and experimental mass (Exp) are labelled, confirming the success synthesis and purity of DNA strand 6. The cBDP is represented by a red spot and the DNA backbone is represented by a black arrow.

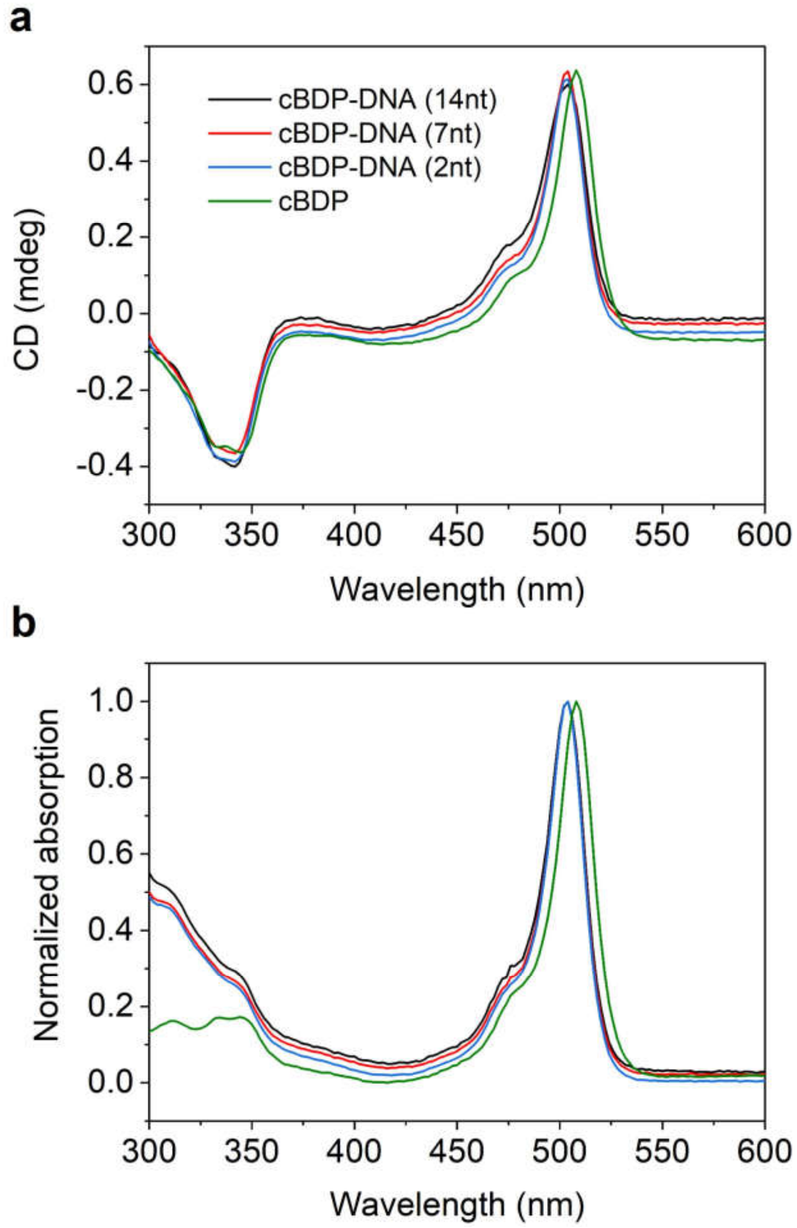

Supplementary Fig. 14. CD (a) and absorption (b) spectra of cBDP-DNA conjugates (cBDP (*S*)-DNA were used in the following) (in TE buffer) and cBDP (in CH_2Cl_2). The cBDP-DNA conjugates exhibited almost the same CD and absorption spectral lineshape as those of free cBDP. The slight redshift of cBDP in CD and absorption spectra compared to cBDP-DNA may be caused by the polarity of the solvent, and similar spectra of cBDP analogue in organic solvents have been reported.[1] Source data are provided as a Source Data file.

Supplementary Note 1. Förster resonance energy transfer (FRET) and site-specificity

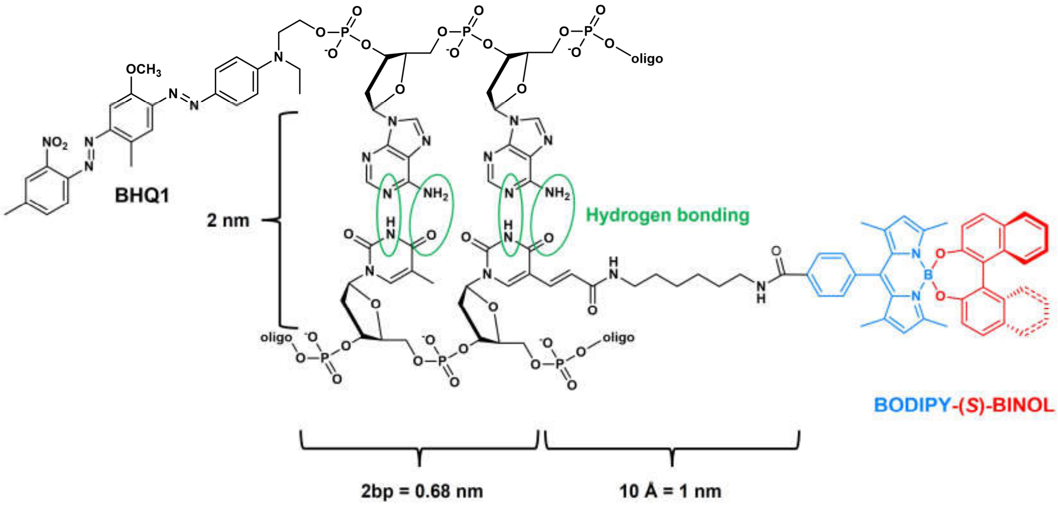

Supplementary Fig. 15. Structural formula of a pair of cBDP and BHQ1 in DNA duplex. The electron-withdrawing BODIPY core is highlighted in blue, and the electron-donating (*S*)-BINOL moiety is highlighted in red. The hydrogen bonding is represented by a green circle.

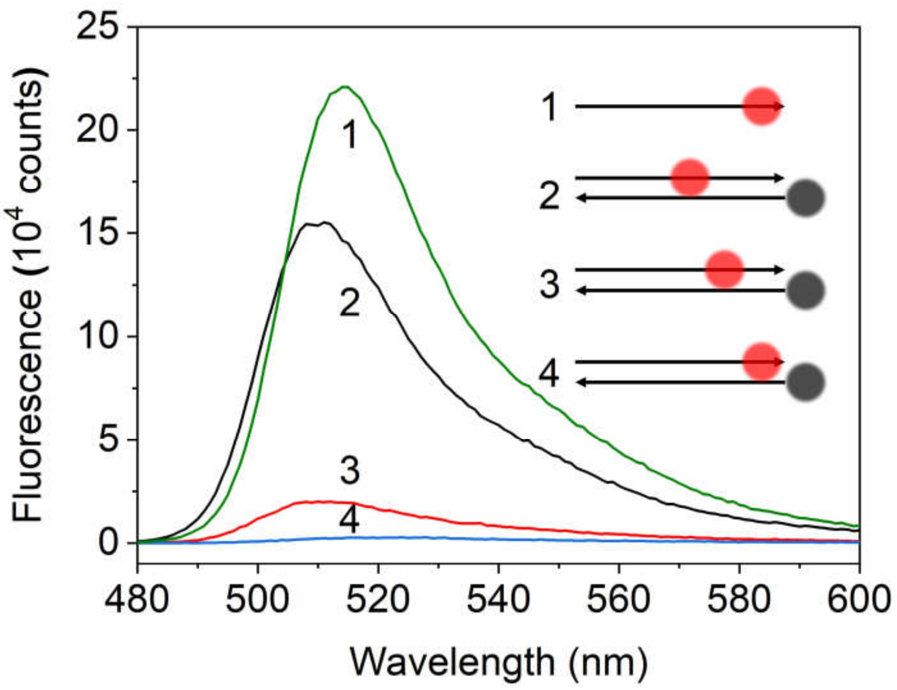

Supplementary Fig. 16. Fluorescence spectra of cBDP-DNA monomer and cBDP-BHQ1 FRET pair with varying inter-distances. The cBDP is represented by a red spot, the BHQ1 is represented by a black spot and the DNA backbone is represented by a black arrow. Source data are provided as a Source Data file.

Supplementary Note 2. Stability test of chromophore-DNA conjugates

Before synthesizing hybrid complexes, we tested the stability of cBDP-DNA conjugates. pH adjustment of buffer and use of tris(2-carboxyethyl)phosphine hydrochloride (TCEP•HCl) were necessary during AuNP surface modifications, and dithiothreitol (DTT) was used to replace the cBDP-DNA conjugates on AuNP surfaces for quantitative analysis. Thus, relevant stability tests of cBDP-DNA conjugates are required.

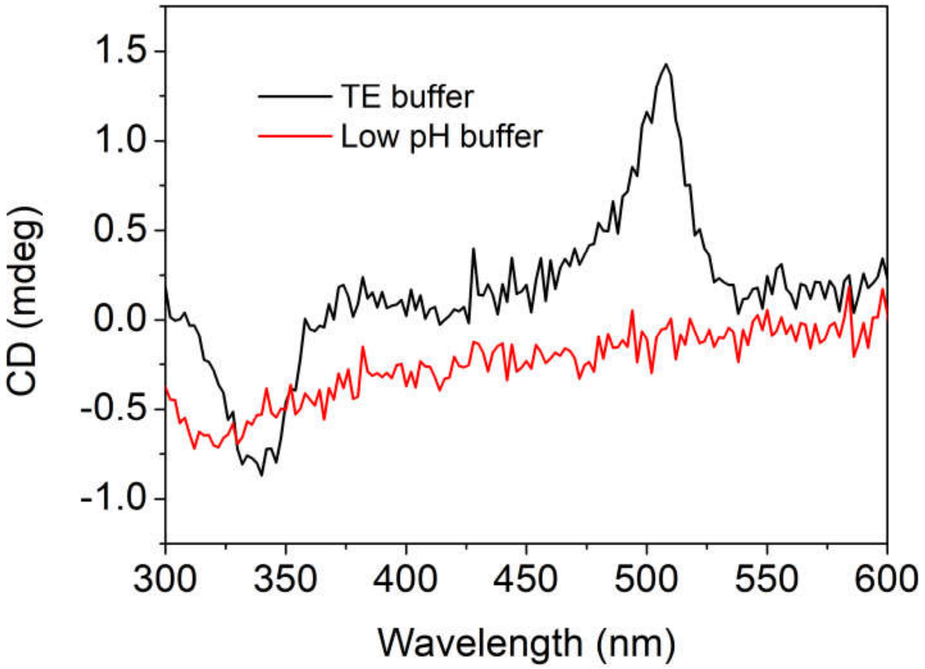

Supplementary Fig. 17. Stability test of cBDP-DNA (2nt) against pH changes. The black and red lines are the same concentration of cBDP-DNA (2nt) in TE buffer (pH = 8) and citrate-HCl buffer (pH = 3) for 30 min, respectively. Source data are provided as a Source Data file.

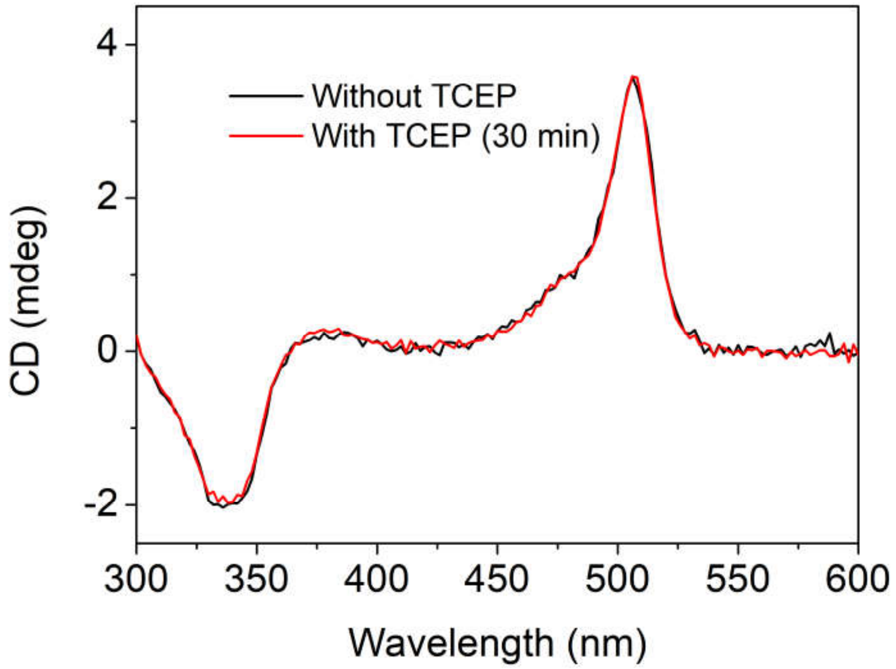

Supplementary Fig. 18. TCEP stability test of cBDP-DNA (2nt). The black and red lines are the same concentration cBDP-DNA (2nt) without and with TCEP for 30 min, respectively. Source data are provided as a Source Data file.

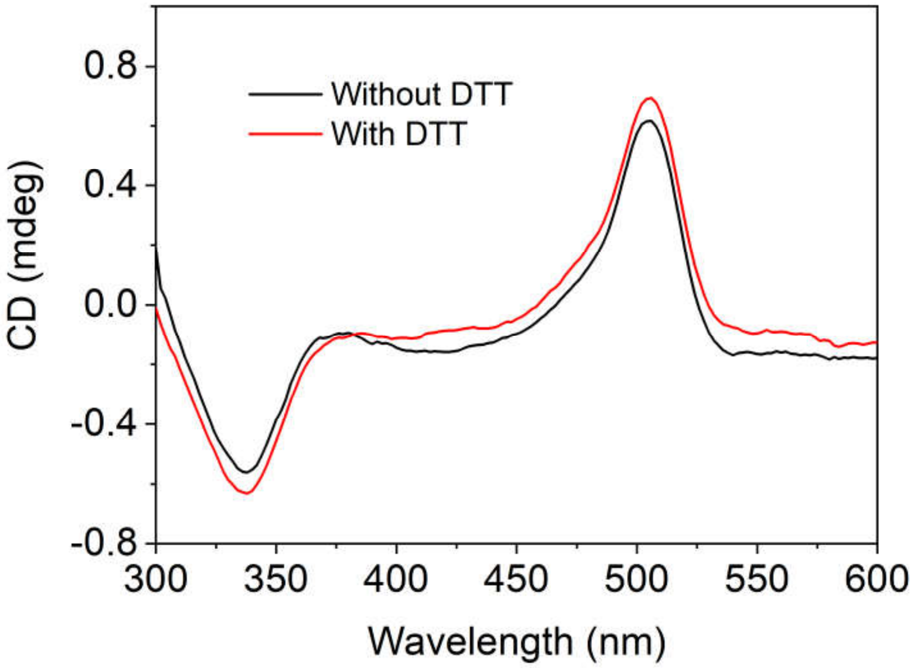

Supplementary Fig. 19. DTT stability test of cBDP-DNA (2nt). The black and red lines are the same concentration cBDP-DNA (2nt) without and with DTT (2.5 μL, 1M) overnight, respectively. Source data are provided as a Source Data file.

Supplementary Note 3. Aggregation test of chromophore-DNA conjugates

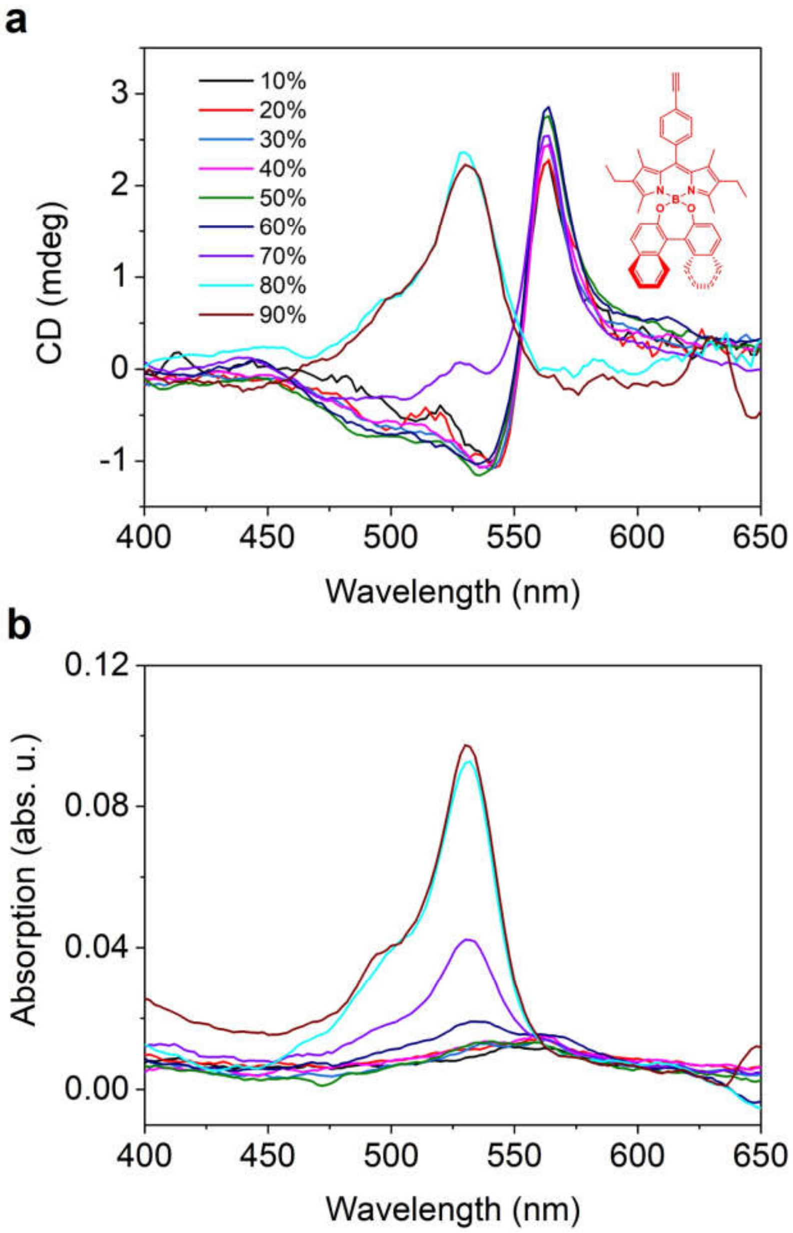

Supplementary Fig. 20. (a) CD spectra of a derivative of BODIPY-(*S*)-BINOL (denoted as alkynyl-cBDP) and (b) the corresponding absorption spectra with various volume-percentage of DMSO in DMSO-water mixed solvents. At low volume-percentage of DMSO, the CD spectra exhibited a bisignated splitting lineshape,[2] indicating that alkynyl-cBDP tends to aggregate in close proximity, promoting intermolecular excitonic correlations. As the volume-percentage of DMSO increased, the CD spectra evolved into a single positive band that is blue shifted. This band resembled the CD lineshape of cBDP-DNA monomer, as depicted in Supplementary Fig. 14a. Source data are provided as a Source Data file.

a

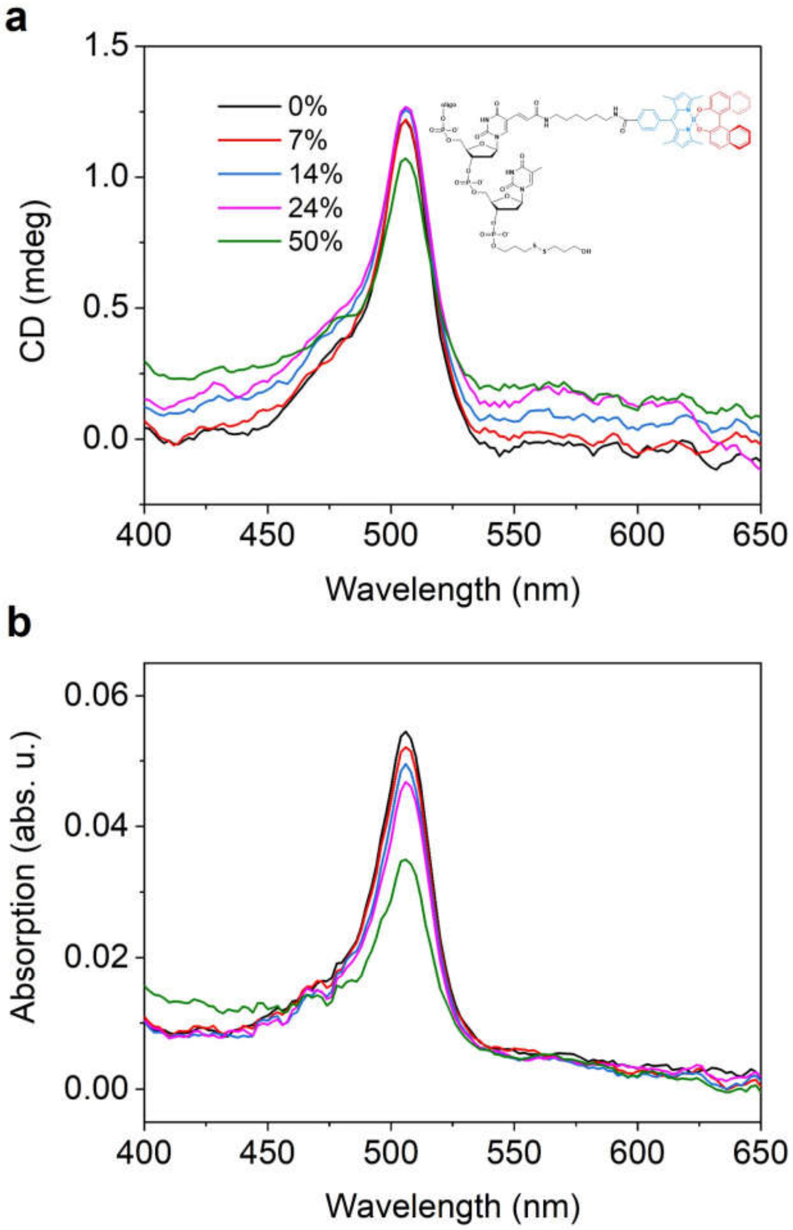

Supplementary Fig. 21. (a) CD spectra of cBDP-DNA (2nt) and (b) the corresponding absorption spectra with various volume-percentage of DMSO in DMSO-water mixed solvents. cBDP-DNA (2nt) has good solubility in water. The addition of DMSO is for comparison with Supplementary Fig. 20. With the addition of DMSO, there is no spectral shift or bisignated splitting observed in the CD spectra. These results mean that almost no aggregation of cBDP-DNA conjugates occurred in aqueous solution under the experimental conditions. The electron-withdrawing BODIPY core is highlighted in blue, and the electron-donating (*S*)-BINOL moiety is highlighted in red. Source data are provided as a Source Data file.

Supplementary Note 4. Quantification of surface concentration of chromophore-DNA conjugates

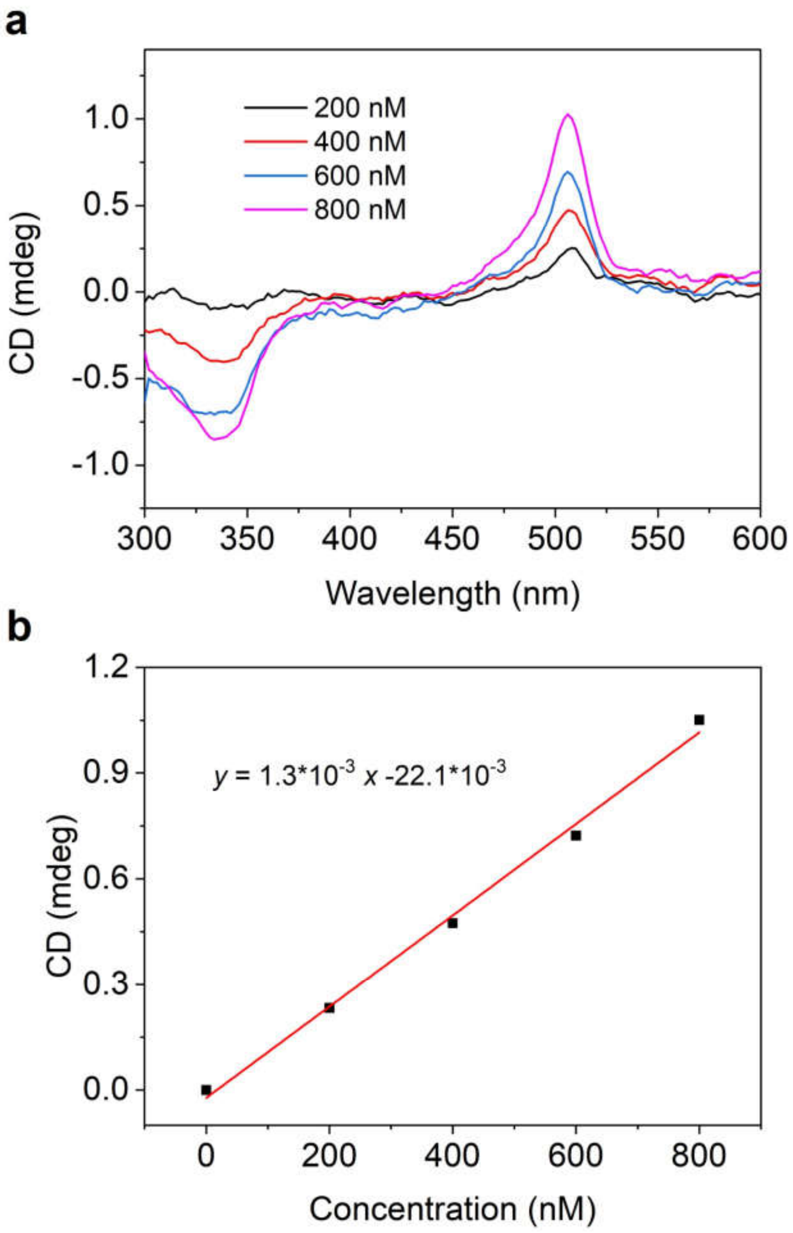

Supplementary Fig. 22. CD spectra of cBDP-DNA (2nt) with various concentrations (a) and standard curve via linear fitting (R^2 = 0.9933, intercept = -0.0221, slope = 0.0013) (b). The linear relationship ranges from 0 to 800 nM, if sample concentration exceeds 800 nM, it can be diluted to the linear region for further calculation. Source data are provided as a Source Data file.

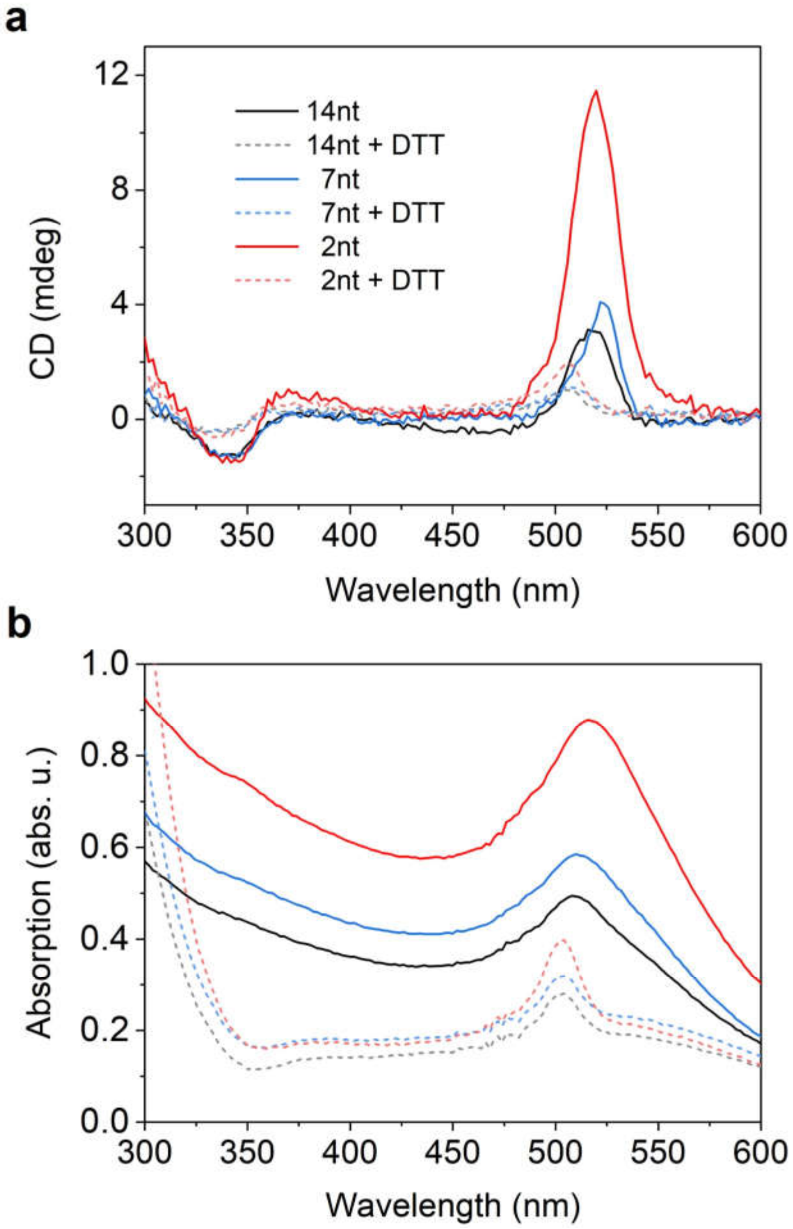

Supplementary Fig. 23. CD spectra of 5 nm Au complexes before (solid lines) and after (dashed lines) DTT addition (a) and their corresponding absorption spectra (b). The blue shift and intensity decrease of the dashed lines in the CD and absorption spectra are related to the substitution of cBDP-DNA conjugates on the surface of AuNPs by DTT. It is obvious that after DTT addition, the cBDP-DNA conjugates were released into the solution. Source data are provided as a Source Data file.

Supplementary Note 5. Additional experimental data

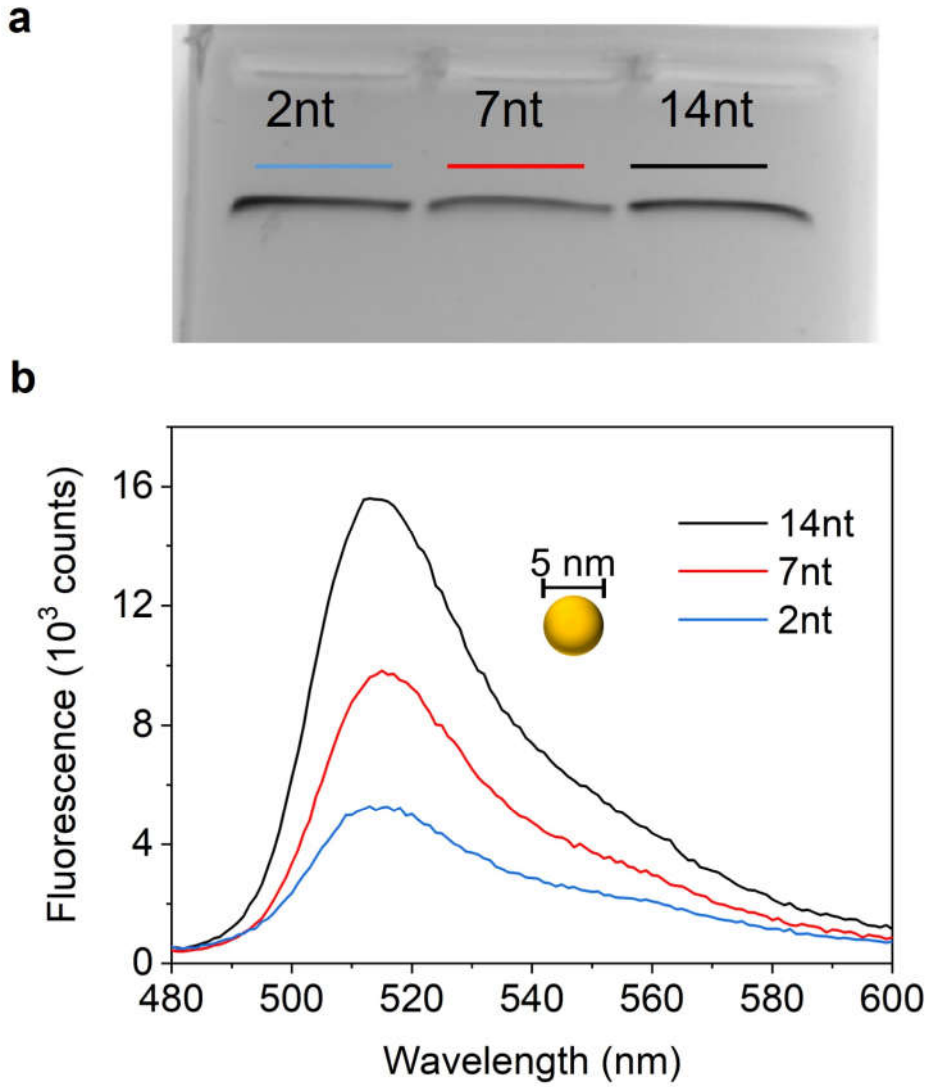

Supplementary Fig. 24. Agarose gel image of three complexes (a) and their fluorescence spectra (b). Three complexes showed single narrow bands of similar mobility, consistent with the similar cBDP-DNA surface concentrations in Supplementary Table 1. As compared with the fluorescence intensity of cBDP-DNA monomer in Supplementary Fig. 16, the fluorescence of three complexes was largely quenched by AuNPs, and the quenching in 2nt complexes was higher than in 7nt and 14nt complexes. Source data are provided as a Source Data file.

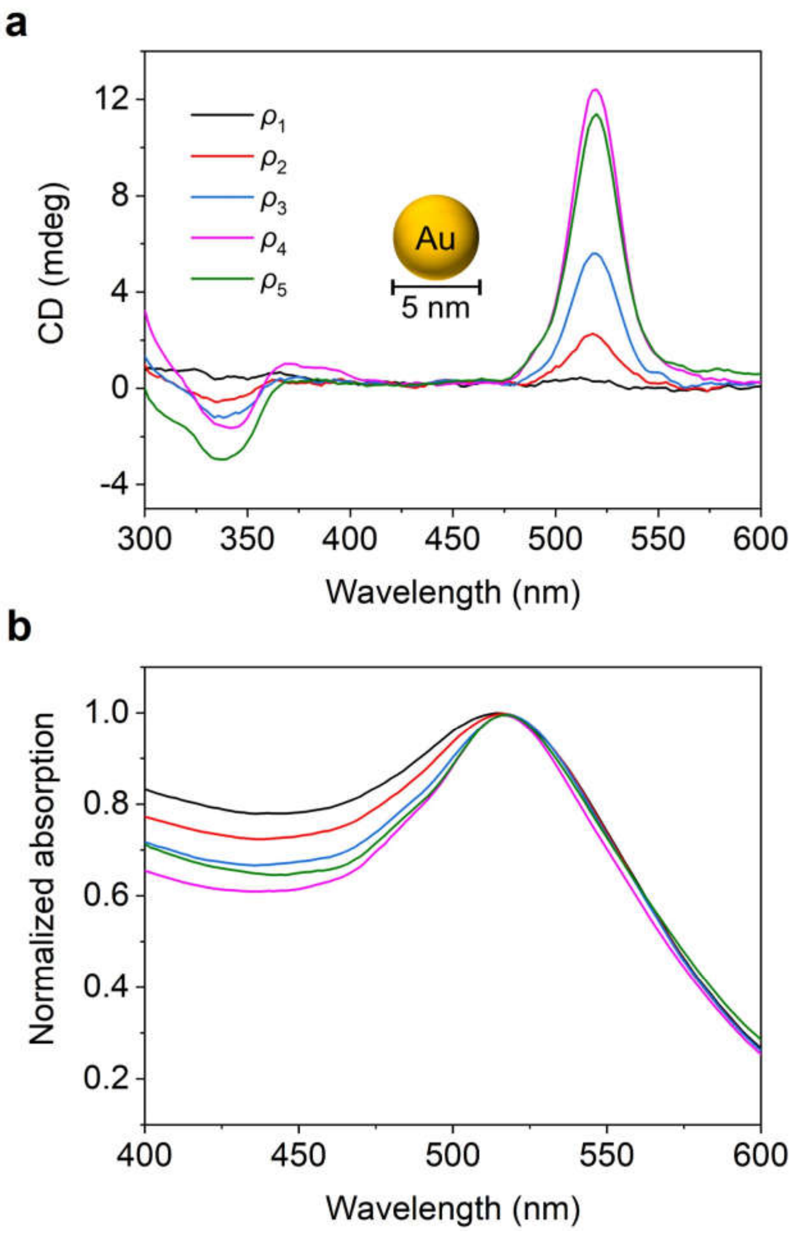

Supplementary Fig. 25. CD (a) and absorption (b) spectra of Au complexes (5 nm, 2nt) with varying surface concentrations (*ρ*) of cBDP. The changing trends of enhancement factor and internal ratio in Au complexes (5 nm, 2nt) with varying surface concentrations were presented in the main text. Supplementary Fig. 25a provides a visual representation of the slight decrease of $|CD_{520\ nm}|$ from ρ_4 to ρ_5. This can be attributed to the potential adverse effects of cBDP overcrowding on molecular stacking, thereby impeding the enhancement of CD. Source data are provided as a Source Data file.

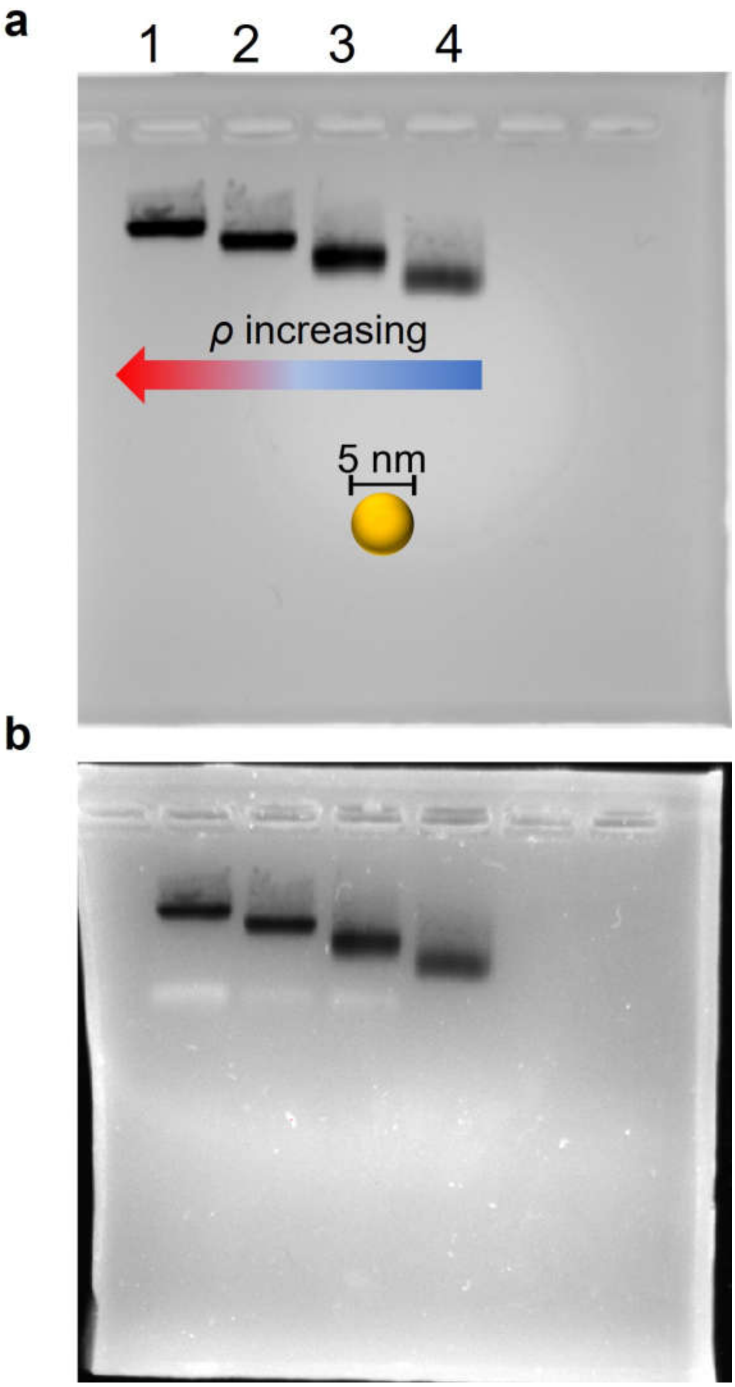

Supplementary Fig. 26. Agarose gel image of Au complexes (5 nm, 2nt) with varying surface concentrations (*ρ*) of cBDP under white (a) and UV light (b). The preparation procedure is as follows: 800 µL of 5 nm AuNPs solution was mixed with various volumes of cBDP-DNA (2nt) (25 µM), then frozen at -20 °C overnight. Lane 1: ρ_3 (40 µL), lane 2: ρ_2 (20 µL), lane 3: ρ_1 (10 µL), lane 4: ρ_0 (4 µL), corresponding to Supplementary Table 2 and Supplementary Fig. 25. Source data are provided as a Source Data file.

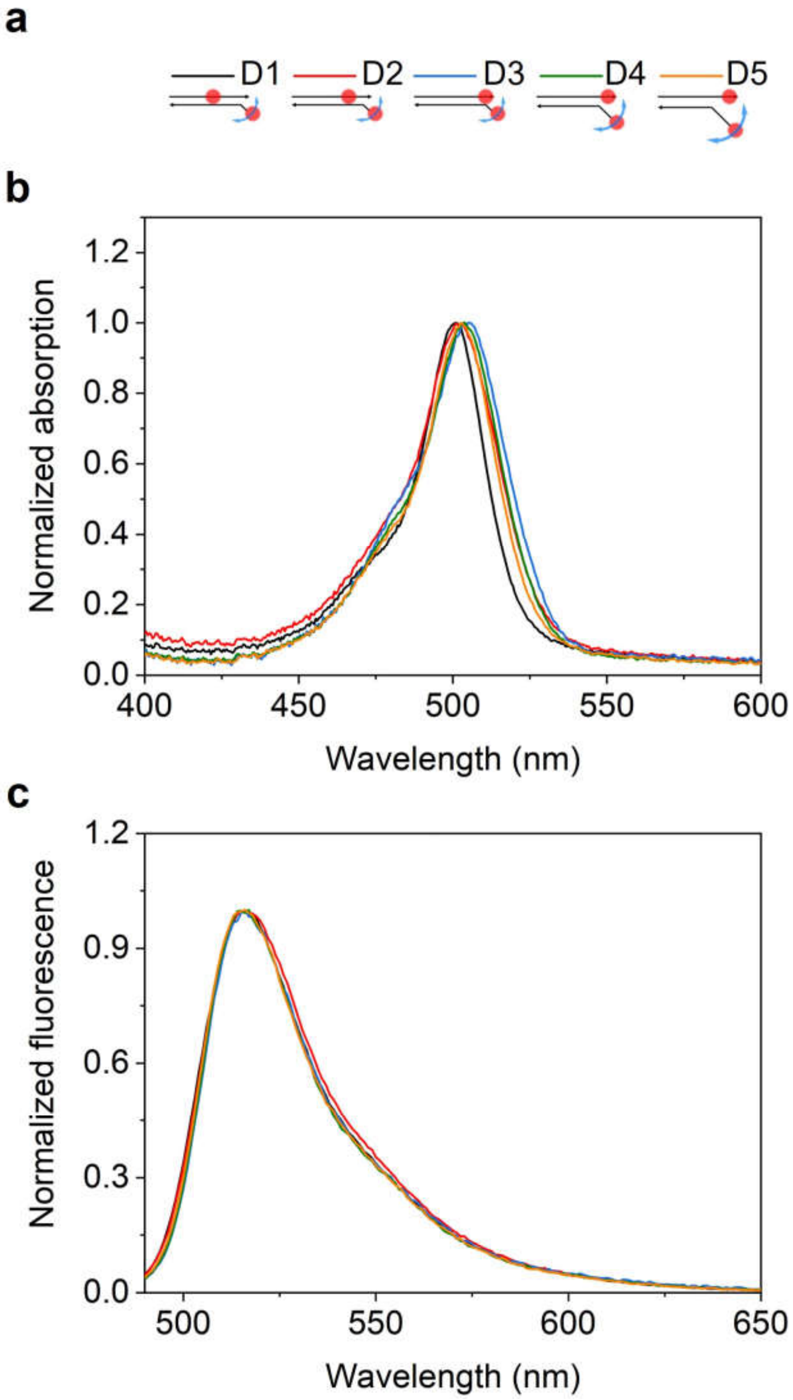

Supplementary Fig. 27. Schematic (a), absorption (b) and fluorescence spectra (c) (excitation: 480 nm) of cBDP dimers. D1: DNA strands 1 and 4, D2: DNA strands 2 and 4, D3: DNA strands 3 and 4, D4: DNA strands 3 and 5, D5: DNA strands 3 and 6, as depicted in main text Fig. 3a. The sequence of DNA strands 1-6 can be found in Supplementary Table 6. The fluorescence spectra are almost identical for all dimers, possibly due to null exciton coupling mediated monomer-like optical characteristics.[3] Source data are provided as a Source Data file.

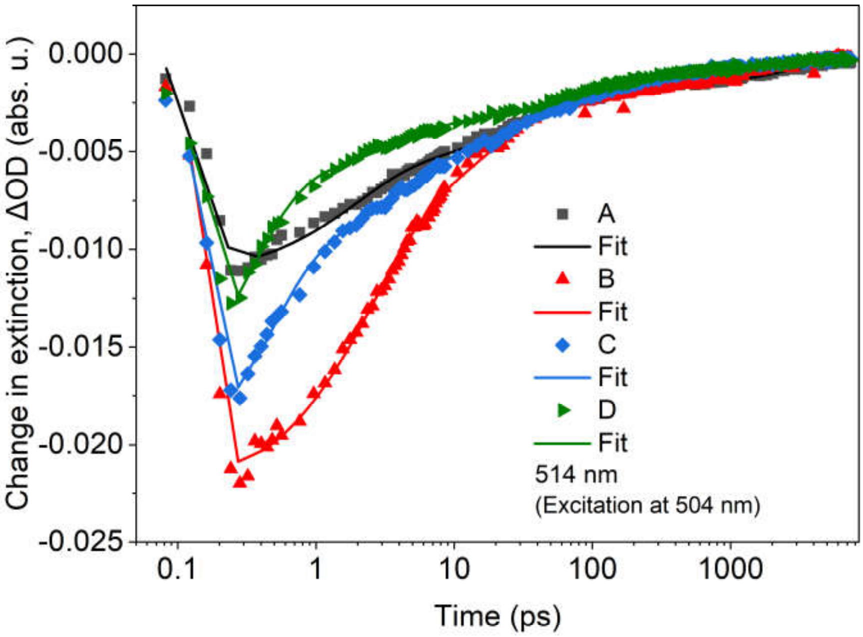

Supplementary Fig. 28. Transient kinetics for cBDP-DNA monomer (A) and D1-D3 (B-D) at the same timescale (probed at 514 nm). The fits are fitted curves to the triple (A and B) and quadruple (C and D) exponential decay with the instrument response function. Source data are provided as a Source Data file.

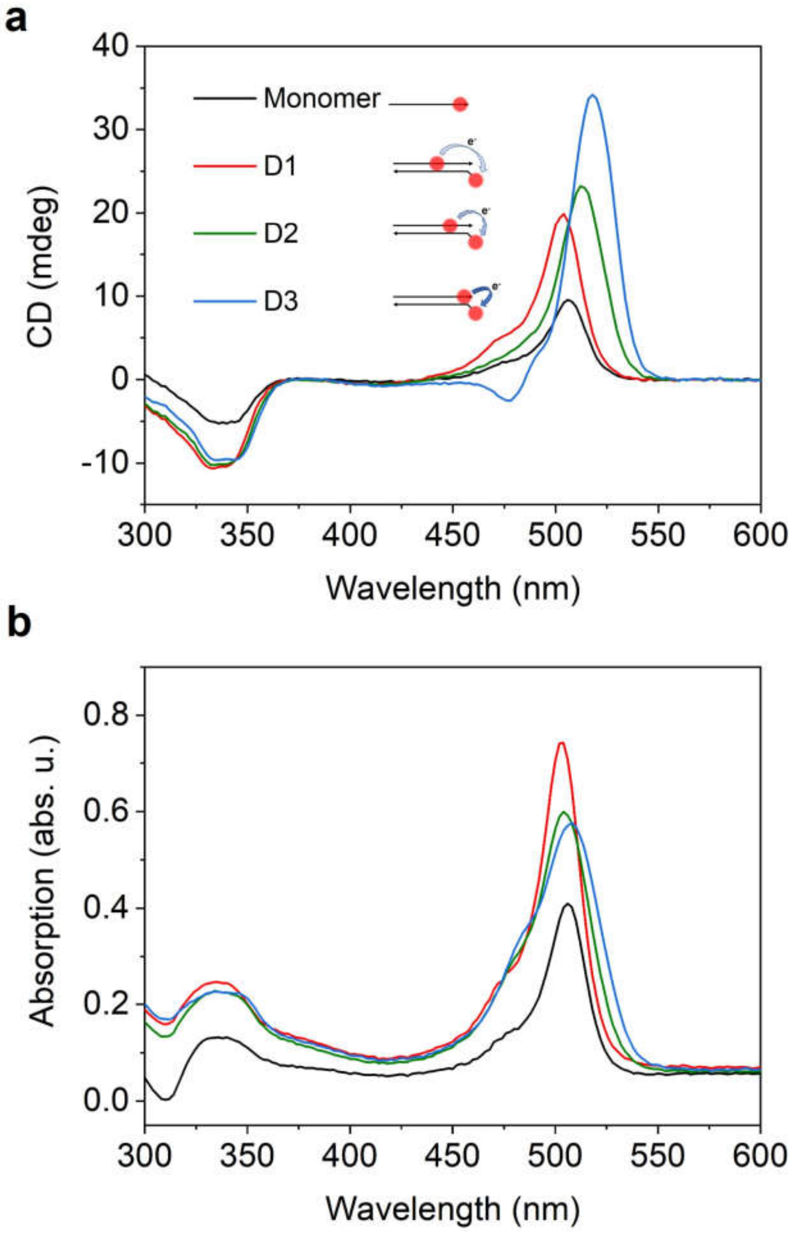

Supplementary Fig. 29. CD (a) and absorption (b) spectra of cBDP-DNA monomer and D1-D3 before TA experiments. D1: DNA strands 1 and 4, D2: DNA strands 2 and 4, D3: DNA strands 3 and 4, as depicted in main text Fig. 3a. Source data are provided as a Source Data file.

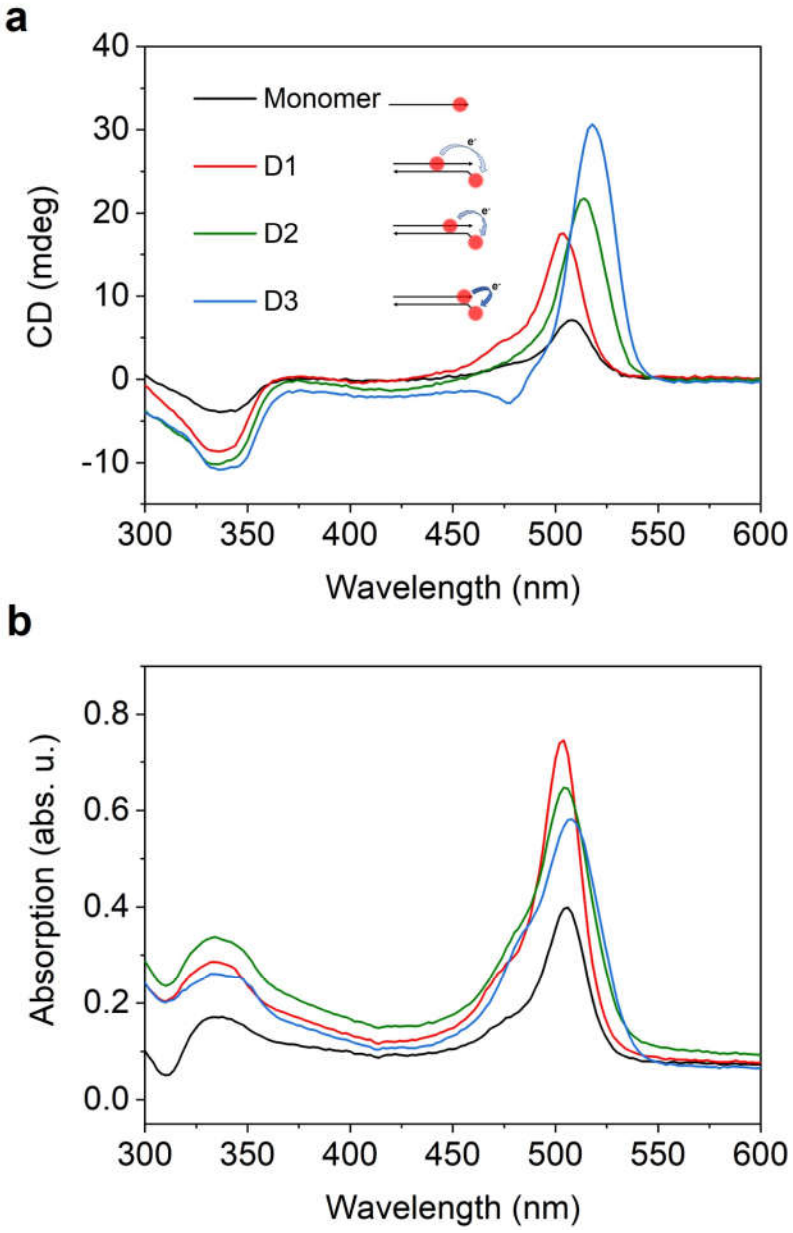

Supplementary Fig. 30. CD (a) and absorption (b) spectra of cBDP-DNA monomer and D1-D3 after TA experiments. D1: DNA strands 1 and 4, D2: DNA strands 2 and 4, D3: DNA strands 3 and 4, as depicted in main text Fig. 3a. Supplementary Figs. 29-30 showed that photodegradation during TA experiments was negligible. Source data are provided as a Source Data file.

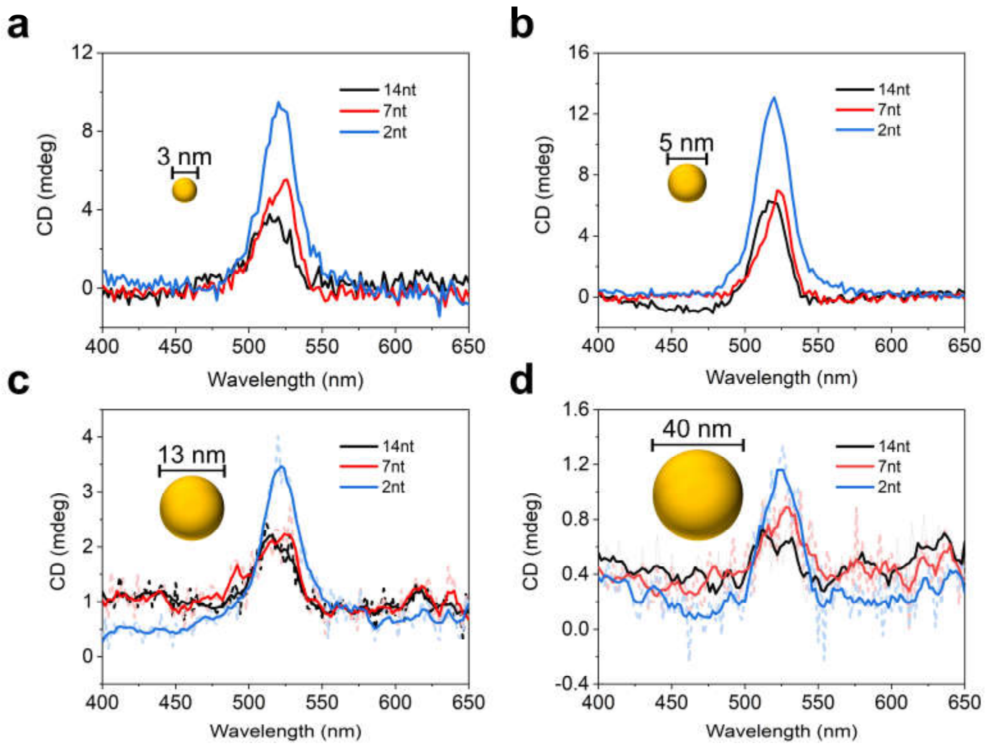

Supplementary Fig. 31. CD spectra of single-NP complexes with size variation. The CD intensity of all 2nt complexes is the highest, with little difference between 7nt and 14nt complexes for each AuNP size. Asymmetric CD lineshape can be observed in both 7nt complexes of 3 nm and 5 nm AuNP sizes. The data in main text Fig. 4b are experimental results (appropriate spectral smooth fitting for 13 nm and 40 nm complexes). Source data are provided as a Source Data file.

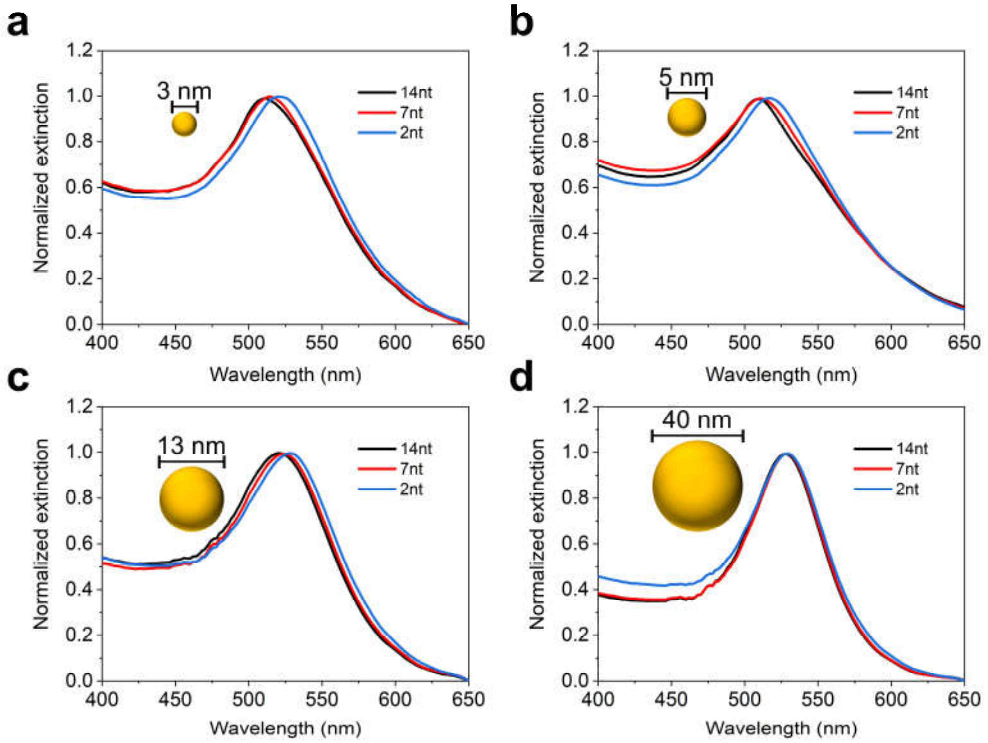

Supplementary Fig. 32. Absorption spectra of single-NP complexes with size variation. All complexes showed a redshift of maximum absorption wavelength because of both the dielectric effect and intermolecular couplings as cBDP-Au gap decreased from 14nt to 7nt and 2nt. Source data are provided as a Source Data file.

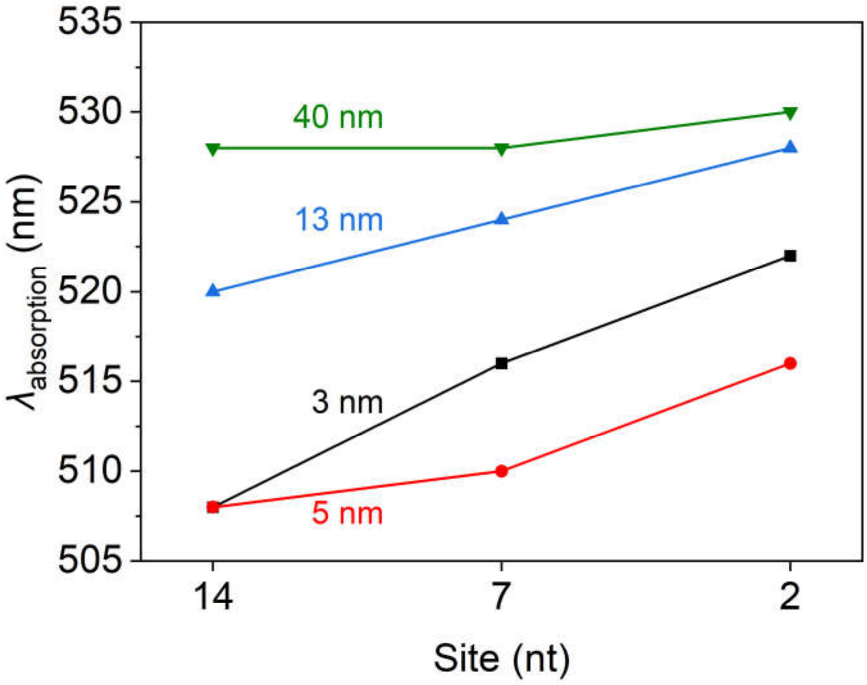

Supplementary Fig. 33. Maximum absorption wavelength of single-NP complexes with size variation. Source data are provided as a Source Data file.

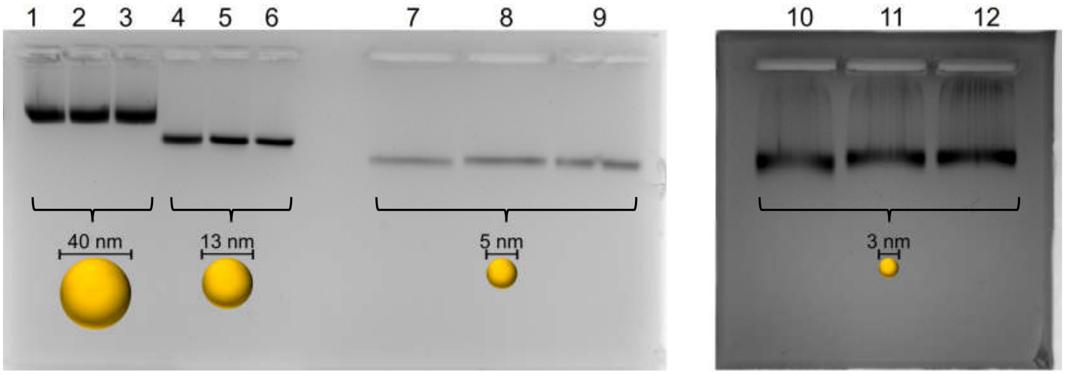

Supplementary Fig. 34. Agarose gel image of 40 nm, 13 nm, 5 nm and 3 nm Au complexes. Typically, for 5 nm complexes, the preparation procedure is as follows: 800 μL of 5 nm AuNPs solution was mixed with equal amount of cBDP-DNA (14nt, 7nt and 2nt respectively), then frozen at -20 °C overnight. Lane 1: 40 nm 14nt, lane 2: 40 nm 7nt, lane 3: 40 nm 2nt, lane 4: 13 nm 14nt, lane 5: 13 nm 7nt, lane 6: 13 nm 2nt, lane 7: 5 nm 14nt, lane 8: 5 nm 7nt, lane 9: 5 nm 2nt, lane 10: 3 nm 14nt, lane 11: 3 nm 7nt, lane 12: 3 nm 2nt. All complexes showed single narrow bands and the complexes (14nt, 7nt and 2nt) of the same size have similar mobility, consistent with their similar cBDP-DNA surface concentrations. Source data are provided as a Source Data file.

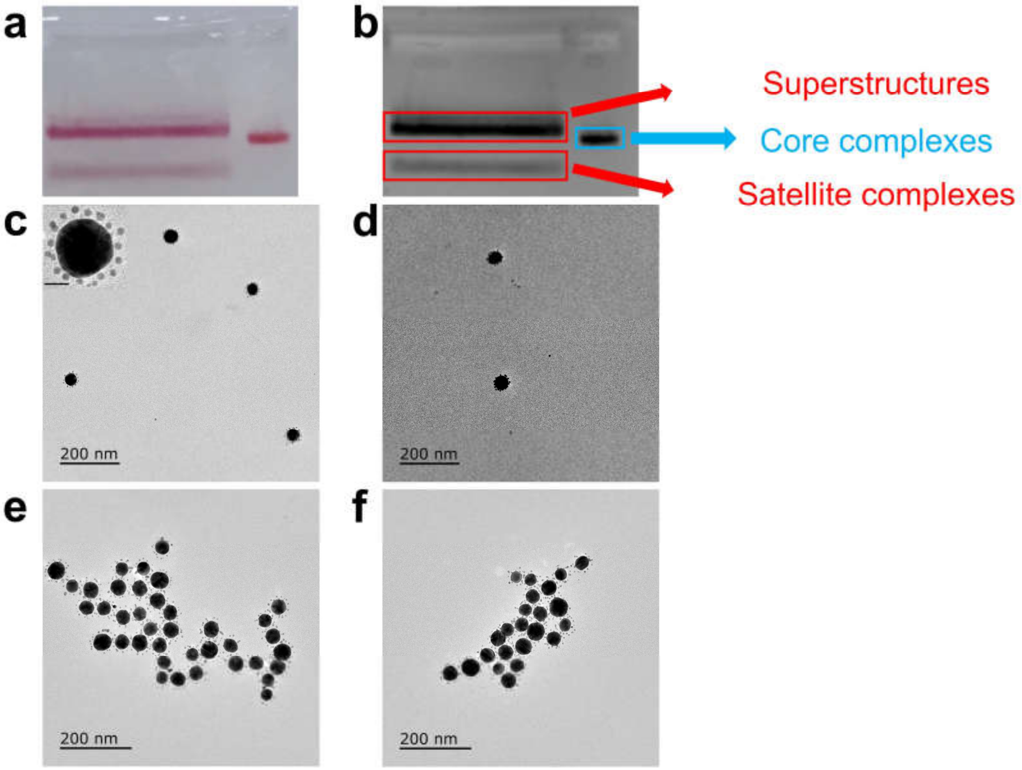

Supplementary Fig. 35. Agarose gel (a and b) and TEM images (c-f) of superstructures. Inset in (c) is a zoom-in image of a single superstructure, scale bar is 20 nm. The single narrow band of target products in agarose gel demonstrated mono-dispersity of superstructures. Source data are provided as a Source Data file.

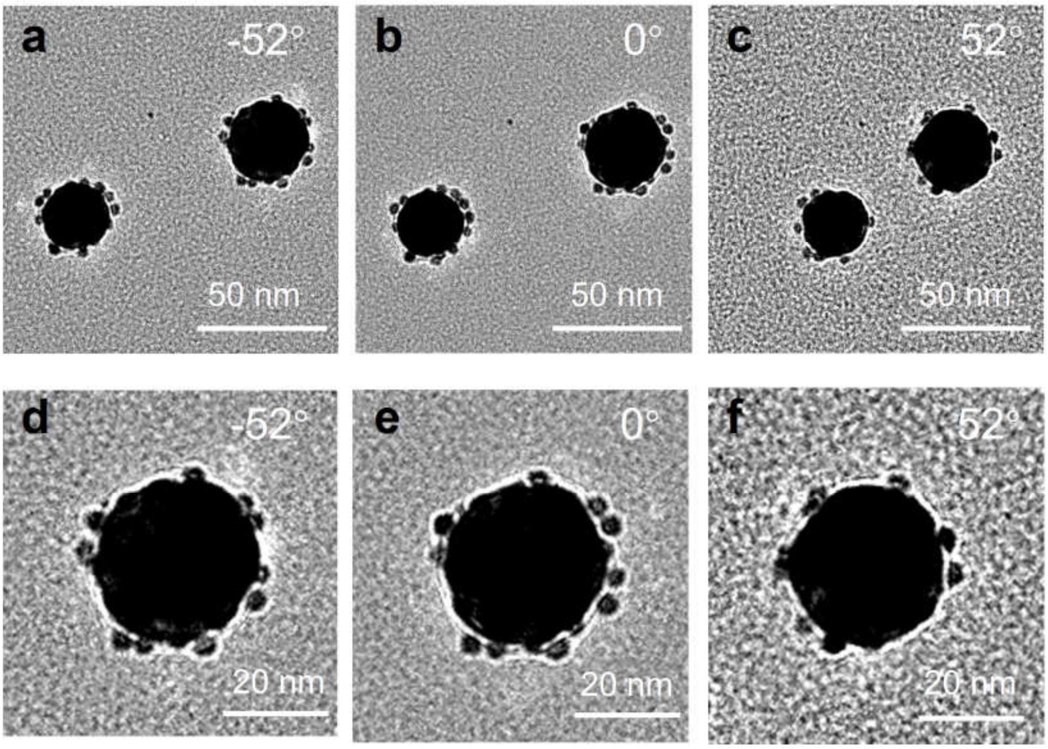

Supplementary Fig. 36. Tilt-TEM images of the superstructures (a-f).

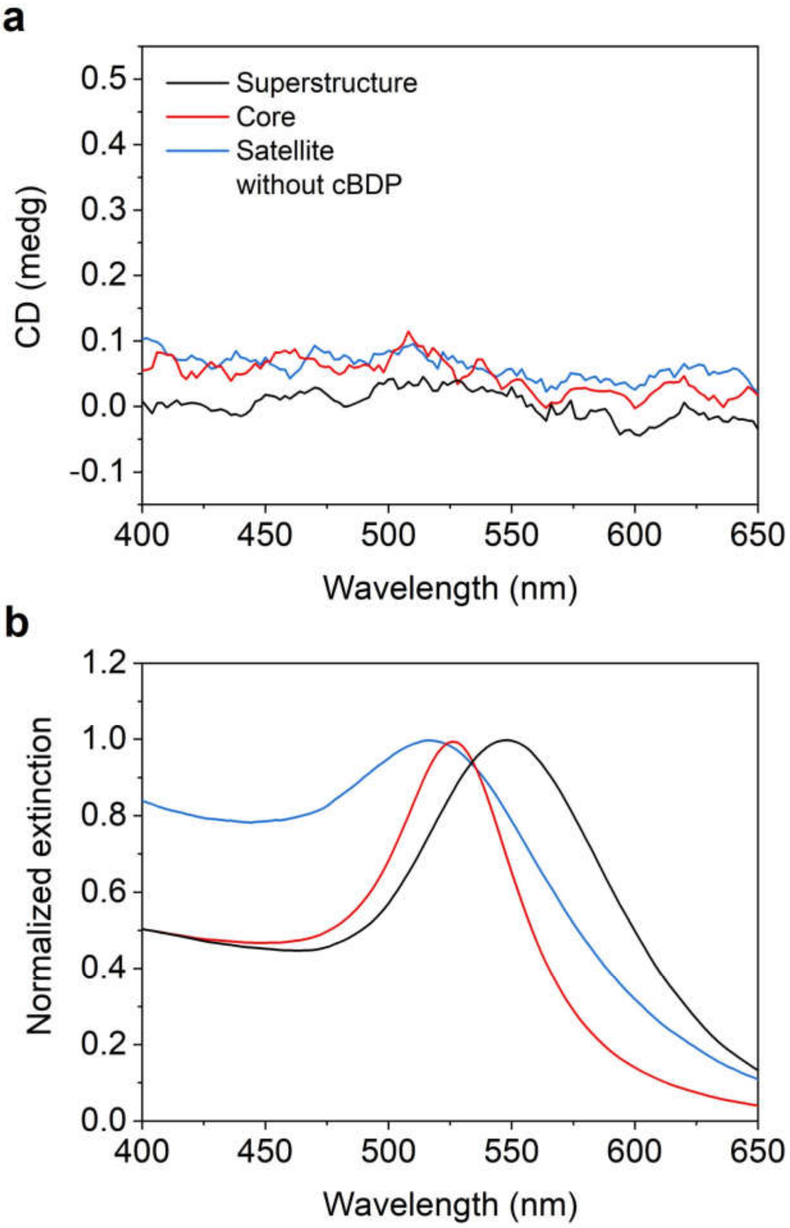

Supplementary Fig. 37. CD (a) and extinction (b) spectra of superstructure without cBDP and the constituent particles, respectively. Source data are provided as a Source Data file.

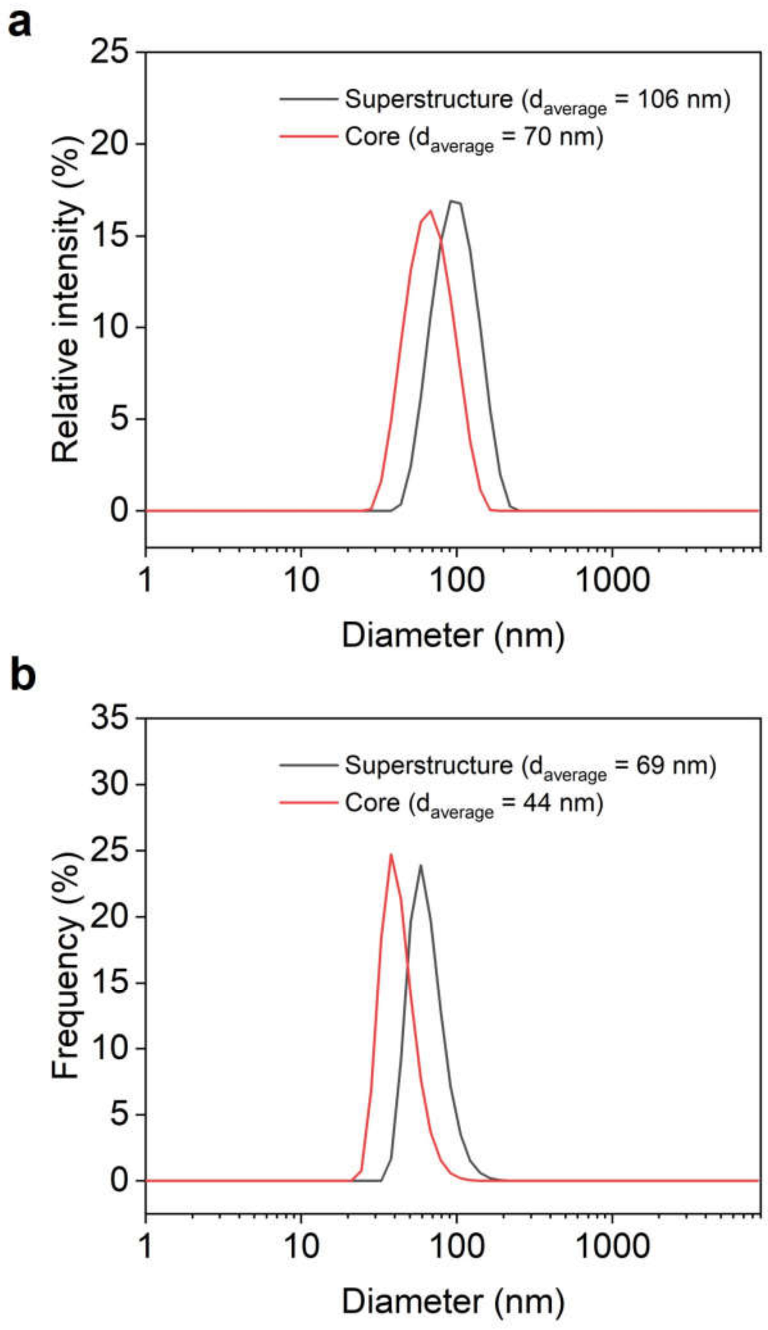

Supplementary Fig. 38. Size distribution of the superstructure and the core particle obtained by dynamic light scattering (DLS). Both samples were directly measured after gel purification. The average size of superstructure less than twice of the core particle size indicated no observable cluster aggregations. Source data are provided as a Source Data file.

Supplementary Note 6. Theoretical analysis

Quantum chemical calculation

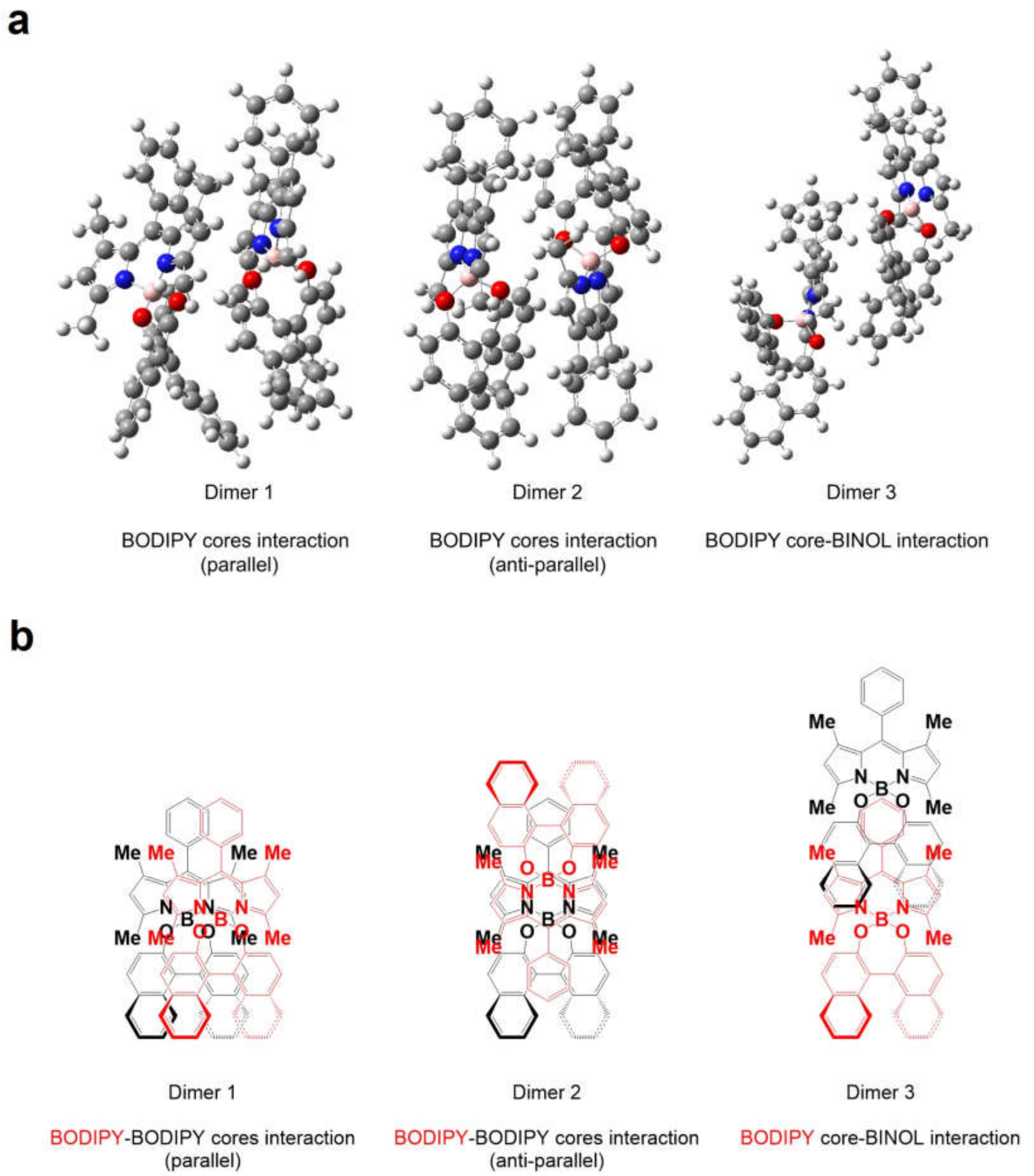

Supplementary Fig. 39. DFT calculated three spatial orientations (a) and their corresponding molecular structural formulae (b) of cBDP dimer. We chose three spatial orientations for comparison, namely, parallel alignment between BODIPY cores, anti-parallel alignment between BODIPY cores, and close alignment between BODIPY core and (*S*)-BINOL moiety.

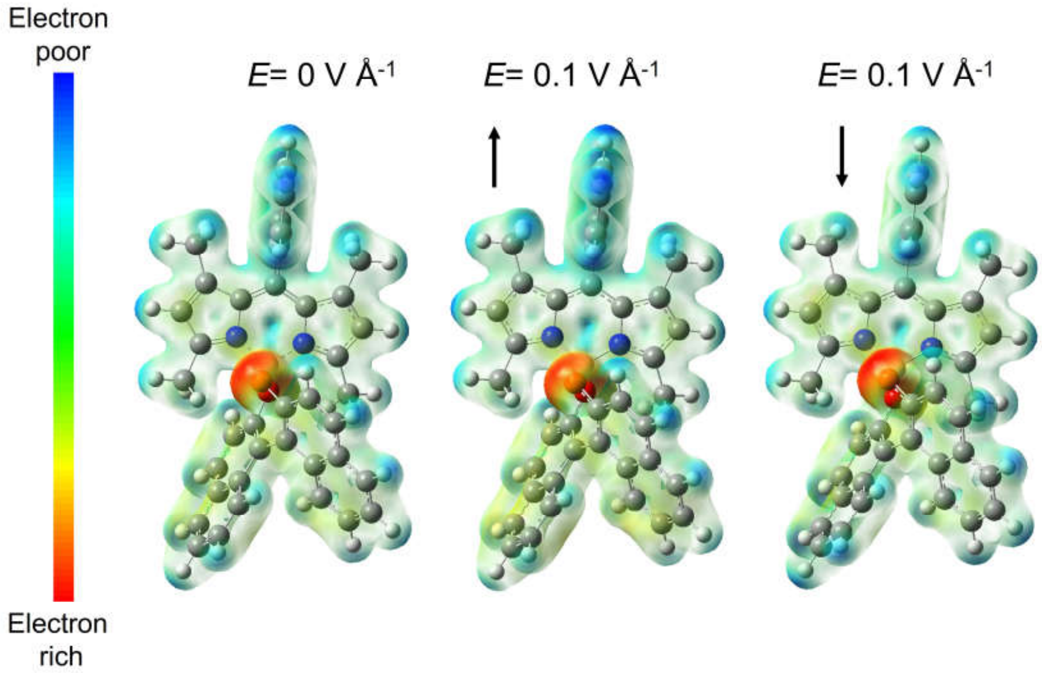

Supplementary Fig. 40. DFT calculated charge density distribution map of cBDP monomer in external electric field. It can be observed that the charge density distribution of BODIPY core changes in external electric field, especially on the adjacent benzene ring and the (*S*)-BINOL moiety.

Electromagnetic simulation

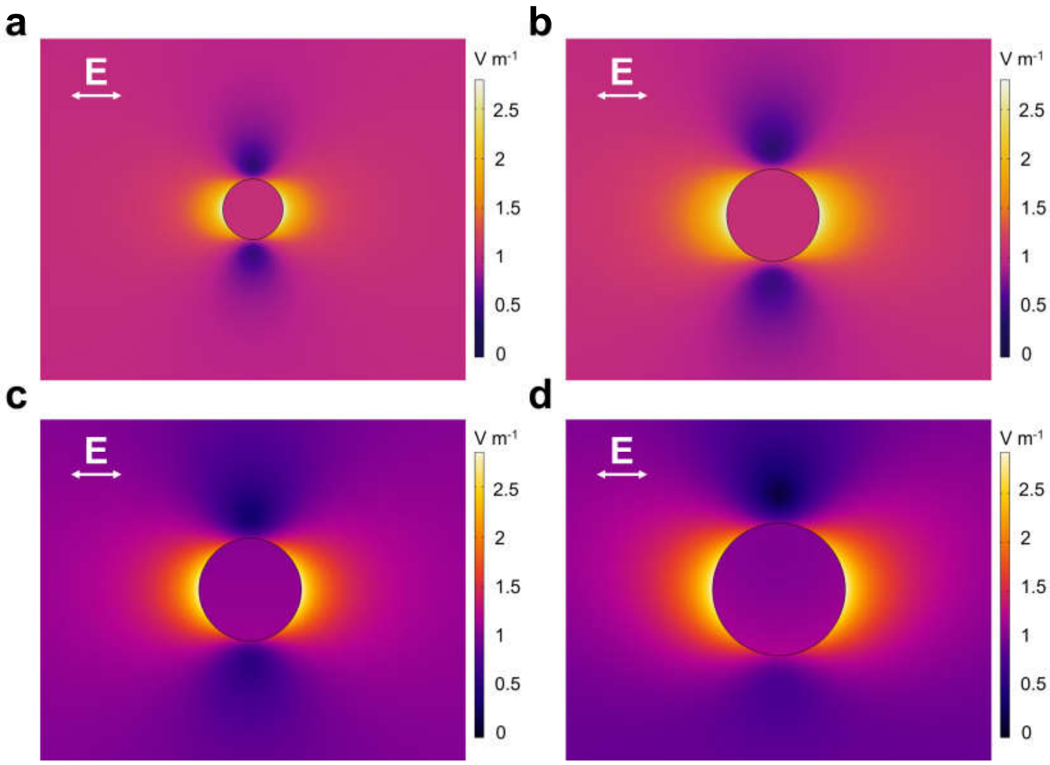

Supplementary Fig. 41. Electric field distribution maps for different sizes of AuNP (a) 3 nm, (b) 5 nm, (c) 13 nm and (d) 40 nm. The smaller the particle size, the more inhomogeneous the distribution of electric field intensity near the AuNP surface, for example, within a surface distance less than 5 nm.

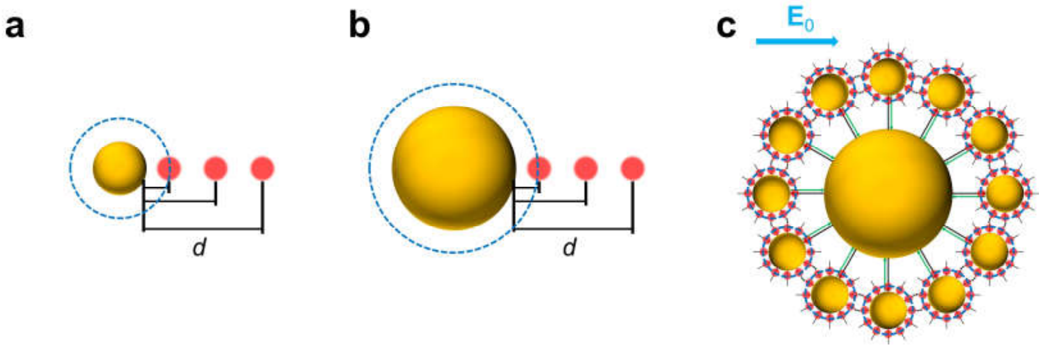

Supplementary Fig. 42. Schematic of integration models of 5 nm and 40 nm AuNP (a-b) and superstructure (c) (*d*=2nt). Note that (**a**) for 5 nm AuNP, integration around a single, isolated AuNP, (**b**) for 40 nm AuNP, integration around a single, isolated AuNP, (**c**) for the superstructure, the integrals are taken around satellites.

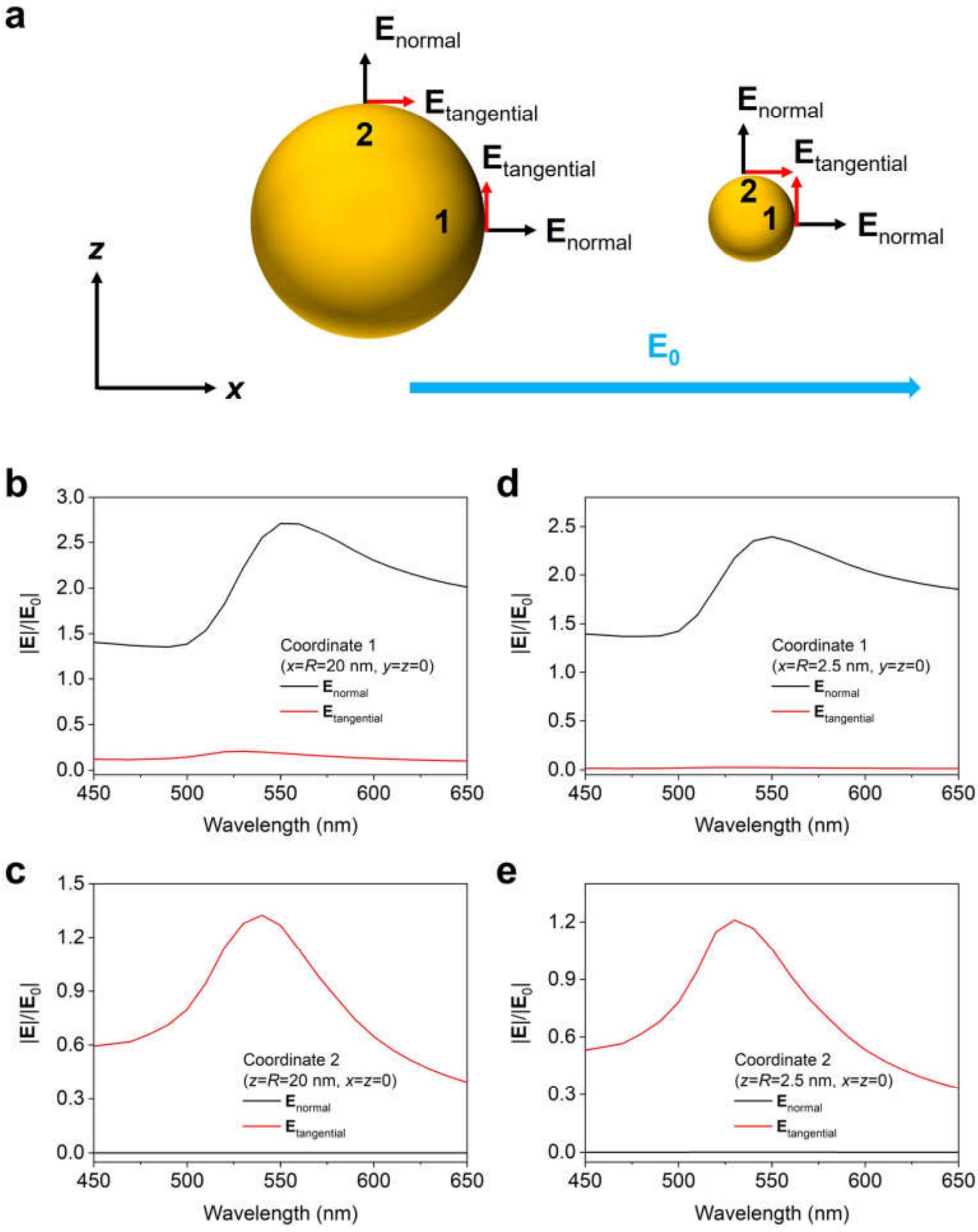

Supplementary Fig. 43. Schematic of 40 nm and 5 nm AuNP models (a) and their calculated |E|/|E$_0$| at coordinates A and B (b-e), respectively. Source data are provided as a Source Data file.

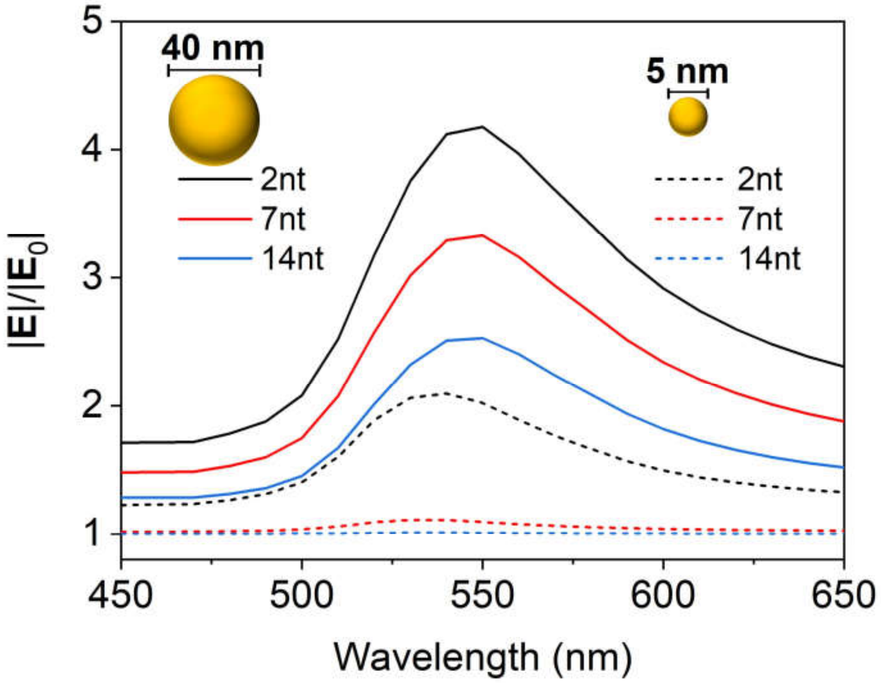

Supplementary Fig. 44. Calculated average |E|/|E$_0$| of 40 nm and 5 nm complexes (14nt, 7nt and 2nt). Source data are provided as a Source Data file.

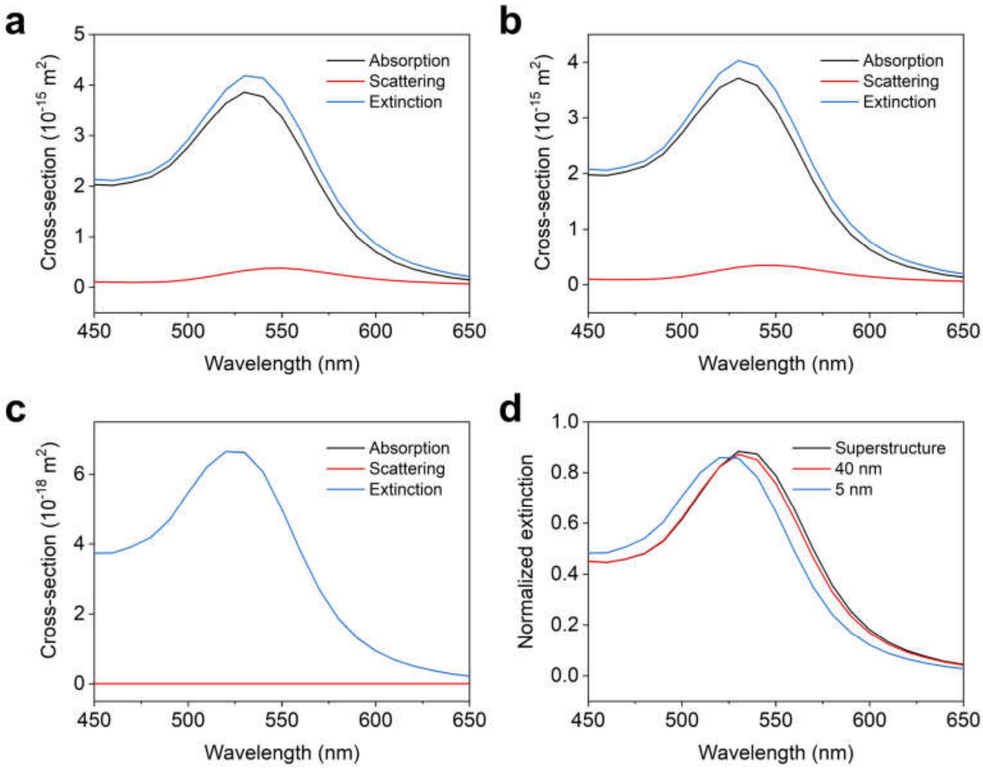

Supplementary Fig. 45. Calculated absorption, scattering and extinction of (a) superstructure, (b) 40 nm AuNP and (c) 5 nm AuNP and (d) their extinction comparison. Source data are provided as a Source Data file.

Supplementary Discussion

We experimentally observed a large extinction redshift of superstructure relative to the individual core particles (Fig. 4g). To study such spectral shift, a 3D model of the superstructure was built theoretically as depicted in Supplementary Fig. 46a. In fact, there are many parameters that need to be considered to meet the experimental conditions. Firstly, we noticed that the dielectric constant of surroundings could be modified as a result of local densification of DNA strands after assembling.[4,5] Also, the number of satellites in the superstructure could be varied between one another. Furthermore, the core-satellite gap distance can be affected by the flexibility of double-stranded DNA linkers. Thus, we systematically studied the influence of change in the dielectric constant of surroundings, the number of satellites and the core-satellite gap distance. As shown in Supplementary Fig. 46b, increasing the dielectric constant of surroundings around the AuNPs causes clear redshift of extinction spectra. More redshift can be generated by increasing the number of satellites (Supplementary Fig. 46c-e). Similarly, reducing the core-satellite gap distance, for example, from 10 nm to 7.5 nm, also leads to an obvious redshift (Supplementary Figs. 46e and 47b). Therefore, we concluded that the observed extinction redshift of superstructure was attributed to the core-satellite plasmonic couplings but affected by structural variations.

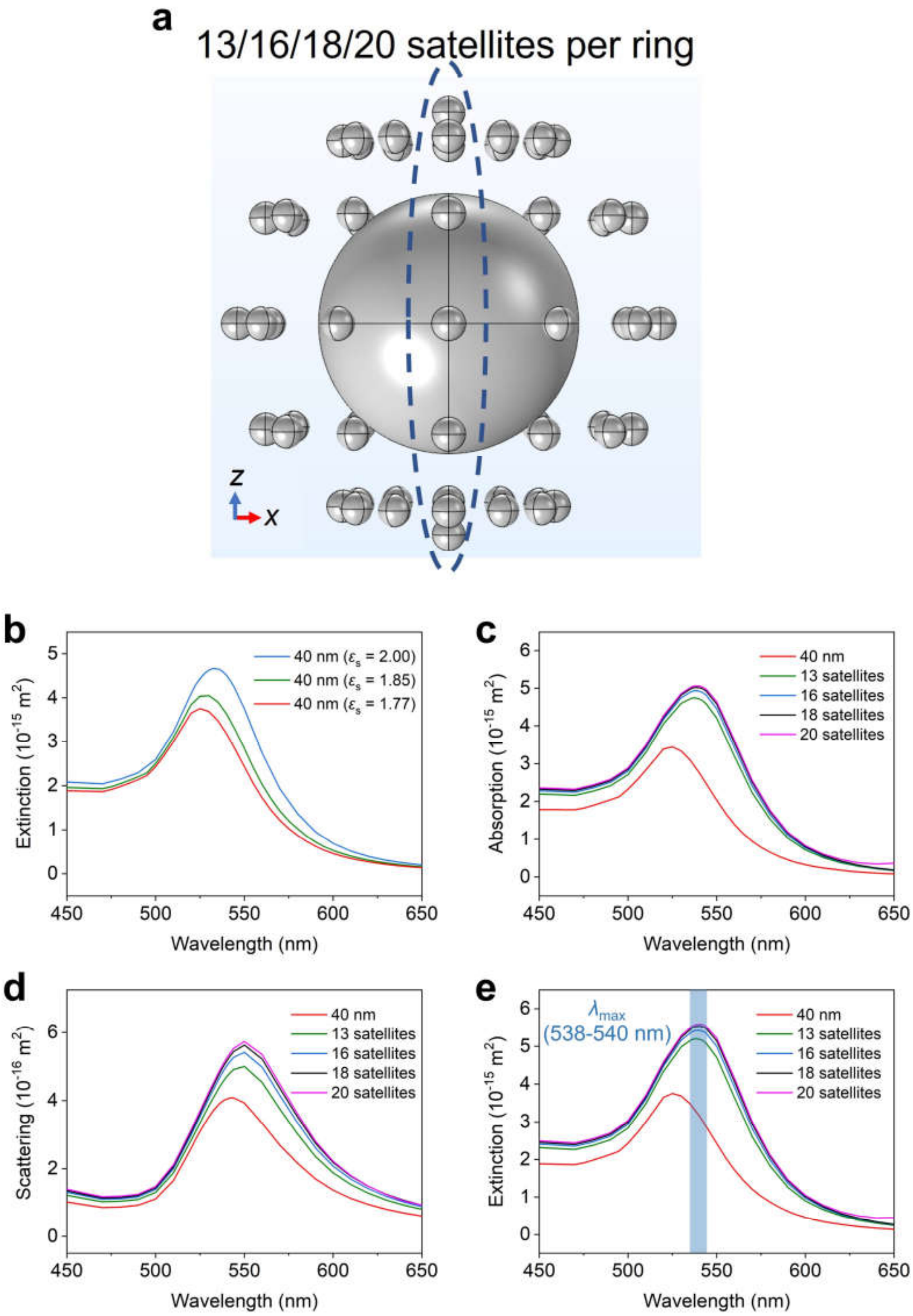

Supplementary Fig. 46. Influence of structural changes on the optical responses of superstructure. a, 3D model of superstructure. **b,** Calculated extinction spectra of 40 nm AuNP with different dielectric constant of surroundings (ε_S). **c-e,** Calculated absorption (**c**), scattering (**d**) and extinction (**e**) spectra of 40 nm AuNP ($\varepsilon_S = 1.77$) and superstructure with different number of satellites per ring (the core-satellite gap is 10 nm, and the effective dielectric constant is set 2.00). Source data are provided as a Source Data file.

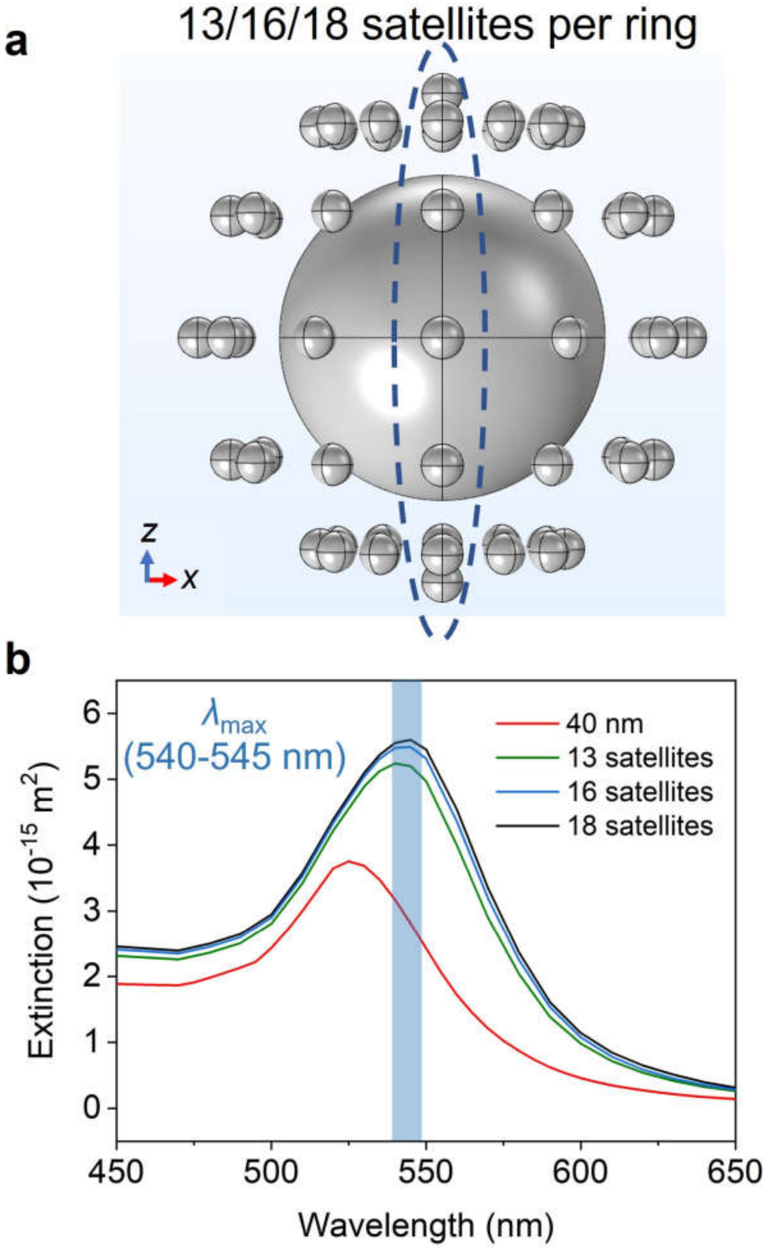

Supplementary Fig. 47. Influence of structural changes on the optical responses of superstructure. Schematic of superstructure (**a**) and calculated extinction spectra (**b**) of 40 nm AuNP ($\varepsilon_S = 1.77$) and superstructure with different number of satellites per ring (the core-satellite gap is 7.5 nm, and the effective dielectric constant is set 2.00). Source data are provided as a Source Data file.

Supplementary Table 1. Enhancement factor, ρ and g factor of Au complexes (5 nm)

Au complexes (5 nm)	14nt	7nt	2nt
$CD_{complex}$ at 520 nm (Before adding DTT)	3.05	3.52	11.46
$CD_{cBDP\text{-}DNA}$ at 504 nm (After adding DTT)	0.75	0.86	1.62
Enhancement factor ($CD_{complex}/CD_{cBDP\text{-}DNA}$)	4.07	4.09	7.07
ρ	25	23	22
g factor (CD/Absorption/33000)	2.00×10^{-4}	2.22×10^{-4}	3.99×10^{-4}

The CD and absorption intensities are based on Supplementary Fig. 23. The cBDP-DNA concentration is determined by the standard curve in Supplementary Fig. 22. It is clear that the cBDP-DNA surface concentrations in three complexes are very close. Thus, the comparison and analysis of CD spectra in main text Fig. 1 is reasonable.

Supplementary Table 2. Enhancement factor, ρ and g factor of Au complexes (5 nm, 2nt)

Au complexes (5 nm, 2nt)	ρ_1	ρ_2	ρ_3	ρ_4	ρ_5
$CD_{complex}$ at 520 nm (Before adding DTT)	0.19	1.31	3.54	11.46	8.43
$CD_{cBDP\text{-}DNA}$ at 504 nm (After adding DTT)	0.17	0.54	0.95	1.62	1.41
Enhancement factor ($CD_{complex}/CD_{cBDP\text{-}DNA}$)	1.12	2.42	3.72	7.07	5.98
ρ	4	12	18	22	30
g factor (CD/Absorption/33000)	9.44×10^{-6}	6.73×10^{-5}	1.65×10^{-4}	3.99×10^{-4}	3.60×10^{-4}

The DNA density (surface concentration in our case) of spherical nucleic acids obtained by freezing method has been studied systematically by Hao et. al.[6] 5 nm AuNPs could load 27 DNA of 21-base length, 20 DNA of 59-base length and 16 DNA of 89-base length. In our case, 5 nm AuNPs loading of 30 cBDP-DNA (2nt) of 30-base length is reasonable.

Supplementary Table 3. Summary of internal ratio in CD spectra

Sample[a,b,c]	Internal ratio[d]	Sample	Internal ratio	Sample	Internal ratio
5 nm, 2nt low ρ	3.94	5 nm, 2nt[e] ρ_1	/	D1	1.65
5 nm, 7nt low ρ	3.68	5 nm, 2nt ρ_2	3.74	D2	3.05
5 nm, 14nt low ρ	1.64	5 nm, 2nt ρ_3	5.62	D3	3.12
5 nm, 2nt high ρ	7.61	5 nm, 2nt ρ_4	7.61	D4	2.85
5 nm, 7nt high ρ	3.24	5 nm, 2nt ρ_5	4.24	D5	2.75
5 nm, 14nt high ρ	2.41	cBDP-DNA (2nt) (25 μM)	1.96	/	/

a. Low ρ: 20 μL cBDP-DNA (2nt) (25 μM), 41.5 μL cBDP-DNA (7nt) (12 μM) or 32.5 μL cBDP-DNA (14nt) (15.3 μM) added to AuNPs solution during preparation in main text Methods.
b. Middle ρ: 40 μL cBDP-DNA (2nt) (25 μM), 83 μL cBDP-DNA (7nt) (12 μM) or 65 μL cBDP-DNA (14nt) (15.3 μM) added to AuNPs solution during preparation in main text Methods.
c. High ρ: 80 μL cBDP-DNA (2nt) (25 μM), 166 μL cBDP-DNA (7nt) (12 μM) or 130 μL cBDP-DNA (14nt) (15.3 μM) added to AuNPs solution during preparation in main text Methods.
d. For hybrid complexes, internal ratio = $|CD_{520\ nm}/CD_{340\ nm}|$. For cBDP-DNA, internal ratio = $|CD_{504\ nm}/CD_{340\ nm}|$.
e. The $CD_{340\ nm}$ intensity is too weak to calculate internal ratio.

The difference in the internal ratio suggests that the intermolecular exciton correlations contribute to CD enhancement, which strongly rely on the inter-distance between molecules.

Supplementary Table 4. Fitted lifetime constants for cBDP-DNA monomer and D1-D3

	A@514 nm	B@514 nm	C@514 nm	D@514 nm
τ_1 (ps)	1.8	2.36	0.36	0.25
τ_2 (ps)	\	\	4.8	3.5
τ_3 (ps)	36	20	81	93
τ_4 (ps)	3272	1666	2884	3914

Supplementary Table 5. DNA sequences for conjugation of DNA strands and cBDP

DNA strands	Sequence
**Int NH_2 dT-14nt**	TTTTTTTTTTTTTTTT/**Int NH_2 dT**/TTTTTTTTTTTTT-Dithiol
**Int NH_2 dT-7nt**	TTTTTTTTTTTTTTTTTTTTTTT/**Int NH_2 dT**/TTTTTT-Dithiol
**Int NH_2 dT-2nt**	TTTTTTTTTTTTTTTTTTTTTTTTTTTT/**Int NH_2 dT**/T-Dithiol

The notes 14nt, 7nt and 2nt in strands 1-3 mean the 14th, 7th and 2nd thymine from the 3' end,

Supplementary Table 6. DNA sequences for hybrid complexes and cBDP dimers

DNA strands	Sequence
**1**	TTTTTTTTTTTTTTTT/**BODIPY-(S)-BINOL**/TTTTTTTTTTTTT-Dithiol
**2**	TTTTTTTTTTTTTTTTTTTTTTT/**BODIPY-(S)-BINOL**/TTTTTT-Dithiol
**3**	TTTTTTTTTTTTTTTTTTTTTTTTTTTT/**BODIPY-(S)-BINOL**/T-Dithiol
**4**	**BODIPY-(S)-BINOL**/TAAAAAAAAAAAAAAAAAAAAAAAAAAAAA
**5**	**BODIPY-(S)-BINOL**/TTTAAAAAAAAAAAAAAAAAAAAAAAAAAA
**6**	**BODIPY-(S)-BINOL**/TTTTAAAAAAAAAAAAAAAAAAAAAAAAAA
**T_{30}**	TTTTTTTTTTTTTTTTTTTTTTTTTTTTTT-Dithiol
**T_6**	TTTTTT-Dithiol
**BHQ1-A_{30}**	**BHQ1**/AAAAAAAAAAAAAAAAAAAAAAAAAAAAAA

DNA strands 1-3 are cBDP-DNA conjugates, namely, cBDP-DNA (14nt), cBDP-DNA (7nt) and cBDP-DNA (2nt) respectively. The notes 14nt, 7nt and 2nt in strands 1-3 mean the 14th, 7th and 2nd thymine from the 3' end. DNA strands 4-6 are also cBDP-DNA conjugates, which are used to assemble different cBDP dimers with DNA strands 1-3, as shown in main text Fig. 3a. T_{30} and T_6 strands are used as DNA spacer co-modified on AuNPs. BHQ1-A_{30} acts as a quencher for Förster resonance energy transfer (FRET) test in Supplementary Fig. 16.

Supplementary Table 7. DNA sequences for core particles

DNA strands	Sequence
**A_{30}**	AAAAAAAAAAAAAAAAAAAAAAAAAAAAAA-Dithiol
**$(ACT)_6$**	ACTACTACTACTACTACT-Dithiol

AuNPs were co-modified with DNA strands A_{30} and spacer $(ACT)_6$ to construct core complexes.

Supplementary References

1. Sánchez-Carnerero, E. M., Moreno, F., Maroto, B. L., Agarrabeitia, A. R., Ortiz, M. J., Vo, B. G., Muller, G. & Moya, S. d. l. Circularly polarized luminescence by visible-light absorption in a chiral *O*-BODIPY dye: unprecedented design of CPL organic molecules from achiral chromophores. *J. Am. Chem. Soc.* **136**, 3346-3349 (2014).
2. Liu, Z., Jiang, Z., He, C., Chen, Y. & Guo, Z. Circularly polarized luminescence from axially chiral binaphthalene-bridged BODIPY. *Dyes Pigm.* **181**, 108593 (2020).
3. Sebastian, E., Philip, A. M., Benny, A. & Hariharan, M. Null exciton splitting in chromophoric Greek Cross (+) aggregate. *Angew. Chem. Int. Ed.* **57**, 15696-15701 (2018).
4. Johnson, P. B. & Christy, R. W. Optical constants of the noble metals. *Phys. Rev. B* **6**, 4370 (1972).
5. Kraus, W. A. & Schatz, G. C. Plasmon resonance broadening in small metal particles. *J. Chem. Phys.* **79**, 6130–6139 (1983).
6. Hao, Y., Li, Y., Song, L. & Deng, Z. Flash synthesis of spherical nucleic acids with record DNA density. *J. Am. Chem. Soc.* **143**, 3065-3069 (2021).
