# Supplementary material for: Collective chiroptical activity through the interplay of excitonic and charge-transfer effects in localized plasmonic fields
Source: Nat Commun. 2024 Jun 6;15:4846. doi: 10.1038/s41467-024-49086-3 (PMC11156920; doi:10.1038/s41467-024-49086-3)
Supplement: Supplementary file 3 — Description of Additional Supplementary Files [file 41467_2024_49086_MOESM3_ESM.pdf]

Description of Additional Supplementary Files

File Name: Supplementary Data 1

Description: LCMS chromatogram and ESI mass spectrum of cBDP-DNA conjugates

File Name: Supplementary Data 2

Description: DFT data of cBDP Dimer 1-3

File Name: Supplementary Data 3

Description: Electron tomography tilt images of superstructures

File Name: Supplementary Movie 1

Description: Tomography analysis of superstructures
