# Supplementary material for: Collective chiroptical activity through the interplay of excitonic and charge-transfer effects in localized plasmonic fields
Source: Nat Commun. 2024 Jun 6;15:4846. doi: 10.1038/s41467-024-49086-3 (PMC11156920; doi:10.1038/s41467-024-49086-3)
Supplement: Supplementary file 4 — Supplementary Data 1 [file 41467_2024_49086_MOESM4_ESM.pdf]

[<<]

Data File: E:\LTQ27\1228\LCQ18HPLC\H-5\1941077323-5.raw
Acquisition Date: 12/28/2023
Sample Name: TTTTTTTTTTTTTTTTTTTTTTTTTTTTTT/iCUST227-NdT/T
Sample ID: F+T01
Position: 20
Inj Vol: 30
Instrument Method: C:\Xcalibur\methods\oligo_htcs
Processing Method: C:\Xcalibur\methods\oligo_htcs

Target Mass Summary

RT (min)	Target Mass (Da)	Observed Mass (Da)	Mass Error	Intensity	% Abundance (in Spectrum)	%Purity (Estimate)	Identity	Result Code
0.44	10058.9	10058.9	0.0 Da (0.000 %)	2.17E+006	96.44	96.44	Target Mass	

Chromatogram Summary

RT (min)	Base Peak Mass (Da)	Intensity	Spectral Quality	LC/MS Peak Area	LC/MS Area Percent
0.44	10058.9	2.17E+006	ok	1.98E+006	100.00

[<<] [Top]

LC/MS Chromatogram of F+T01:
TIC

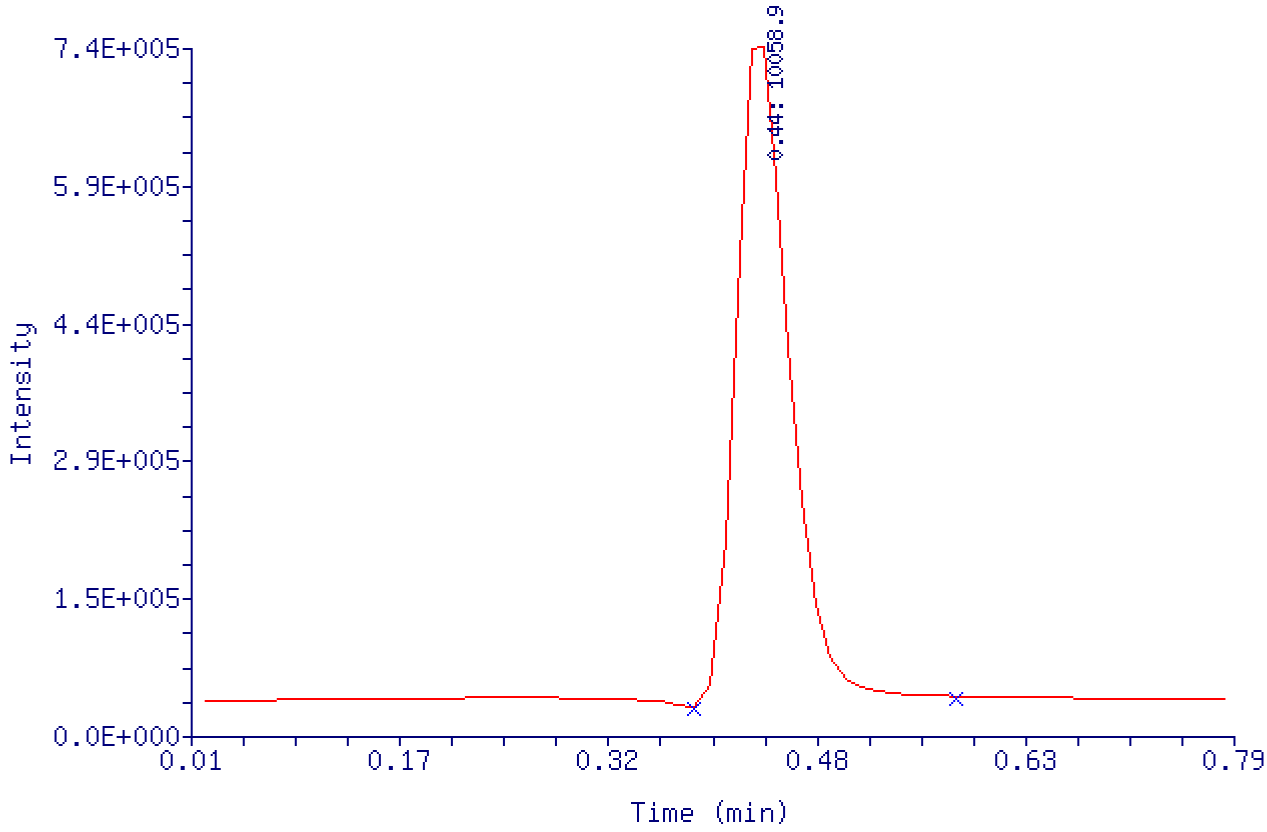

[<<] [Top] [Deconvolution] [Zoom Deconvolution] [Deconvolution Peak Report] [View Data] [Log File]

ESI Mass Spectrum of F+T01, RT = 0.44 min:

Scan Mode: - c ESI Full ms

Scans Averaged: 33-43 Minus: 9-30, 48-57

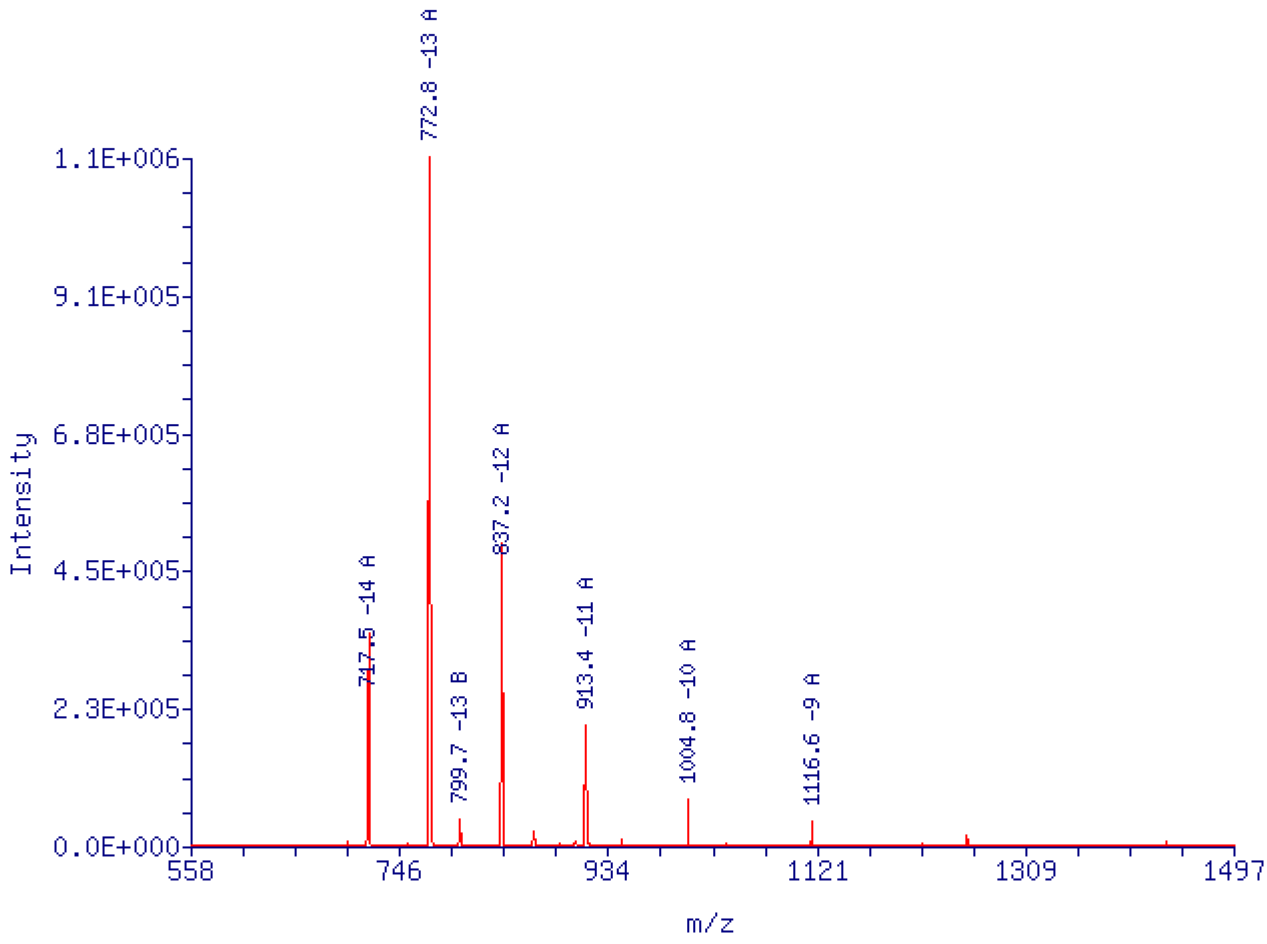

[<<] [Top] [ESI Mass Spectrum] [Zoom Deconvolution] [Deconvolution Peak Report] [View Data] [Log File]

Deconvoluted Mass Spectrum of F+T01, RT = 0.44 min:

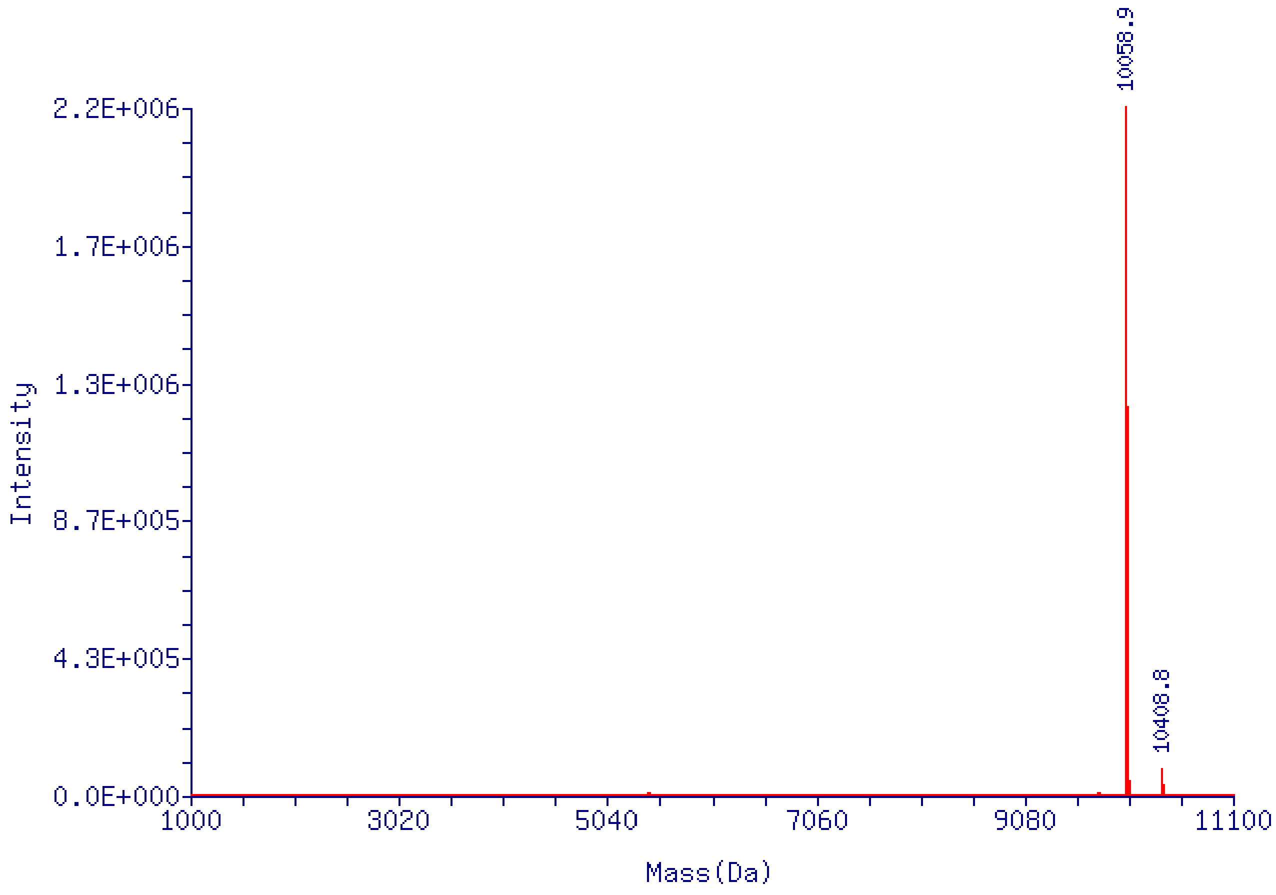

[<<] [Top] [ESI Mass Spectrum] [Deconvolution] [Deconvolution Peak Report] [View Data] [Log File]

Zoom Deconvoluted Mass Spectrum of F+T01, RT = 0.44 min:

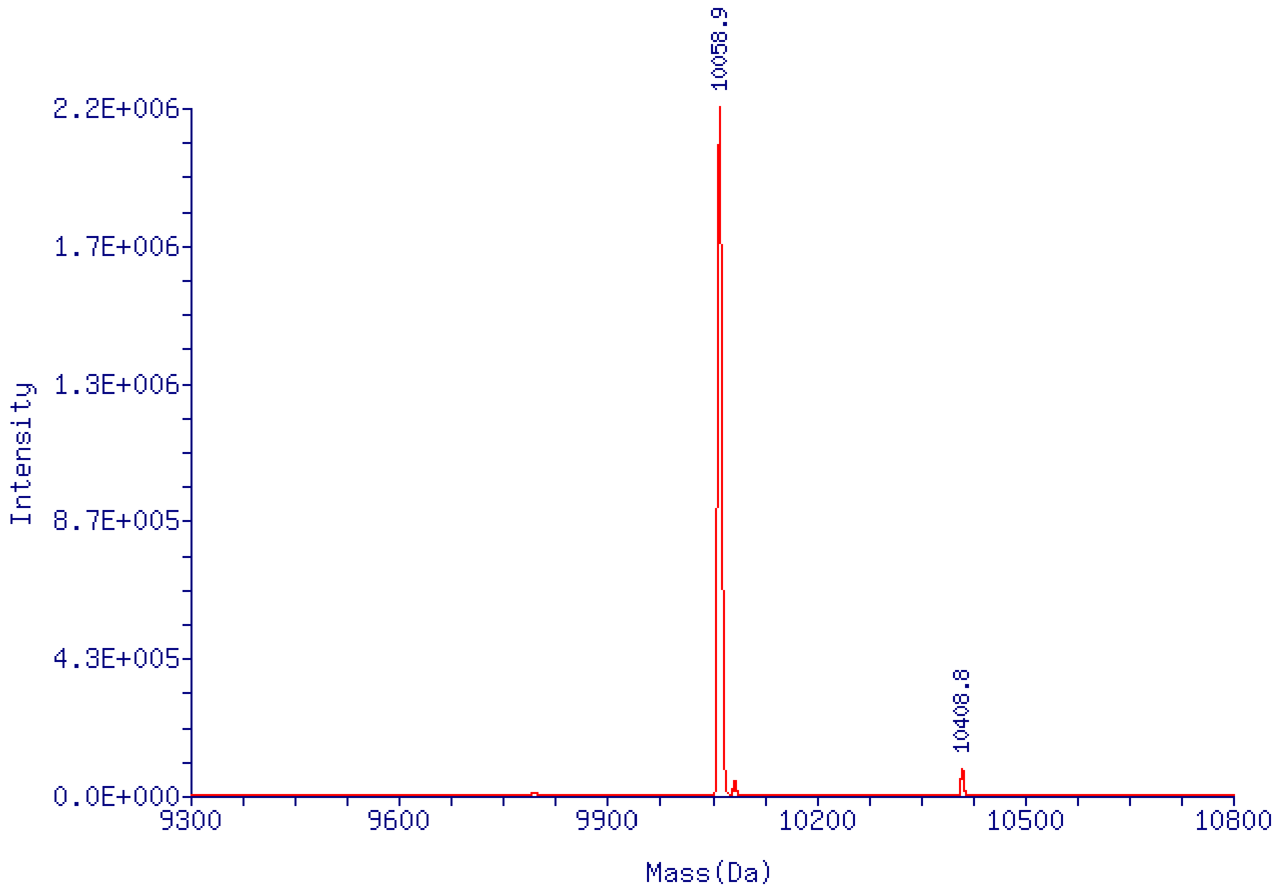

[<<] [Top]

Result Code	Indication
	Target mass found in chromatogram as the most abundant component within 0.05% mass error tolerance
	Target mass found as a major component or as a minor component with other target masses, but NOT as most abundant component in chromatogram
	Target mass found in chromatogram with either or all of the following:<br>(a) other non-target components present in spectrum > 30% abundance<br>(b) low spectral quality (low intensity and/or score)
	Target mass found in chromatogram, but NOT as the most abundant in any of the chromatographic peaks
	Target mass NOT found in chromatogram within 0.05% mass error tolerance
	No target masses specified

ZNova 3.0.2 ©2001-2013 Novatia, LLC

[<<]
Data File: E:\LTQ7\1130\HPLC\H-7\1935465335-1.raw
Acquisition Date: 11/30/2022
Sample Name: TTTTTTTTTTTTTTTTTTTTTTTTTTTTTT/iCUST195-NdT/T
Sample ID: F+T01
Position: 27
Inj Vol: 30
Instrument Method: C:\Xcalibur\methods\oligo_htcs.meth
Processing Method: C:\Xcalibur\methods\oligo_htcs

Target Mass Summary

RT (min)	Target Mass (Da)	Observed Mass (Da)	Mass Error	Intensity	% Abundance (in Spectrum)	% Purity (Estimate)	Identity	Result Code
0.39	10058.9	10056.4	-2.5 Da (-0.025 %)	9.79E+006	96.77	96.77	Target Mass	

Chromatogram Summary

RT (min)	Base Peak Mass (Da)	Intensity	Spectral Quality	LC/MS Peak Area	LC/MS Area Percent
0.39	10056.4	9.79E+006	ok	2.16E+007	100.00

[<<] [Top]
LC/MS Chromatogram of F+T01:
TIC

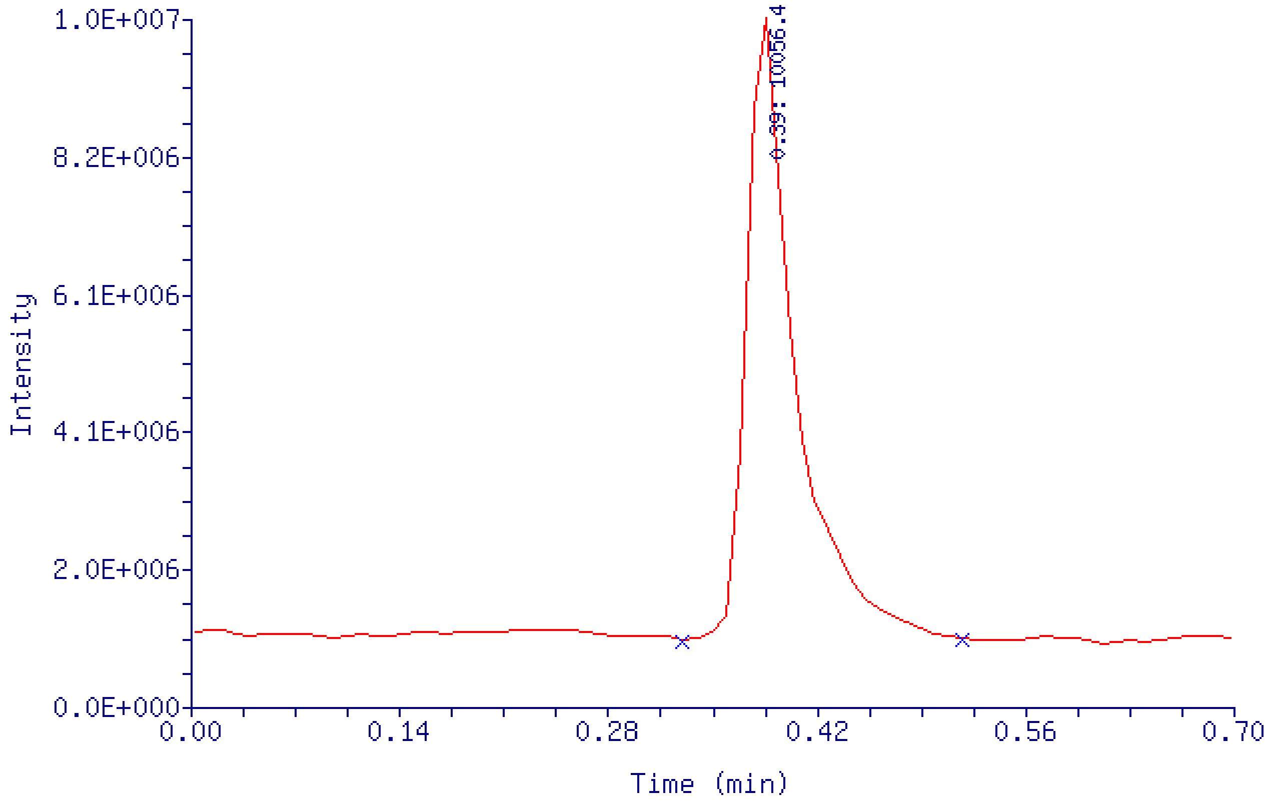

[<<] [Top] [Deconvolution] [Zoom Deconvolution] [Deconvolution Peak Report] [View Data] [Log File]

ESI Mass Spectrum of F+T01, RT = 0.39 min:

Scan Mode: - c ESI Full ms

Scans Averaged: 37-47 Minus: 27-50, 66

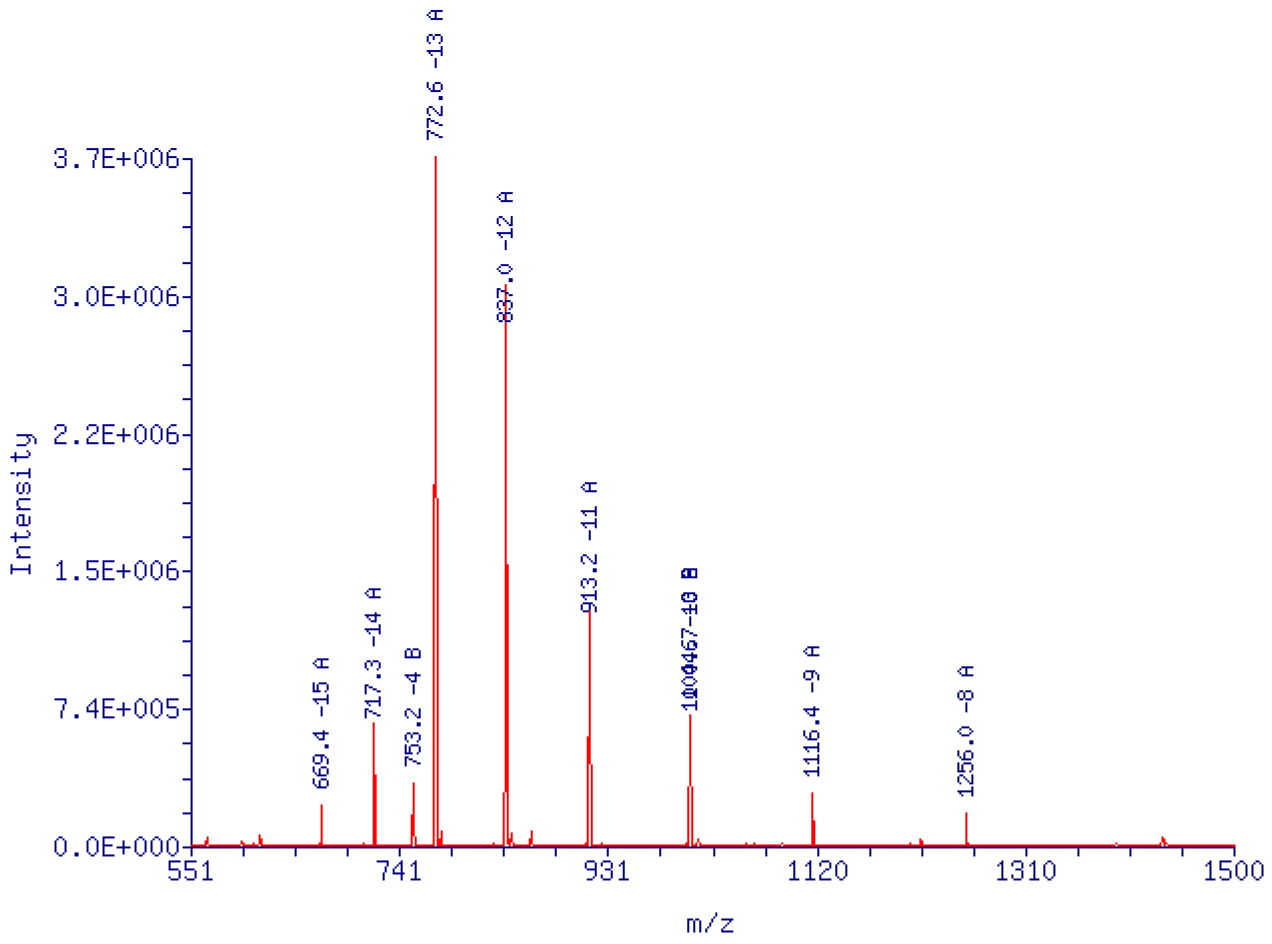

[<<] [Top] [ESI Mass Spectrum] [Zoom Deconvolution] [Deconvolution Peak Report] [View Data] [Log File]

Deconvoluted Mass Spectrum of F+T01, RT = 0.39 min:

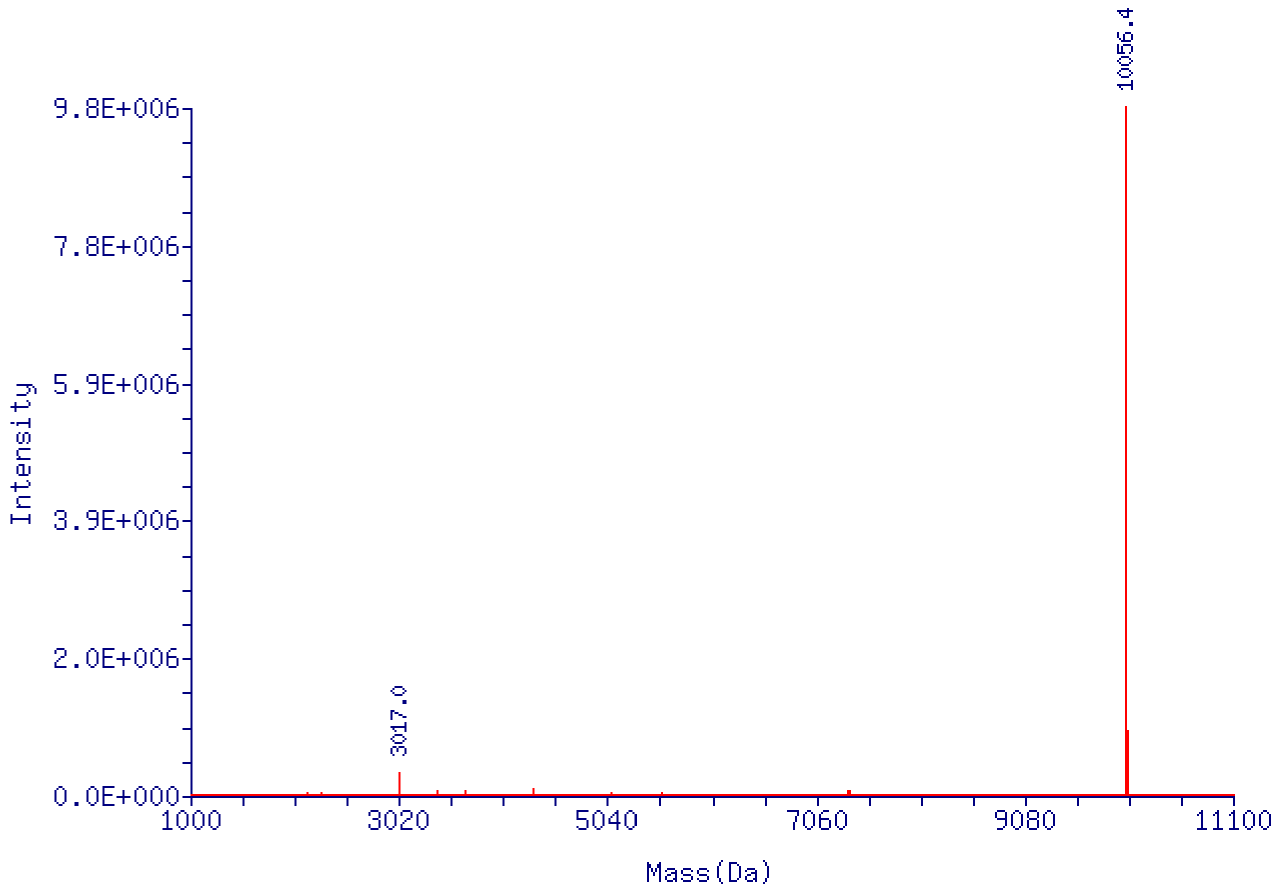

[<<] [Top] [ESI Mass Spectrum] [Deconvolution] [Deconvolution Peak Report] [View Data] [Log File]

Zoom Deconvoluted Mass Spectrum of F+T01, RT = 0.39 min:

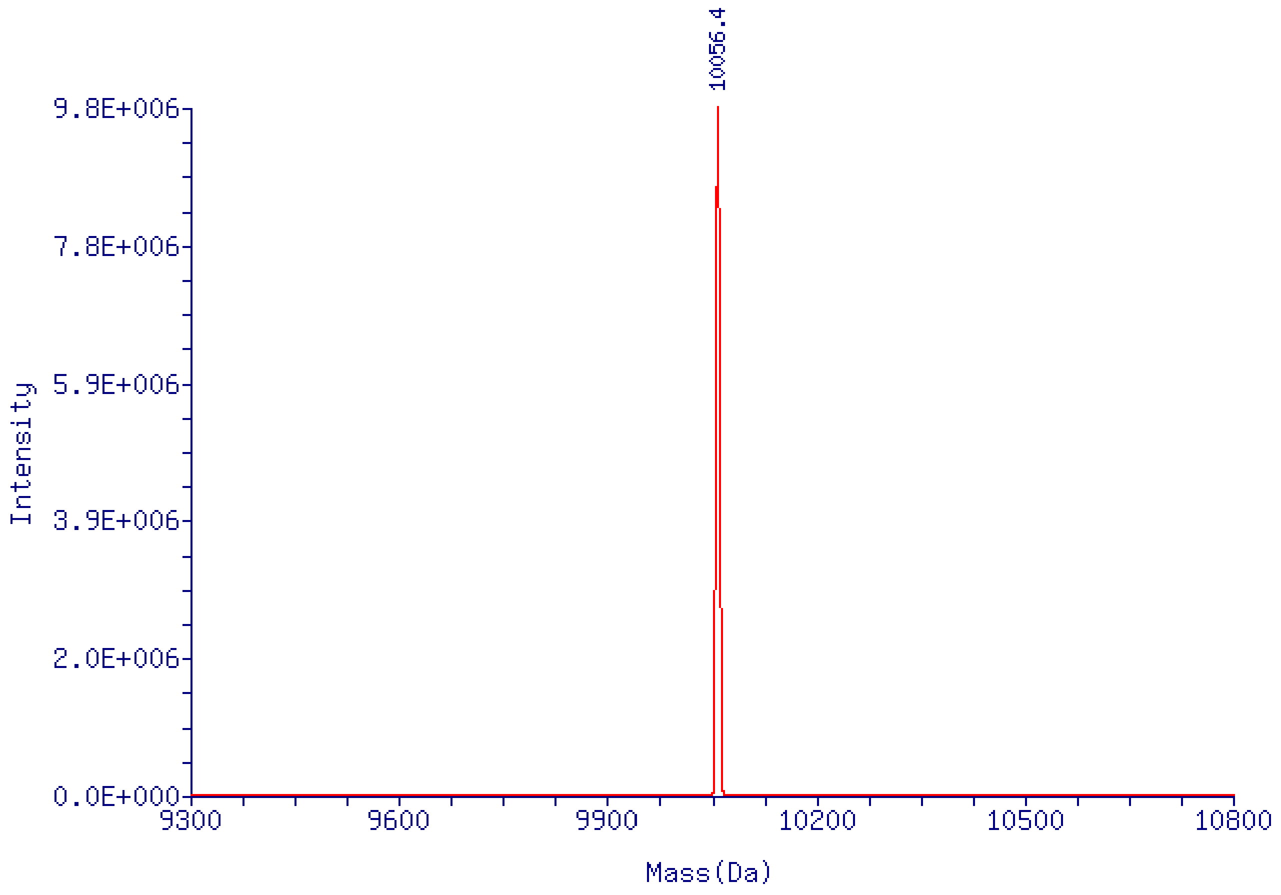

[<<] [Top]

Result Code	Indication
	Target mass found in chromatogram as the most abundant component within 0.048% mass error tolerance
	Target mass found as a major component or as a minor component with other target masses, but NOT as most abundant component in chromatogram
	Target mass found in chromatogram with either or all of the following:<br>(a) other non-target components present in spectrum > 30% abundance<br>(b) low spectral quality (low intensity and/or score)
	Target mass found in chromatogram, but NOT as the most abundant in any of the chromatographic peaks
	Target mass NOT found in chromatogram within 0.048% mass error tolerance
	No target masses specified

ZNova 3.0.2 ©2001-2013 Novatia, LLC

[<<]
Data File: E:\LTQ7\1130\HPLC\H-7\1935465334-1.raw
Acquisition Date: 11/30/2022
Sample Name: TTTTTTTTTTTTTTTTTTTTTTTTT/iCUST195-NdT/TTTTTT
Sample ID: F+T01
Position: 35
Inj Vol: 30
Instrument Method: C:\Xcalibur\methods\oligo_htcs.meth
Processing Method: C:\Xcalibur\methods\oligo_htcs

ProMass

Target Mass Summary

RT (min)	Target Mass (Da)	Observed Mass (Da)	Mass Error	Intensity	% Abundance (in Spectrum)	% Purity (Estimate)	Identity	Result Code
0.38	10058.9	10056.7	-2.2 Da (-0.022 %)	1.15E+007	100.00	100.00	Target Mass	

Chromatogram Summary

RT (min)	Base Peak Mass (Da)	Intensity	Spectral Quality	LC/MS Peak Area	LC/MS Area Percent
0.38	10056.7	1.15E+007	ok	2.01E+007	100.00

[<<] [Top]
LC/MS Chromatogram of F+T01:
TIC

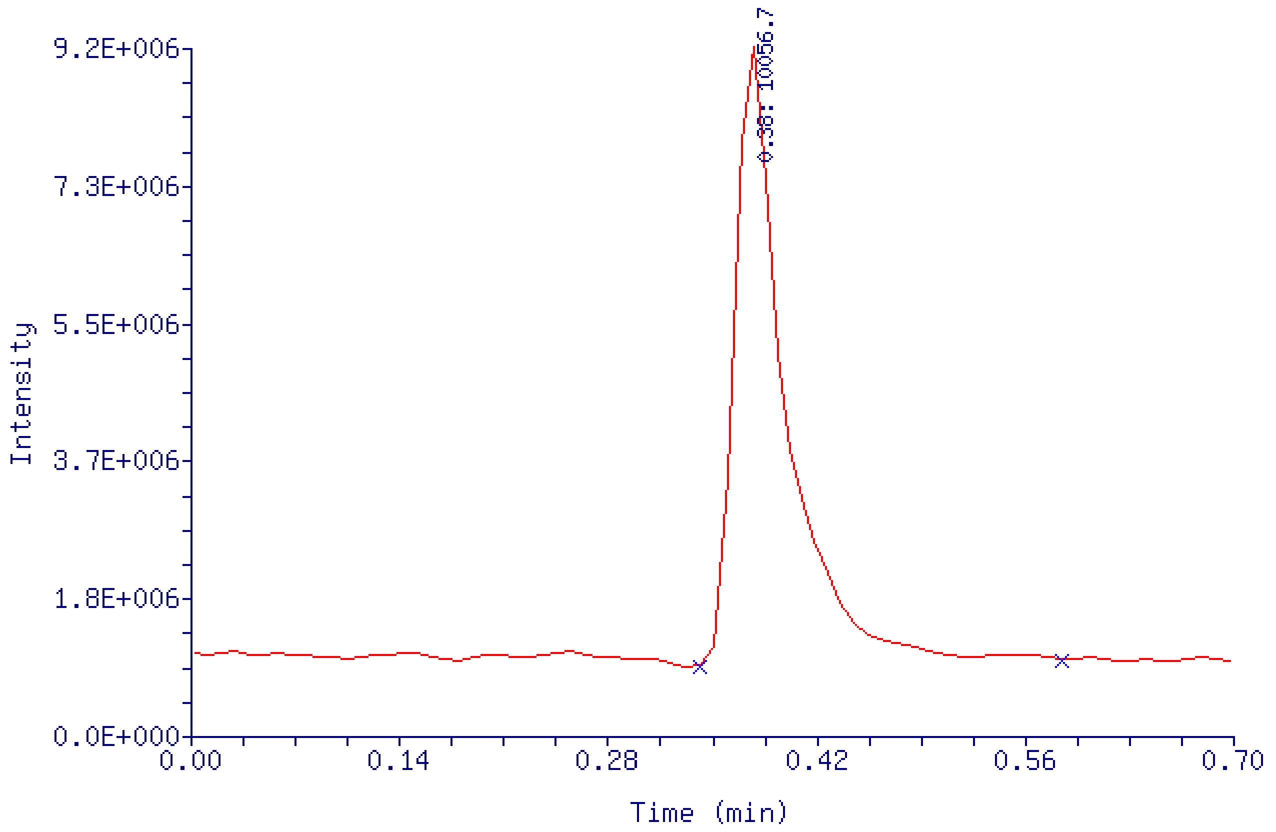

[<<] [Top] [Deconvolution] [Zoom Deconvolution] [Deconvolution Peak Report] [View Data] [Log File]

ESI Mass Spectrum of F+T01, RT = 0.38 min:

Scan Mode: - c ESI Full ms

Scans Averaged: 37-46 Minus: 27-50, 66

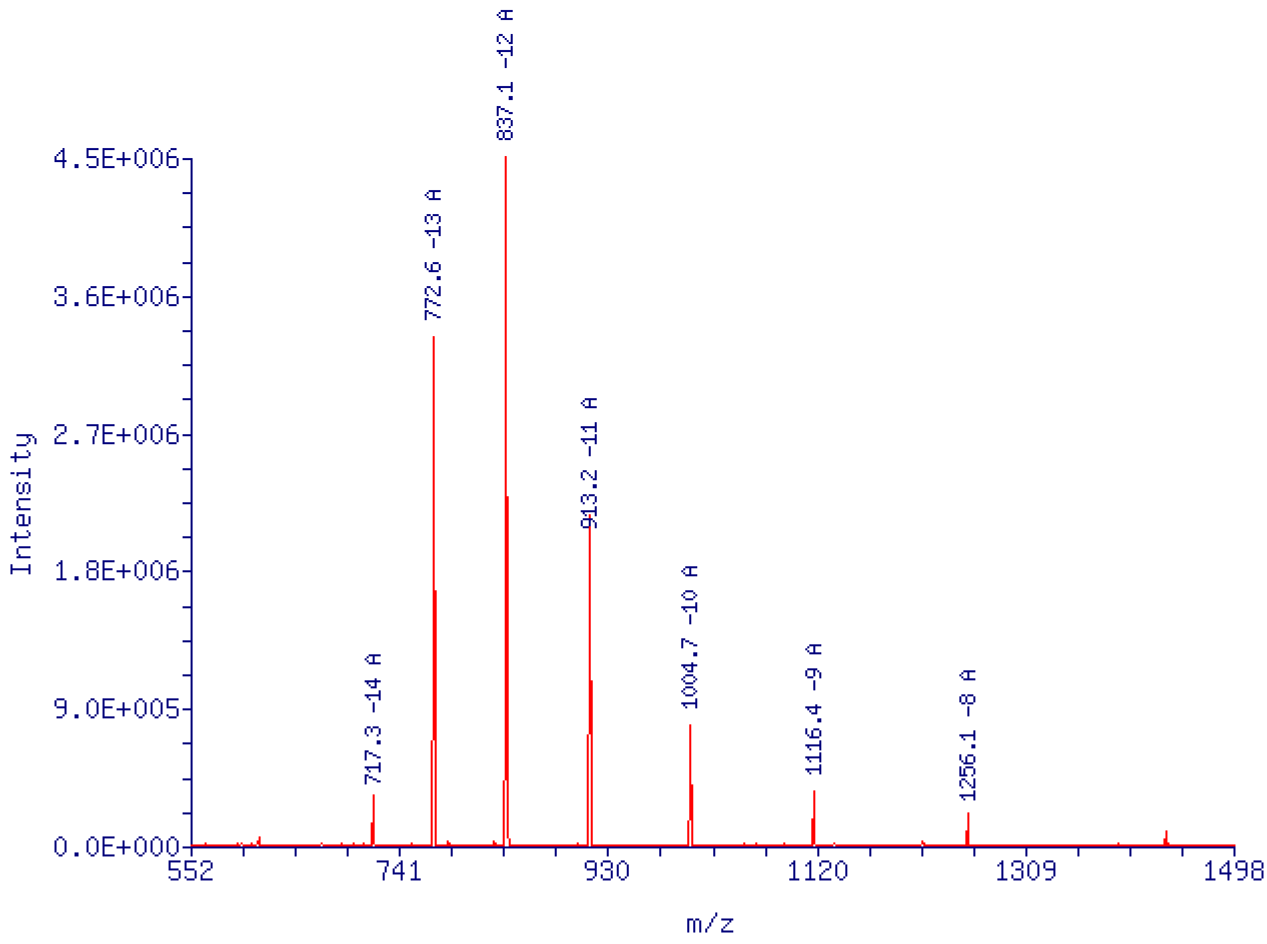

[<<] [Top] [ESI Mass Spectrum] [Zoom Deconvolution] [Deconvolution Peak Report] [View Data] [Log File]

Deconvoluted Mass Spectrum of F+T01, RT = 0.38 min:

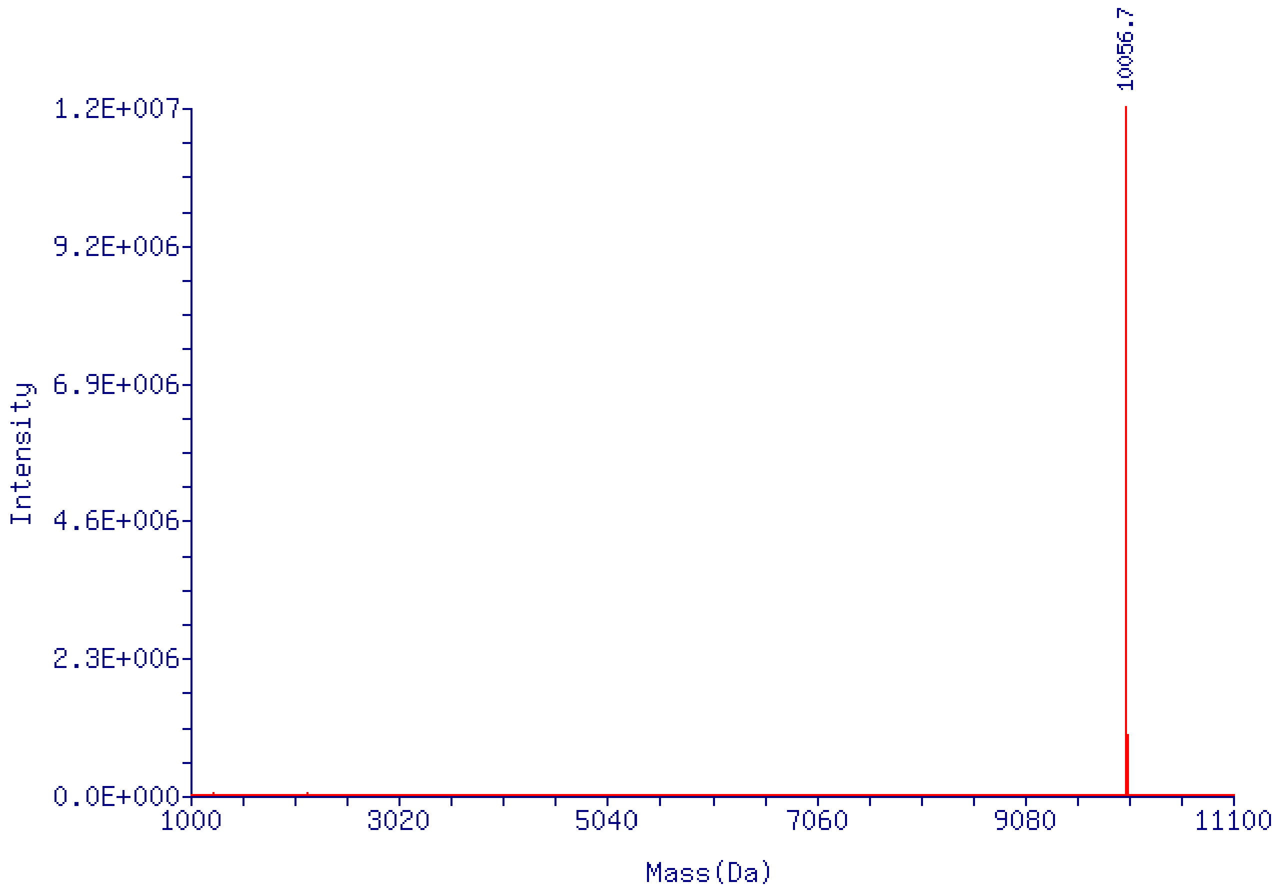

[<<] [Top] [ESI Mass Spectrum] [Deconvolution] [Deconvolution Peak Report] [View Data] [Log File]

Zoom Deconvoluted Mass Spectrum of F+T01, RT = 0.38 min:

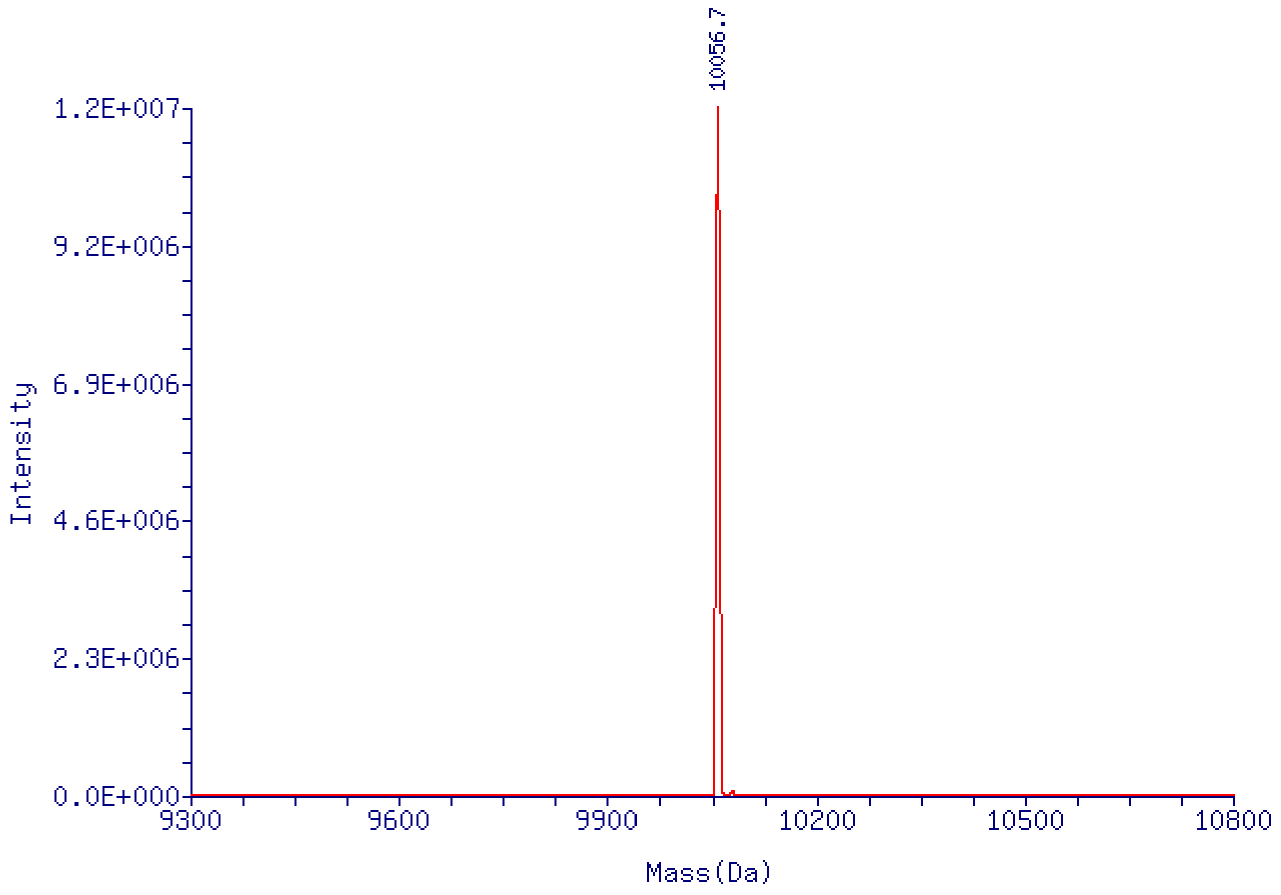

[<<] [Top]

Result Code	Indication
(green)	Target mass found in chromatogram as the most abundant component within 0.048% mass error tolerance
(blue)	Target mass found as a major component or as a minor component with other target masses, but NOT as most abundant component in chromatogram
(purple)	Target mass found in chromatogram with either or all of the following:<br>(a) other non-target components present in spectrum > 30% abundance<br>(b) low spectral quality (low intensity and/or score)
(orange)	Target mass found in chromatogram, but NOT as the most abundant in any of the chromatographic peaks
(red)	Target mass NOT found in chromatogram within 0.048% mass error tolerance
(cyan)	No target masses specified

ZNova 3.0.2 ©2001-2013 Novatia, LLC

[<<]

Data File: E:\LTQ6\1201\HPLC\H-7\1935465333-1.raw
Acquisition Date: 12/1/2022
Sample Name: TTTTTTTTTTTTTTTT/iCUST195-NdT/TTTTTTTTTTTTT
Position: 49
Inj Vol: 30
Instrument Method: C:\Xcalibur\methods\oligo_htcs
Processing Method: C:\Xcalibur\methods\oligo_htcs

ProMass

Target Mass Summary

RT (min)	Target Mass (Da)	Observed Mass (Da)	Mass Error	Intensity	% Abundance (in Spectrum)	% Purity (Estimate)	Identity	Result Code
0.41	10058.9	10060.0	1.1 Da (0.011 %)	1.65E+007	91.21	95.00	Target Mass	

Chromatogram Summary

RT (min)	Base Peak Mass (Da)	Intensity	Spectral Quality	LC/MS Peak Area	LC/MS Area Percent
0.41	10060.0	1.65E+007	ok	2.64E+007	100.00

[<<] [Top]

LC/MS Chromatogram:

TIC

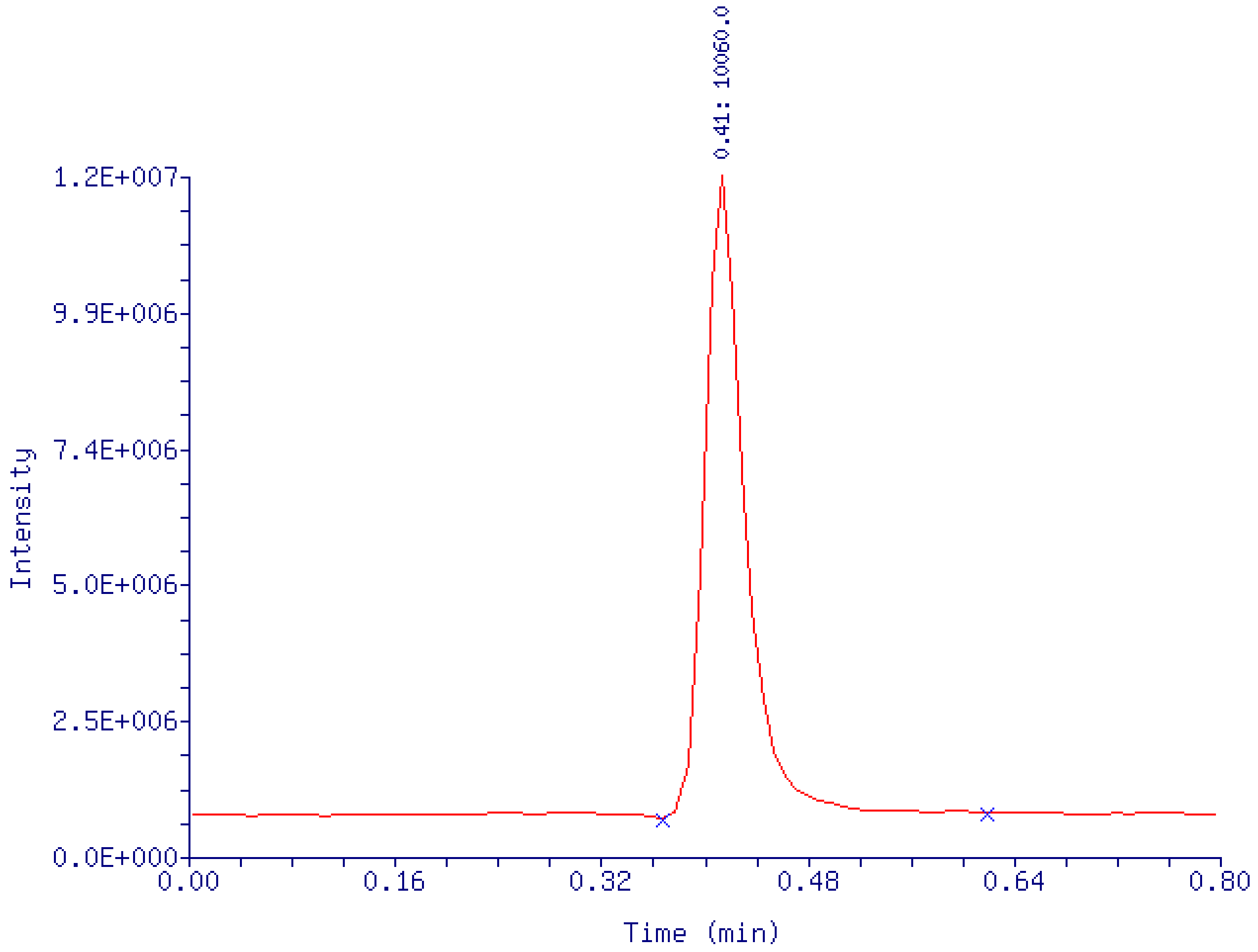

[<<] [Top] [Deconvolution] [Zoom Deconvolution] [Deconvolution Peak Report] [View Data] [Log File]

ESI Mass Spectrum , RT = 0.41 min:

Scan Mode: - c ESI Full ms

Scans Averaged: 40-50 Minus: 17-38, 66

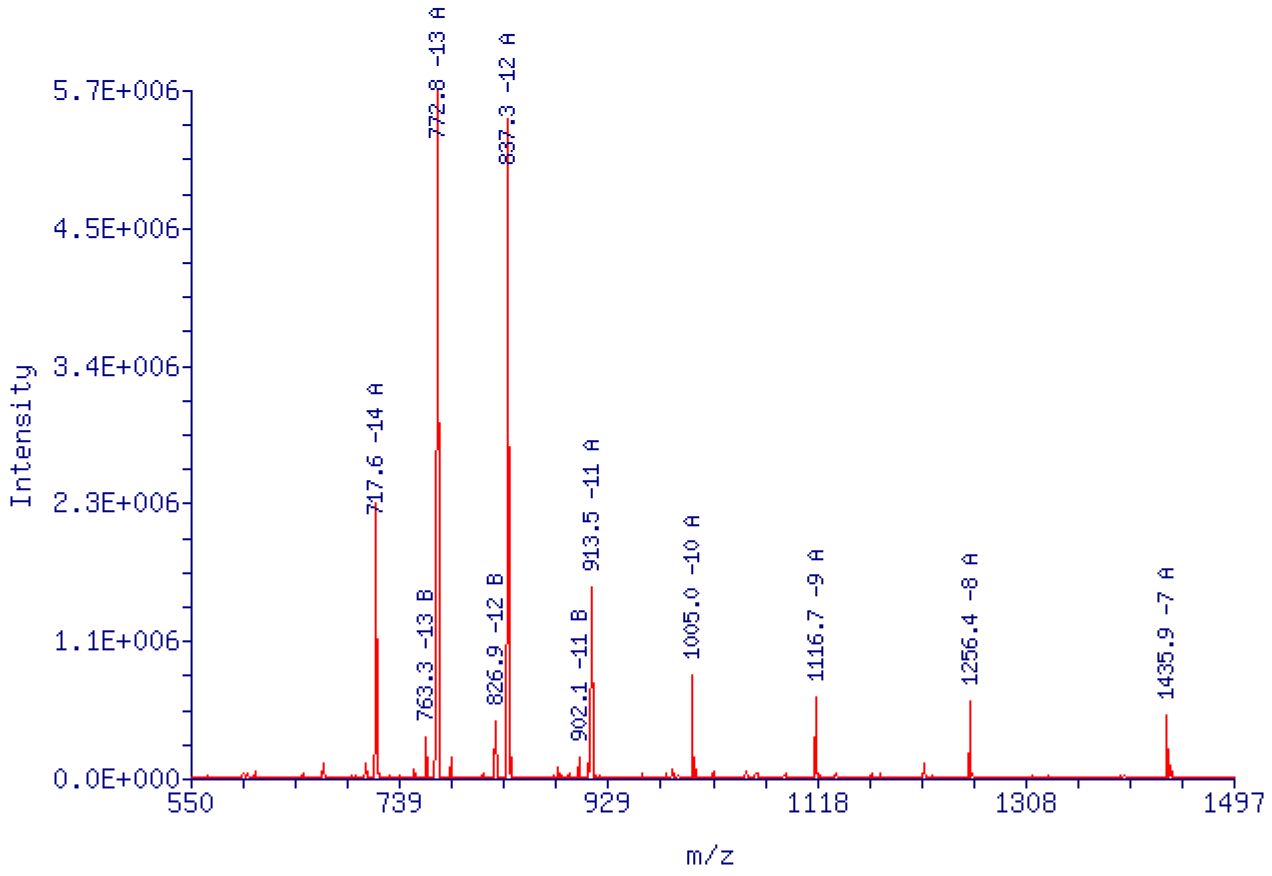

[<<] [Top] [ESI Mass Spectrum] [Zoom Deconvolution] [Deconvolution Peak Report] [View Data] [Log File]

Deconvoluted Mass Spectrum , RT = 0.41 min:

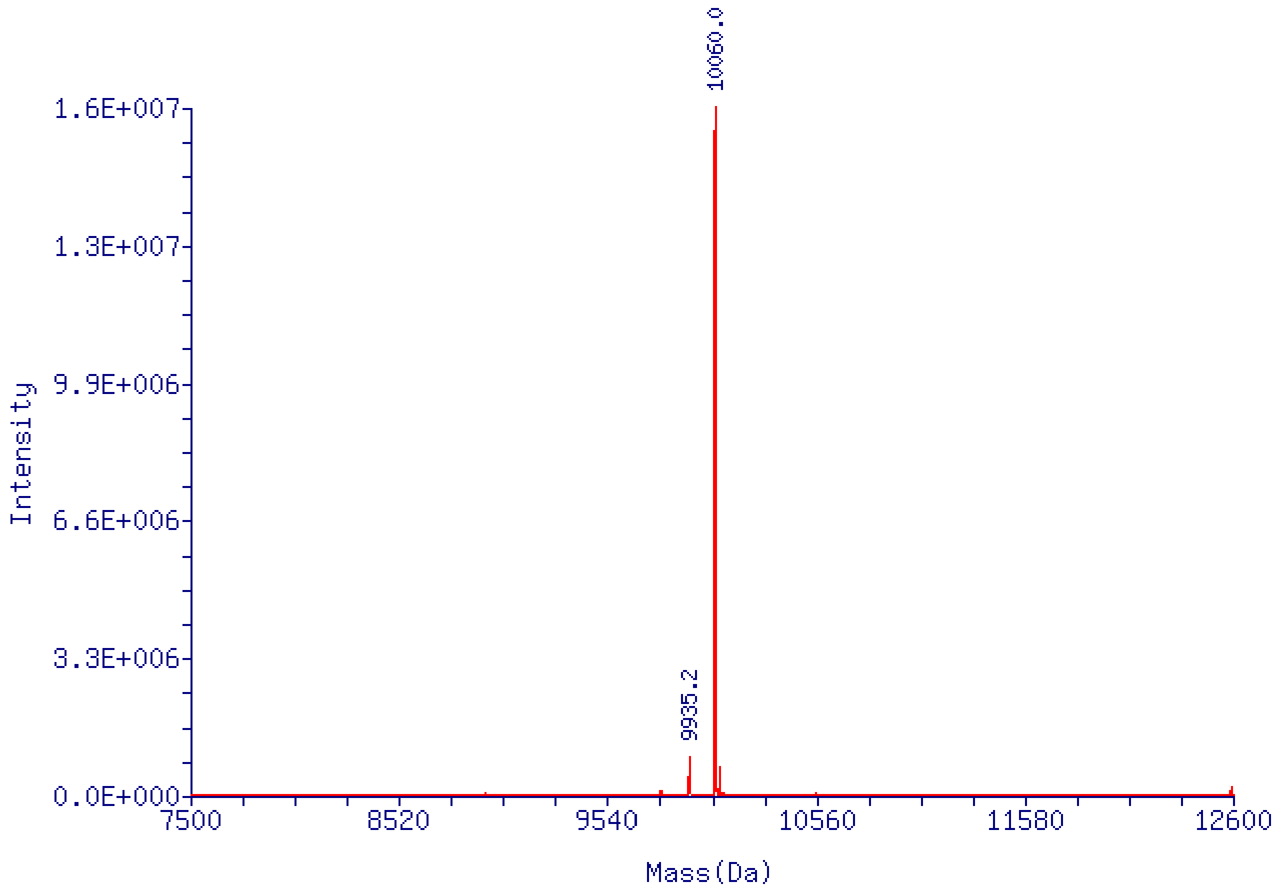

[<<] [Top] [ESI Mass Spectrum] [Deconvolution] [Deconvolution Peak Report] [View Data] [Log File]

Zoom Deconvoluted Mass Spectrum , RT = 0.41 min:

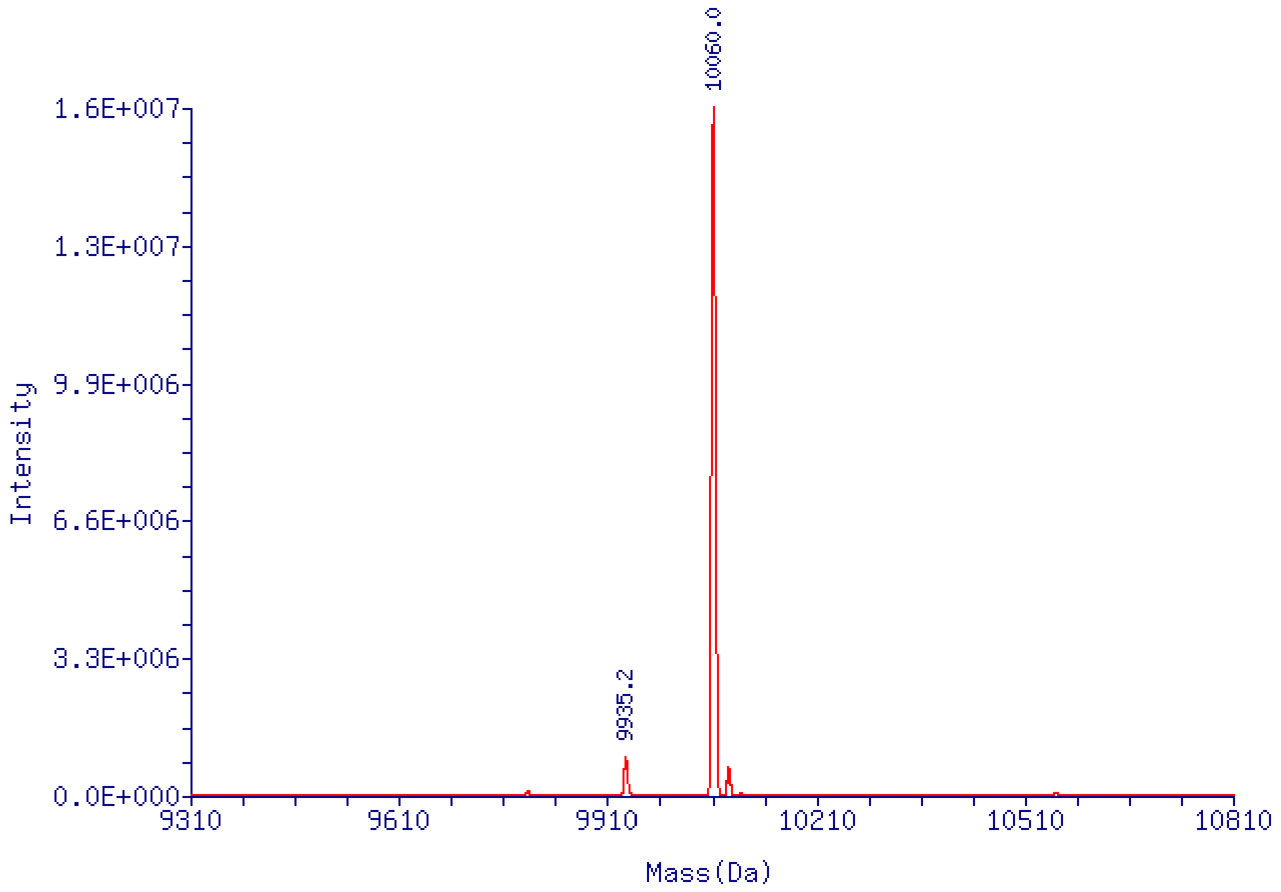

[<<] [Top]

Result Code	Indication
	Target mass found in chromatogram as the most abundant component within 0.048% mass error tolerance
	Target mass found as a major component or as a minor component with other target masses, but NOT as most abundant component in chromatogram
	Target mass found in chromatogram with either or all of the following:<br>(a) other non-target components present in spectrum > 30% abundance<br>(b) low spectral quality (low intensity and/or score)
	Target mass found in chromatogram, but NOT as the most abundant in any of the chromatographic peaks
	Target mass NOT found in chromatogram within 0.048% mass error tolerance
	No target masses specified

ZNova 3.0.2 ©2001-2013 Novatia, LLC

[<<]
Data File: E:\LTQ6\2023\0409\HPLC\H-20\1937422808-1.raw
Acquisition Date: 4/9/2023
Sample Name: TAAAAAAAAAAAAAAAAAAAAAAAAAAAAAA
Sample ID: F+T01
Position: 15
Inj Vol: 30
Instrument Method: C:\Xcalibur\methods\oligo_htcs
Processing Method: C:\Xcalibur\methods\oligo_htcs

ProMass

Target Mass Summary

RT (min)	Target Mass (Da)	Observed Mass (Da)	Mass Error	Intensity	% Abundance (in Spectrum)	% Purity (Estimate)	Identity	Result Code
0.43	10101.0	10101.6	0.6 Da (0.006 %)	2.33E+007	91.73	91.73	Target Mass	

Chromatogram Summary

RT (min)	Base Peak Mass (Da)	Intensity	Spectral Quality	LC/MS Peak Area	LC/MS Area Percent
0.43	10101.6	2.33E+007	ok	3.81E+007	100.00

[<<] [Top]
LC/MS Chromatogram of F+T01:
TIC

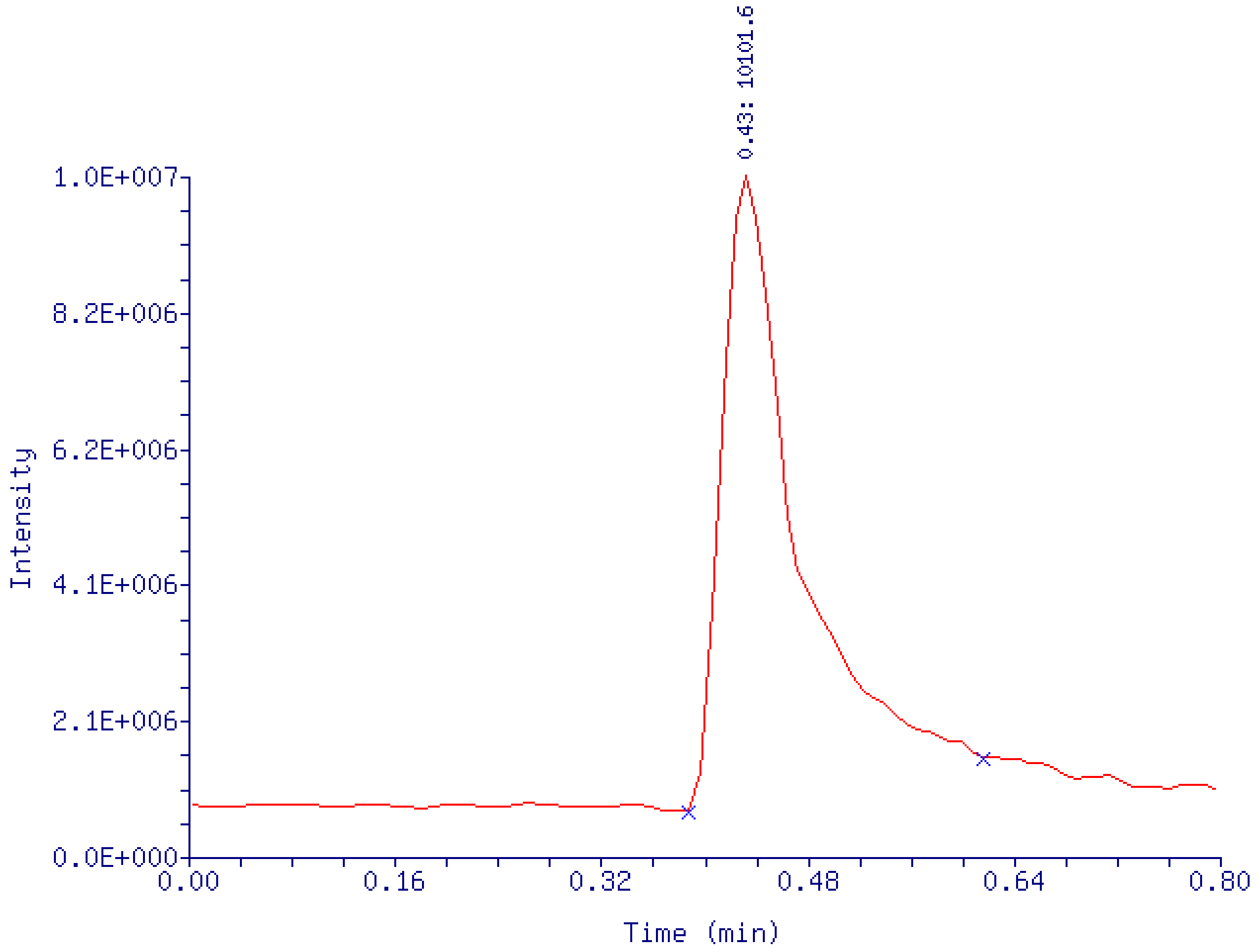

[<<] [Top] [Deconvolution] [Zoom Deconvolution] [Deconvolution Peak Report] [View Data] [Log File]

ESI Mass Spectrum of F+T01, RT = 0.43 min:

Scan Mode: - c ESI Full ms

Scans Averaged: 42-52 Minus: 17-38, 67

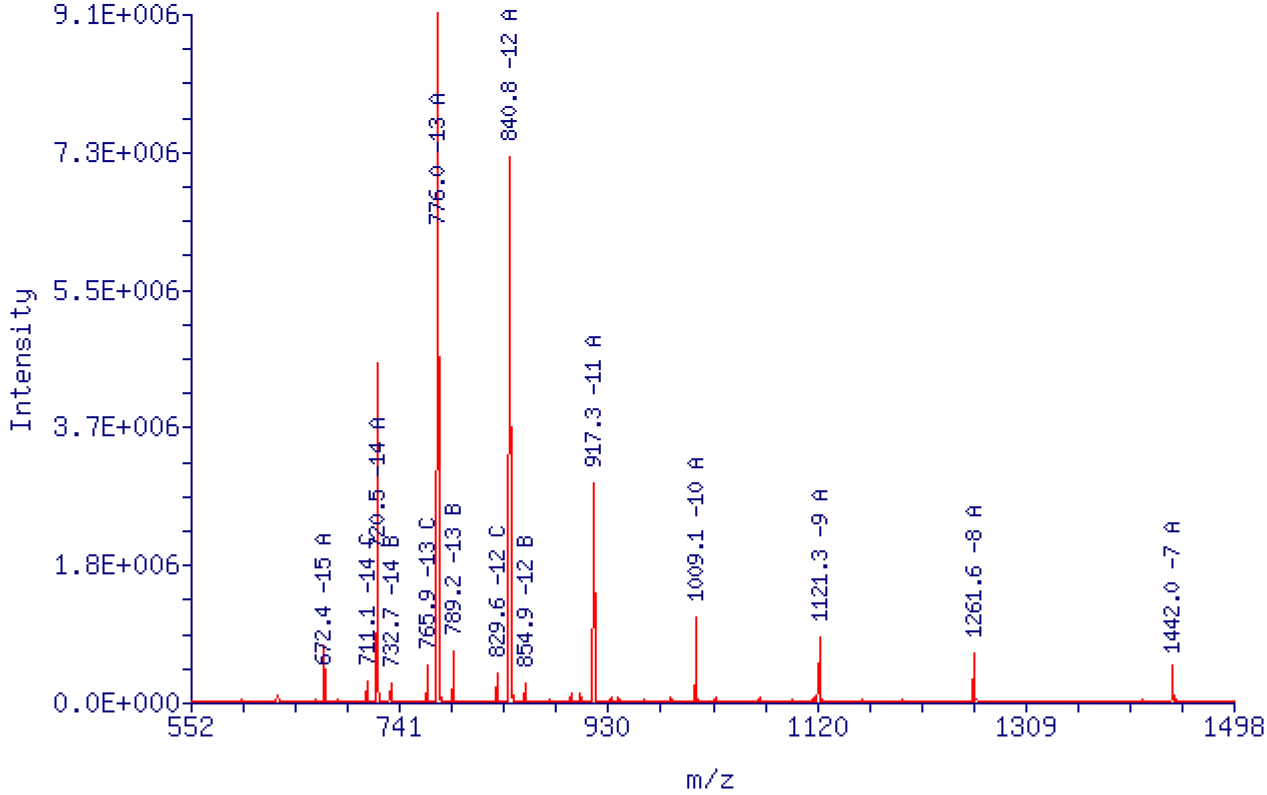

[<<] [Top] [ESI Mass Spectrum] [Zoom Deconvolution] [Deconvolution Peak Report] [View Data] [Log File]

Deconvoluted Mass Spectrum of F+T01, RT = 0.43 min:

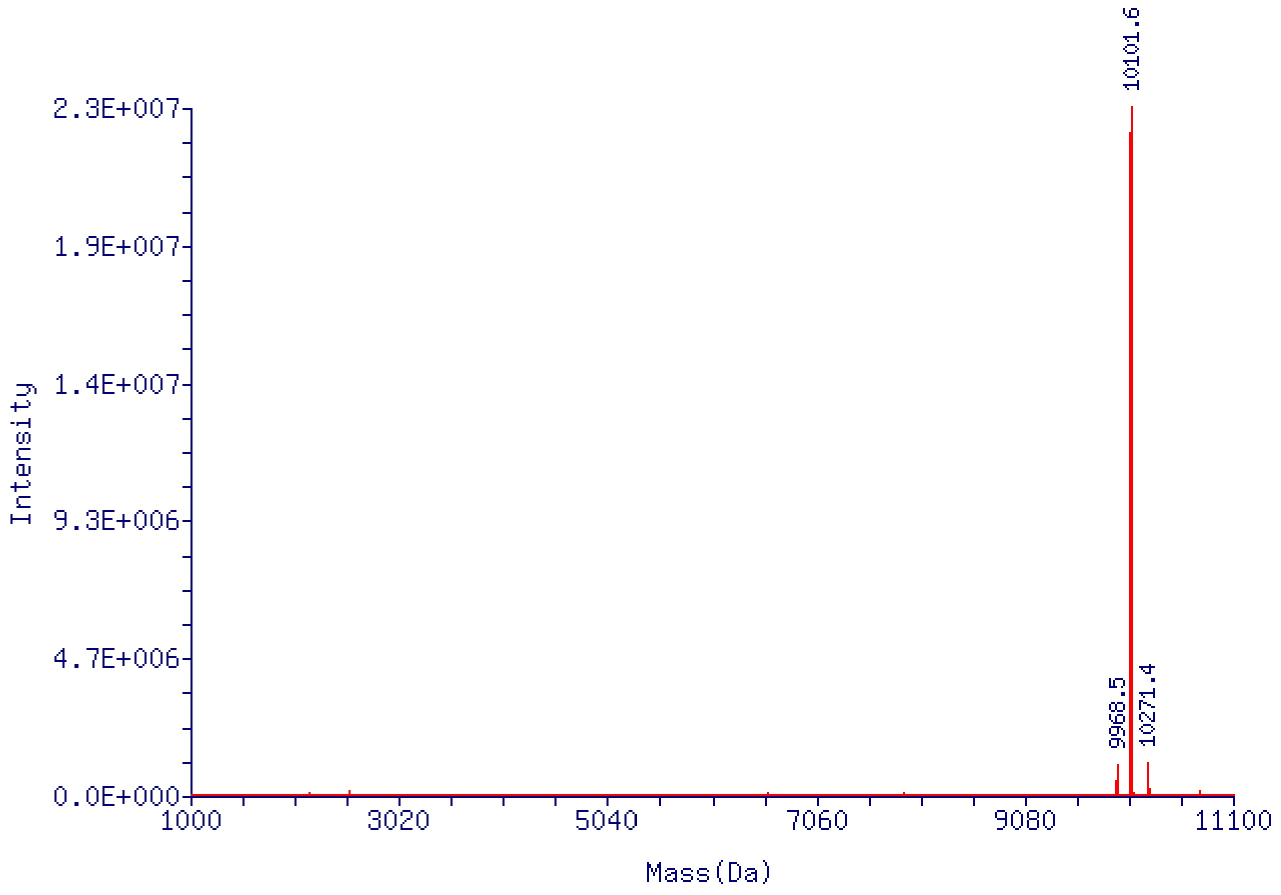

[<<] [Top] [ESI Mass Spectrum] [Deconvolution] [Deconvolution Peak Report] [View Data] [Log File]

Zoom Deconvoluted Mass Spectrum of F+T01, RT = 0.43 min:

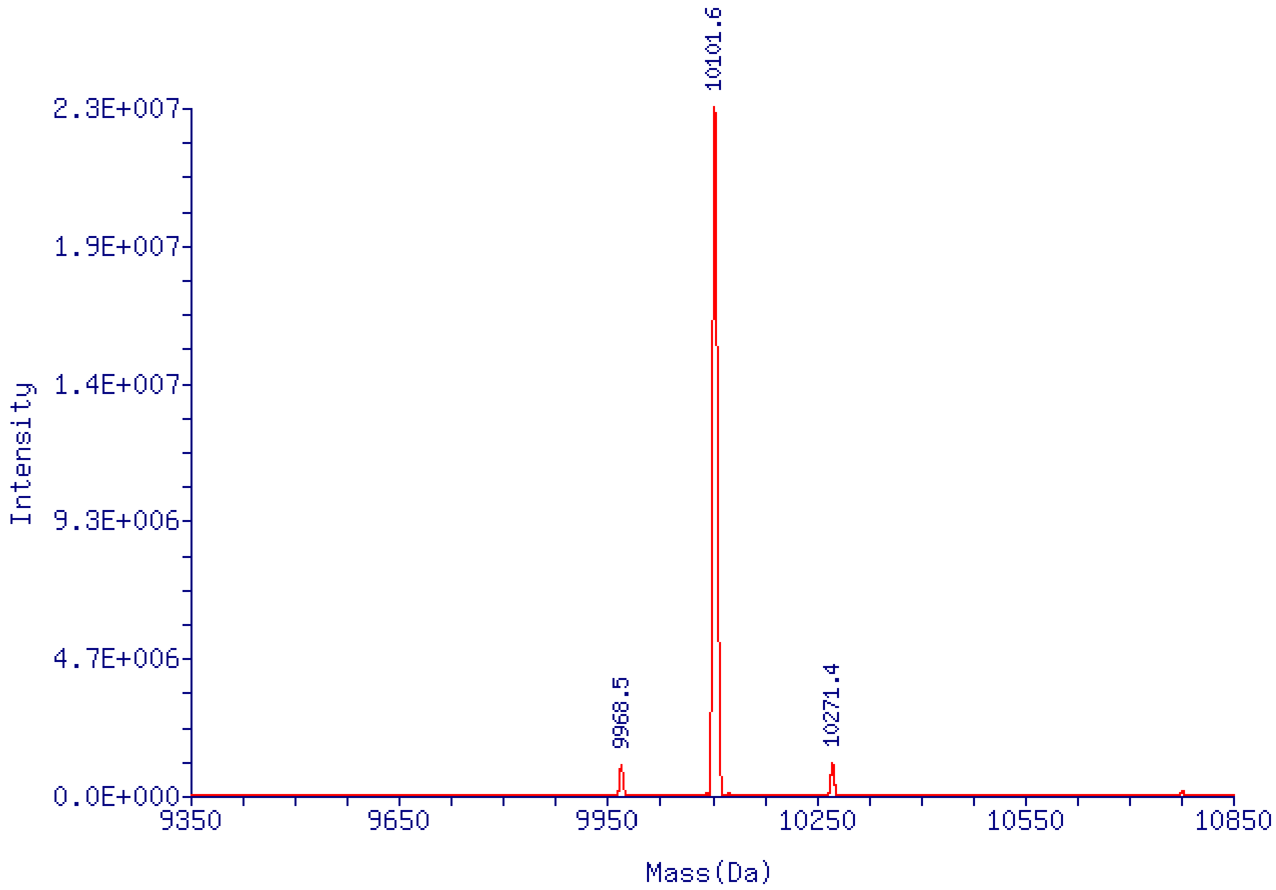

[<<] [Top]

Result Code	Indication
	Target mass found in chromatogram as the most abundant component within 0.040% mass error tolerance
	Target mass found as a major component or as a minor component with other target masses, but NOT as most abundant component in chromatogram
	Target mass found in chromatogram with either or all of the following:<br>(a) other non-target components present in spectrum > 30% abundance<br>(b) low spectral quality (low intensity and/or score)
	Target mass found in chromatogram, but NOT as the most abundant in any of the chromatographic peaks
	Target mass NOT found in chromatogram within 0.040% mass error tolerance
	No target masses specified

ZNova 3.0.2 ©2001-2013 Novatia, LLC

[<<]
Data File: E:\LTQ6\2023\0409\HPLC\H-20\1937422809-1.raw
Acquisition Date: 4/9/2023
Sample Name: TTTAAAAAAAAAAAAAAAAAAAAAAAAAAA
Sample ID: F+T01
Position: 11
Inj Vol: 30
Instrument Method: C:\Xcalibur\methods\oligo_htcs
Processing Method: C:\Xcalibur\methods\oligo_htcs

ProMass

Target Mass Summary

RT (min)	Target Mass (Da)	Observed Mass (Da)	Mass Error	Intensity	% Abundance (in Spectrum)	%Purity (Estimate)	Identity	Result Code
0.42	10082.9	10084.6	1.7 Da (0.017 %)	1.65E+007	90.65	92.76	Target Mass	

Chromatogram Summary

RT (min)	Base Peak Mass (Da)	Intensity	Spectral Quality	LC/MS Peak Area	LC/MS Area Percent
0.42	10084.6	1.65E+007	ok	3.28E+007	100.00

[<<] [Top]
LC/MS Chromatogram of F+T01:
TIC

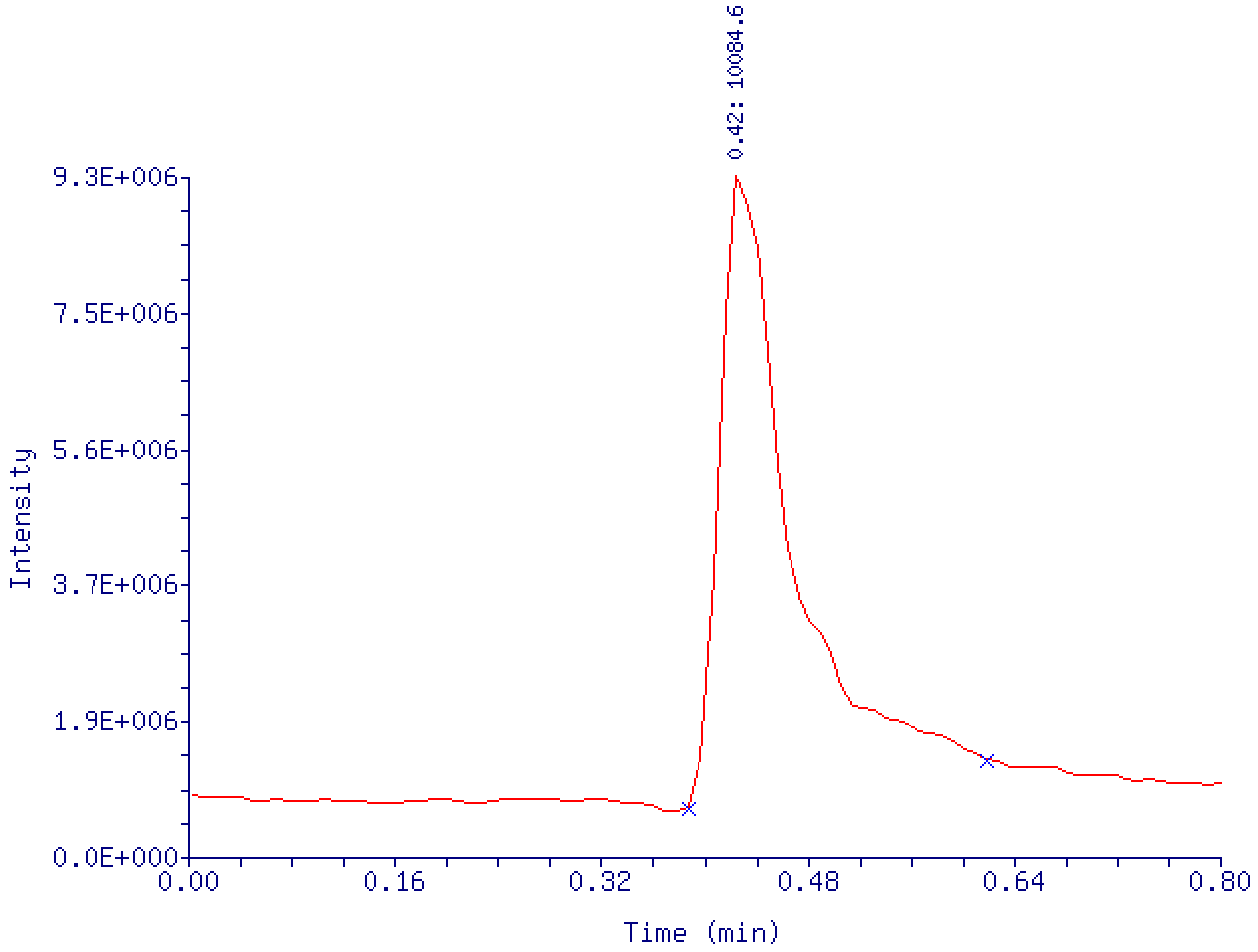

[<<] [Top] [Deconvolution] [Zoom Deconvolution] [Deconvolution Peak Report] [View Data] [Log File]

ESI Mass Spectrum of F+T01, RT = 0.42 min:

Scan Mode: - c ESI Full ms

Scans Averaged: 42-51 Minus: 17-38, 67

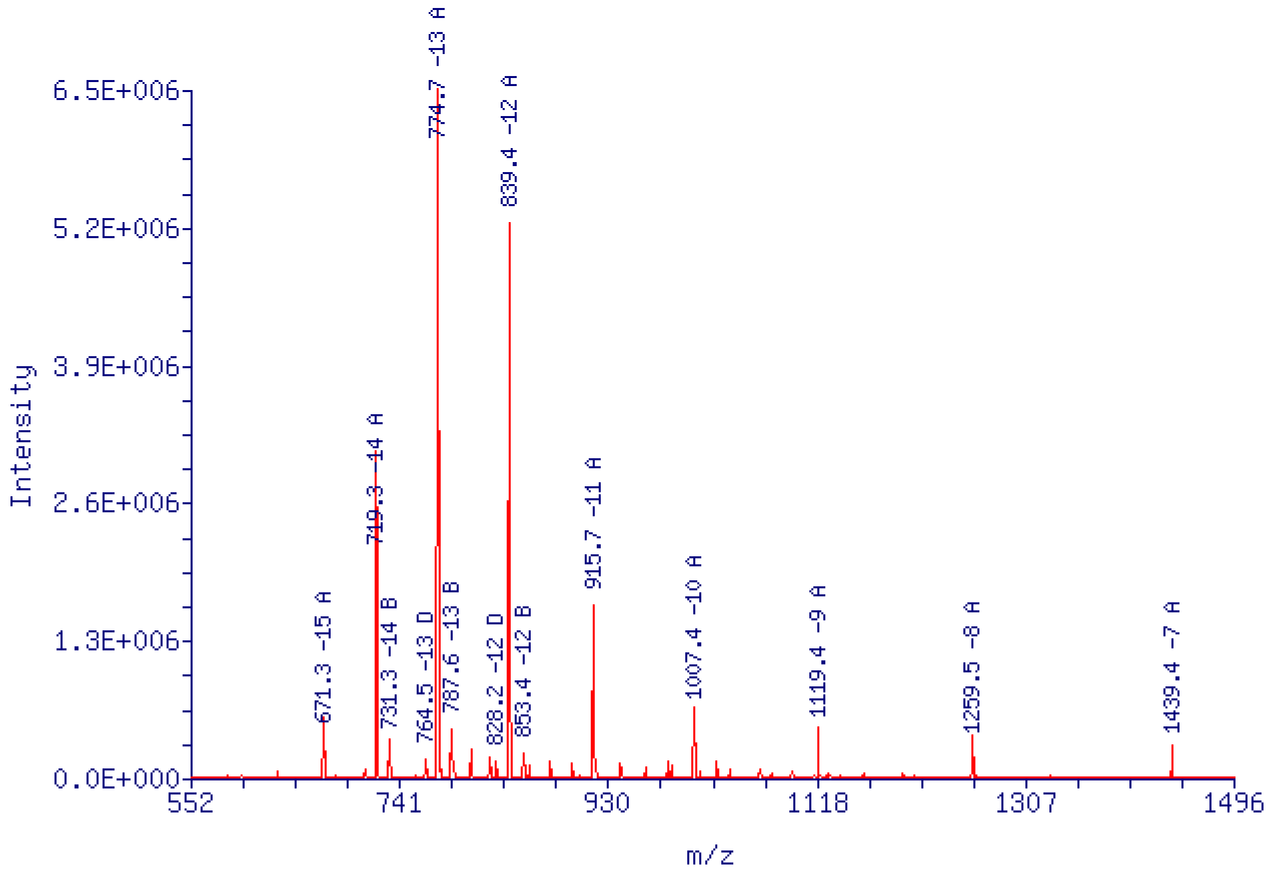

[<<] [Top] [ESI Mass Spectrum] [Zoom Deconvolution] [Deconvolution Peak Report] [View Data] [Log File]

Deconvoluted Mass Spectrum of F+T01, RT = 0.42 min:

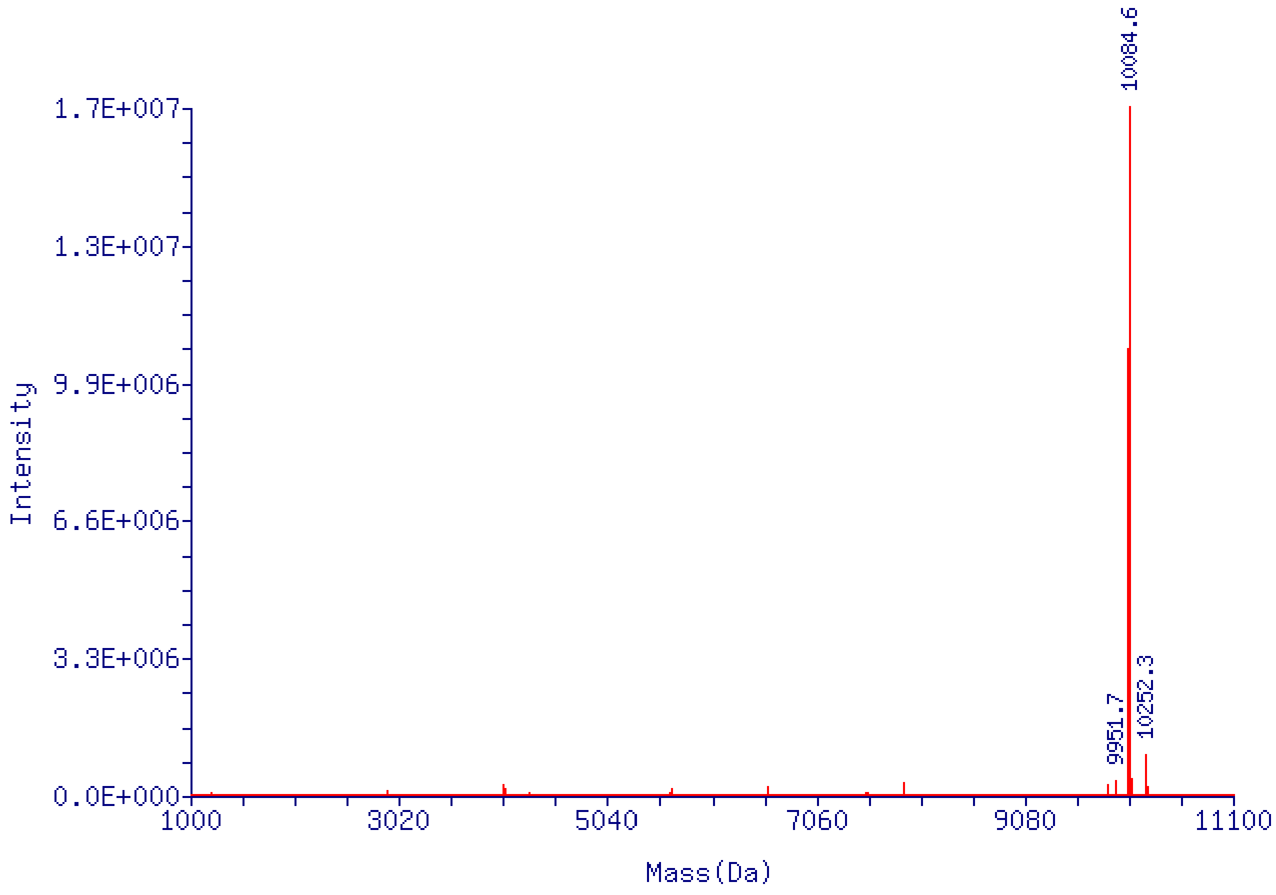

[<<] [Top] [ESI Mass Spectrum] [Deconvolution] [Deconvolution Peak Report] [View Data] [Log File]

Zoom Deconvoluted Mass Spectrum of F+T01, RT = 0.42 min:

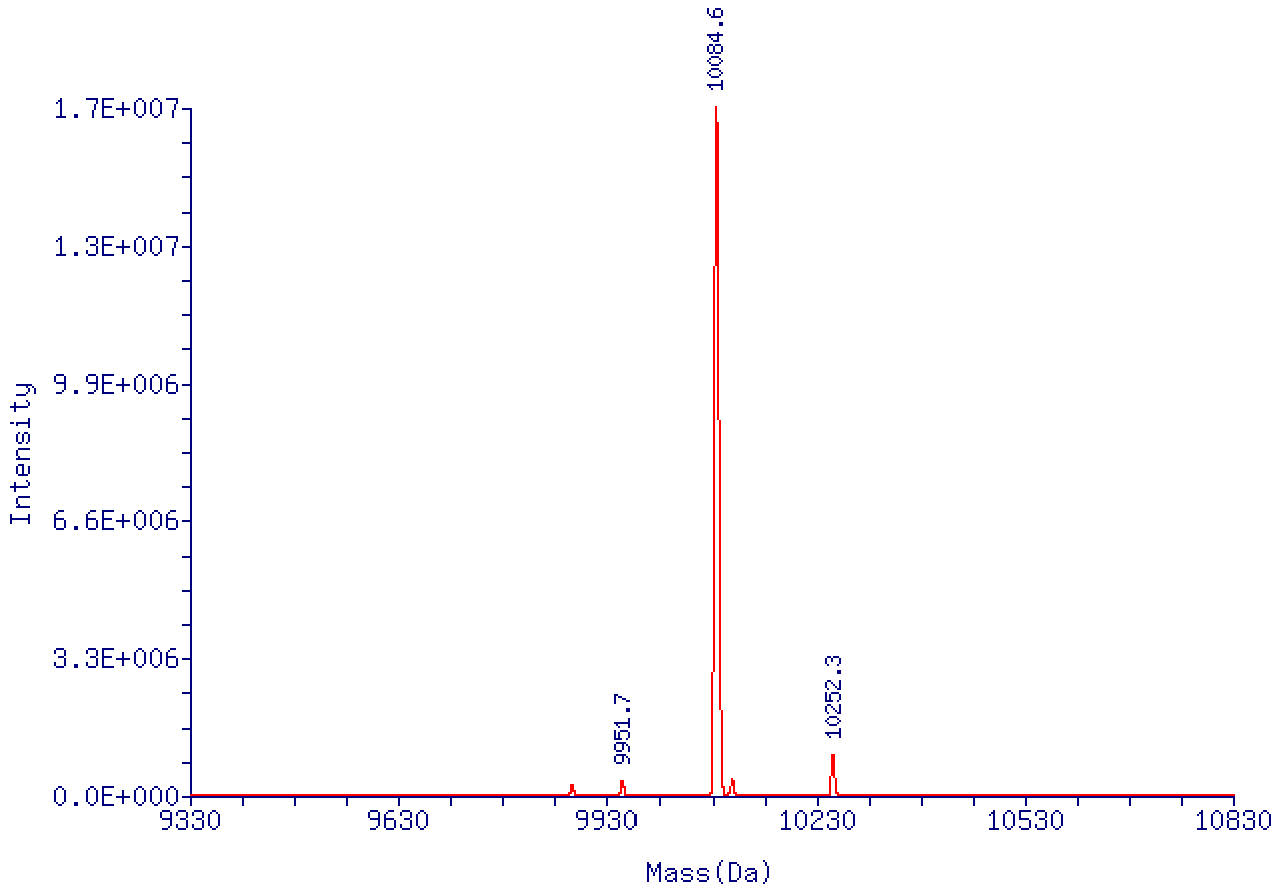

[<<] [Top]

Result Code	Indication
	Target mass found in chromatogram as the most abundant component within 0.040% mass error tolerance
	Target mass found as a major component or as a minor component with other target masses, but NOT as most abundant component in chromatogram
	Target mass found in chromatogram with either or all of the following:<br>(a) other non-target components present in spectrum > 30% abundance<br>(b) low spectral quality (low intensity and/or score)
	Target mass found in chromatogram, but NOT as the most abundant in any of the chromatographic peaks
	Target mass NOT found in chromatogram within 0.040% mass error tolerance
	No target masses specified

ZNova 3.0.2 ©2001-2013 Novatia, LLC

[<<]
Data File: E:\LTQ6\2023\0409\HPLC\H-20\1937422810-1.raw
Acquisition Date: 4/9/2023
Sample Name: TTTTTAAAAAAAAAAAAAAAAAAAAAAAAA
Sample ID: F+T01
Position: 8
Inj Vol: 30
Instrument Method: C:\Xcalibur\methods\oligo_htcs
Processing Method: C:\Xcalibur\methods\oligo_htcs

ProMass

Target Mass Summary

RT (min)	Target Mass (Da)	Observed Mass (Da)	Mass Error	Intensity	% Abundance (in Spectrum)	%Purity (Estimate)	Identity	Result Code
0.42	10064.9	10066.5	1.6 Da (0.016 %)	8.02E+006	73.71	77.80	Target Mass	

Chromatogram Summary

RT (min)	Base Peak Mass (Da)	Intensity	Spectral Quality	LC/MS Peak Area	LC/MS Area Percent
0.42	10066.5	8.02E+006	ok	2.34E+007	100.00

[<<] [Top]
LC/MS Chromatogram of F+T01:
TIC

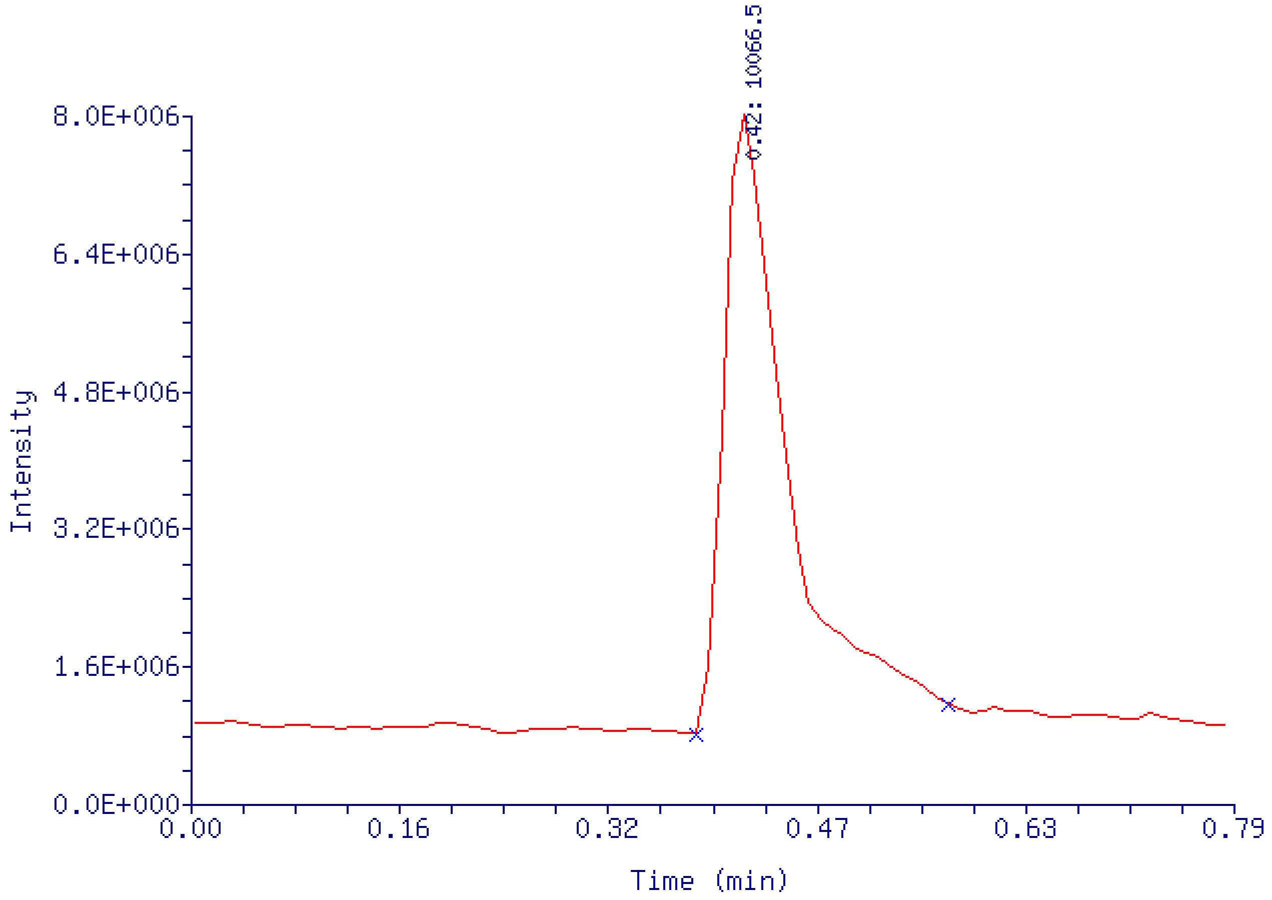

[<<] [Top] [Deconvolution] [Zoom Deconvolution] [Deconvolution Peak Report] [View Data] [Log File]

ESI Mass Spectrum of F+T01, RT = 0.42 min:

Scan Mode: - c ESI Full ms

Scans Averaged: 42-51 Minus: 17-39, 67

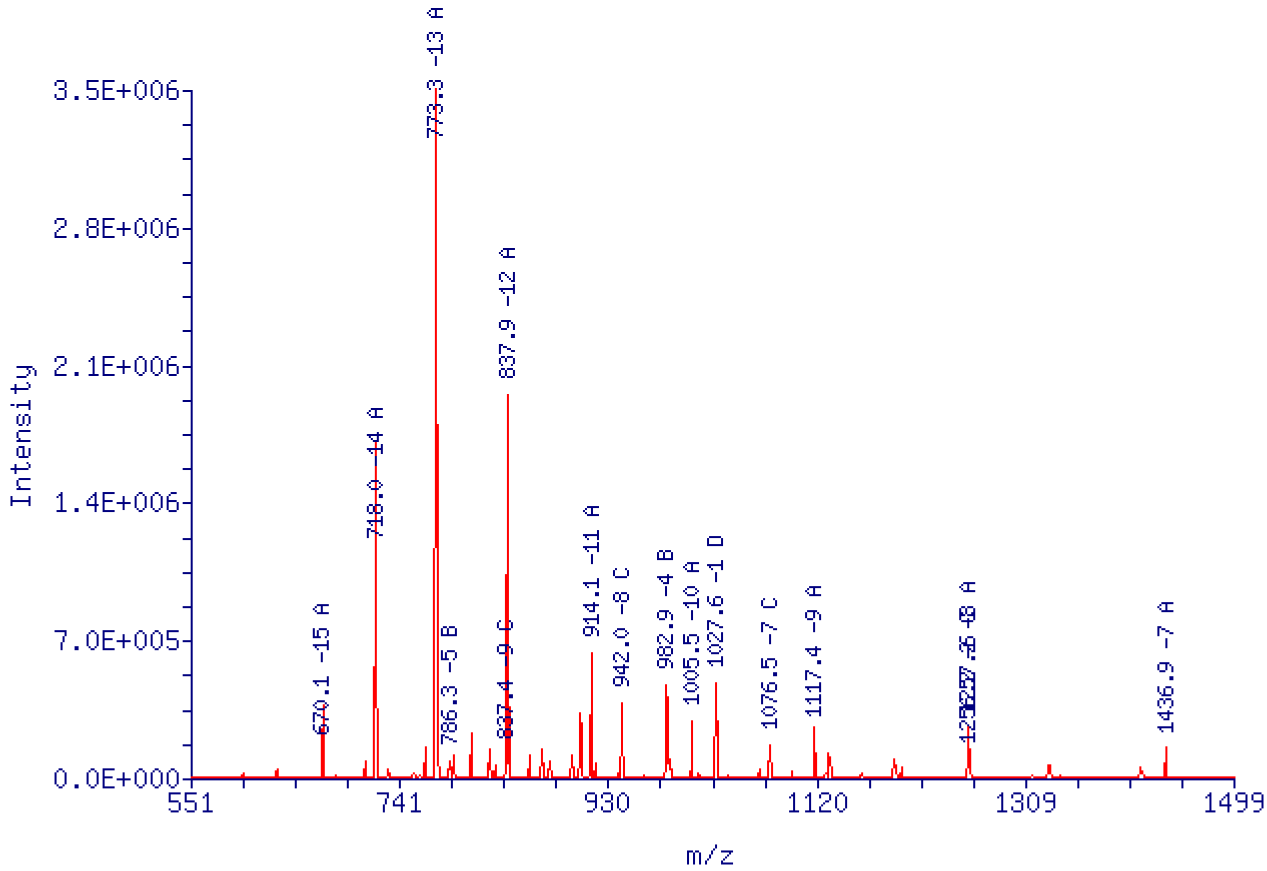

[<<] [Top] [ESI Mass Spectrum] [Zoom Deconvolution] [Deconvolution Peak Report] [View Data] [Log File]

Deconvoluted Mass Spectrum of F+T01, RT = 0.42 min:

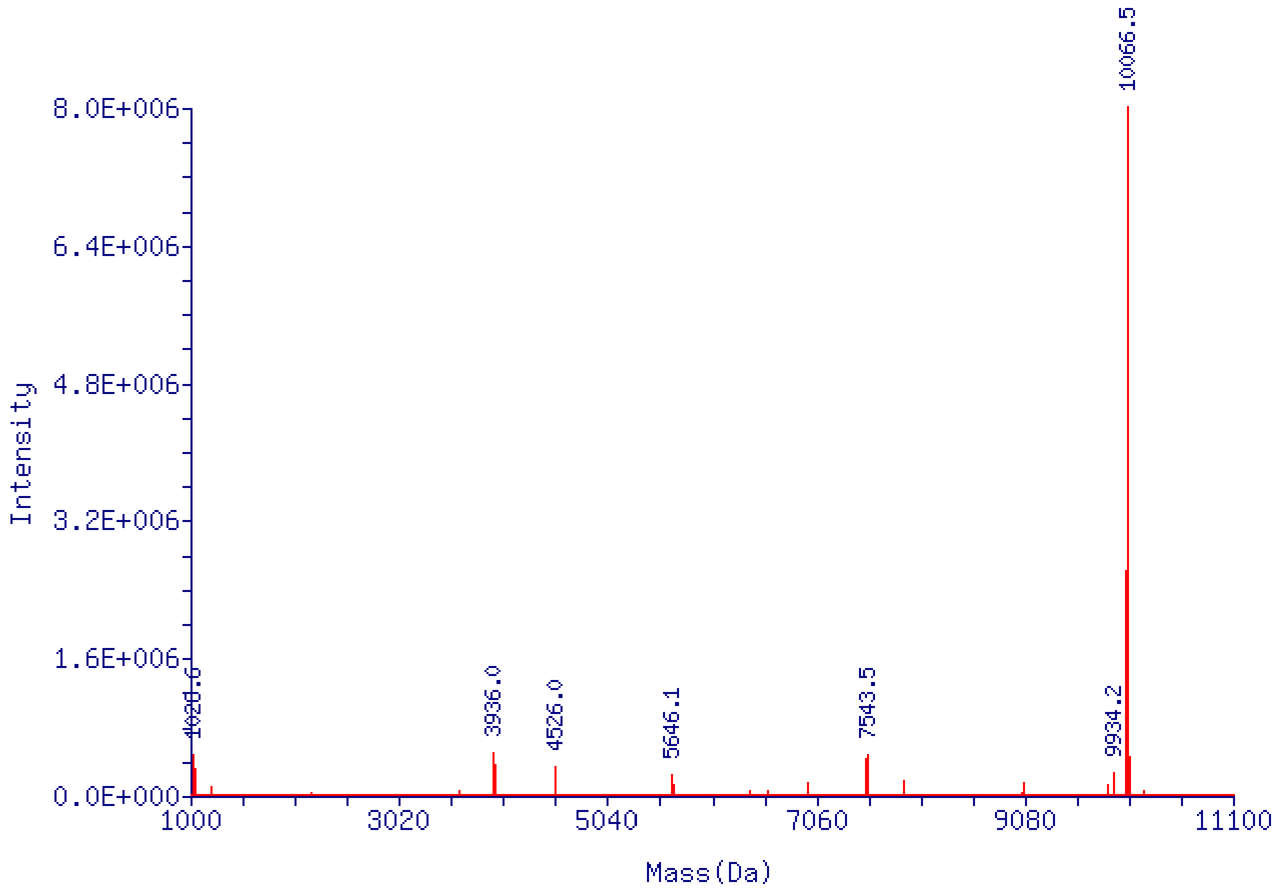

[<<] [Top] [ESI Mass Spectrum] [Deconvolution] [Deconvolution Peak Report] [View Data] [Log File]

Zoom Deconvoluted Mass Spectrum of F+T01, RT = 0.42 min:

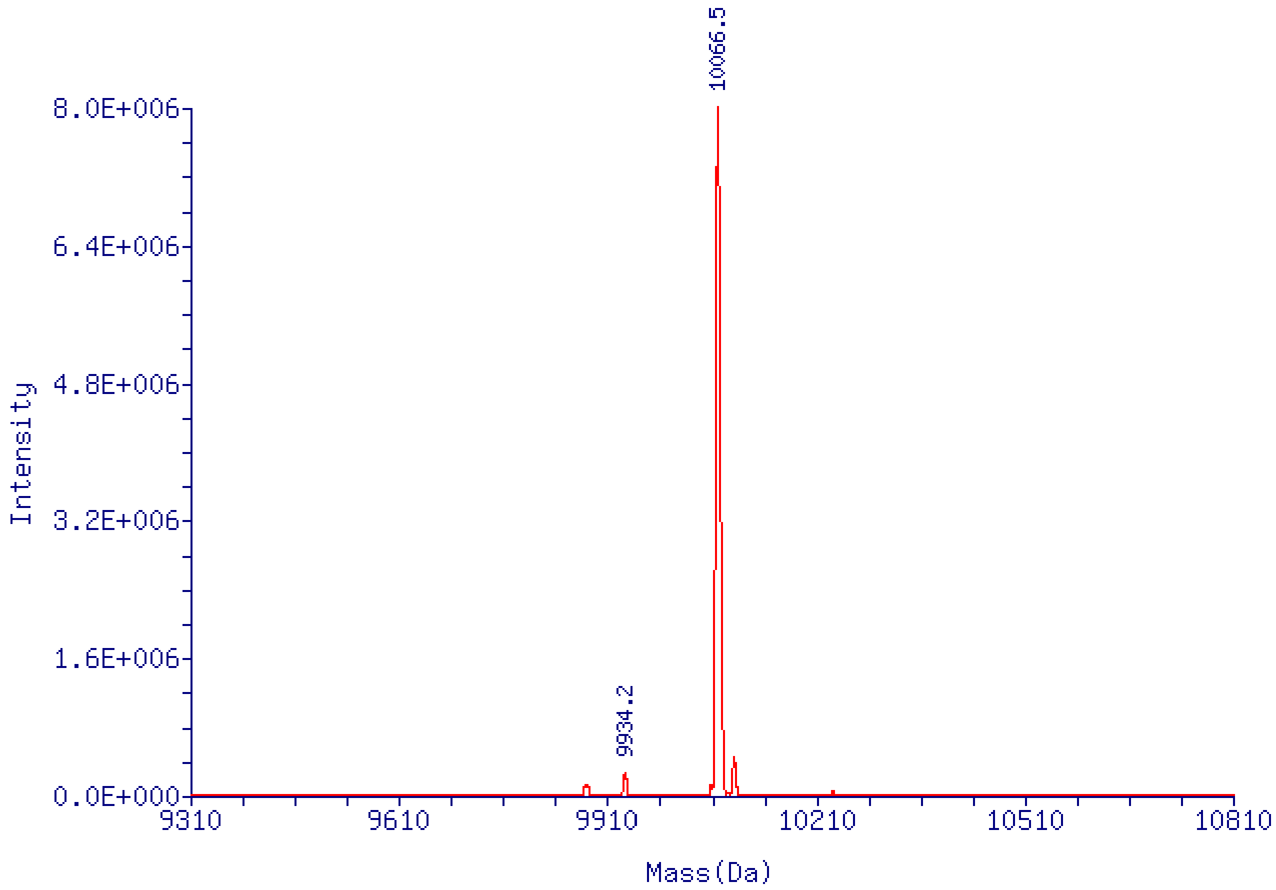

[<<] [Top]

Result Code	Indication
	Target mass found in chromatogram as the most abundant component within 0.040% mass error tolerance
	Target mass found as a major component or as a minor component with other target masses, but NOT as most abundant component in chromatogram
	Target mass found in chromatogram with either or all of the following:<br>(a) other non-target components present in spectrum > 30% abundance<br>(b) low spectral quality (low intensity and/or score)
	Target mass found in chromatogram, but NOT as the most abundant in any of the chromatographic peaks
	Target mass NOT found in chromatogram within 0.040% mass error tolerance
	No target masses specified

ZNova 3.0.2 ©2001-2013 Novatia, LLC
